# Supplementary material for: Lessons from COVID-19 syndromic surveillance through emergency department activity: a prospective time series study from western Switzerland
Source: BMJ Open. 2022 May 6;12(5):e054504. doi: 10.1136/bmjopen-2021-054504 (PMC9082728; doi:10.1136/bmjopen-2021-054504)
Supplement: Supplementary data [file bmjopen-2021-054504supp001.pdf]

Web Appendix

eSupplement 1. Method for Early Aberration Reporting System (STATA computing)

1. Identify the flu-like syndrome in emergency department software using the main complaint reported by the patient: combine the flu-like syndrome or fever/cough/shortness of breath and isolation droplet depending on the main complaint collected.

```
.gen flu_like_syndrome=1 if fever==1 | shortness_breath==1 | cough==1 & isolation_droplet
```

2. Extract the number of flu-like syndromes by day and the total number of patients attending the ED by day.

```
.collapse (sum) flu_like_syndrome total_ed id, by(date_admission)
```

3. Perform a moving average for the number of flu-like syndromes by day (7 days or less).

```
.tssmooth ma flu_like_sd_ma= flu_like_syndrome, window(7 1 7)

.tssmooth ma total_ed_ma= total_ed, window(7 1 7)
```

4. Plot the P-chart (using the usual limit Pr ± $3\sqrt{\frac{\mathrm{Pr}(1-\mathrm{Pr})}{N}}$).

```
. pchart flu_like_sd_ma date_admission total_ed_ma, sta recast(line) ytitle("Daily Flu-like syndrome in the ED",size(small)) title("P-chart moving average fraction defective", size(small)) xtitle("date",size(small)) ylabel(, labsize(small) axis(1)) ylabel(, labsize(small))
```

eSupplement 2. Correlogram between ICU occupancy and surveillance indicators at Lausanne University Hospital.

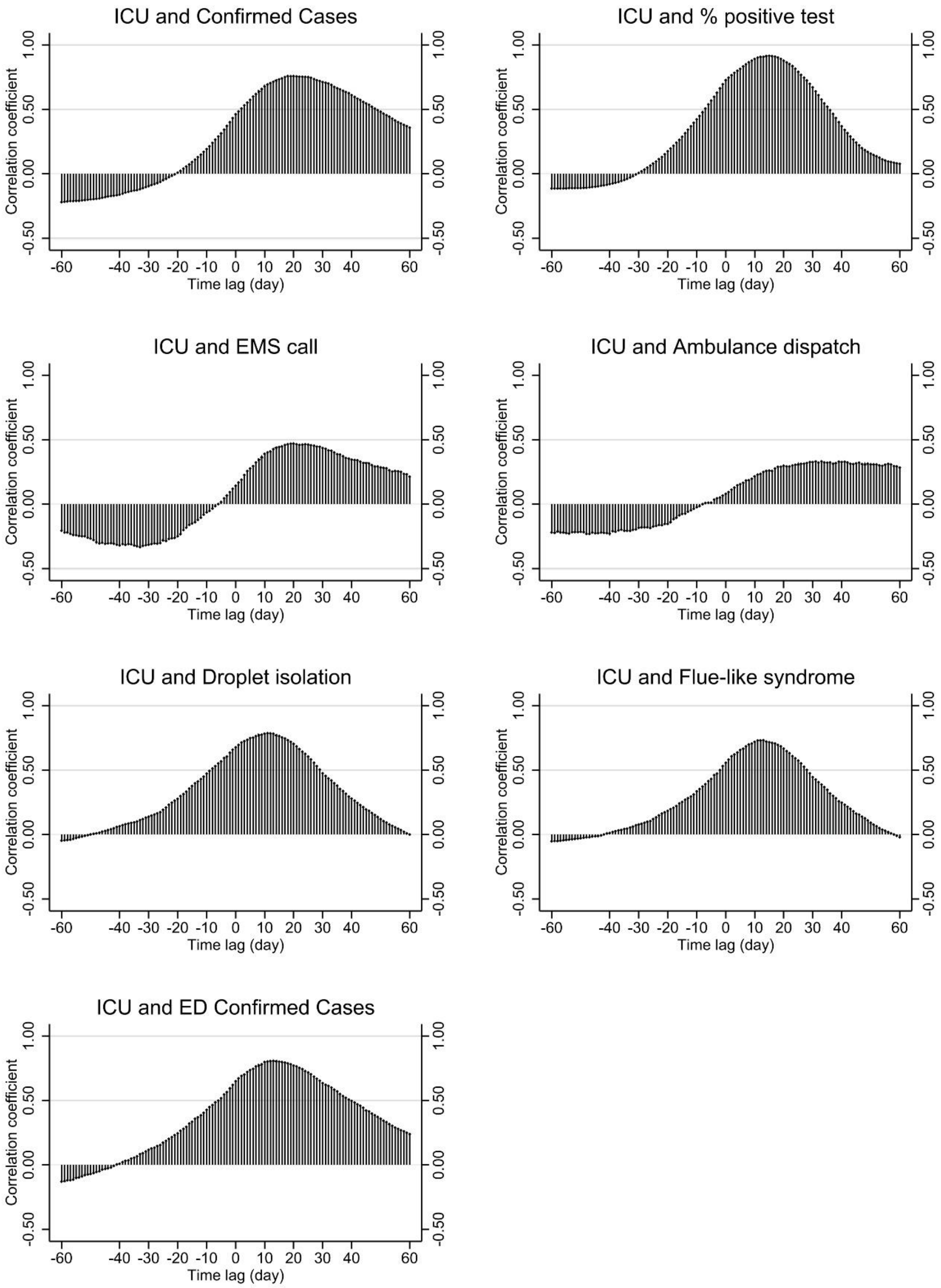

eSupplement 3. Daily P-charts of emergency department flu-like syndrome during the second wave of the COVID-19 outbreak at Lausanne University Hospital.

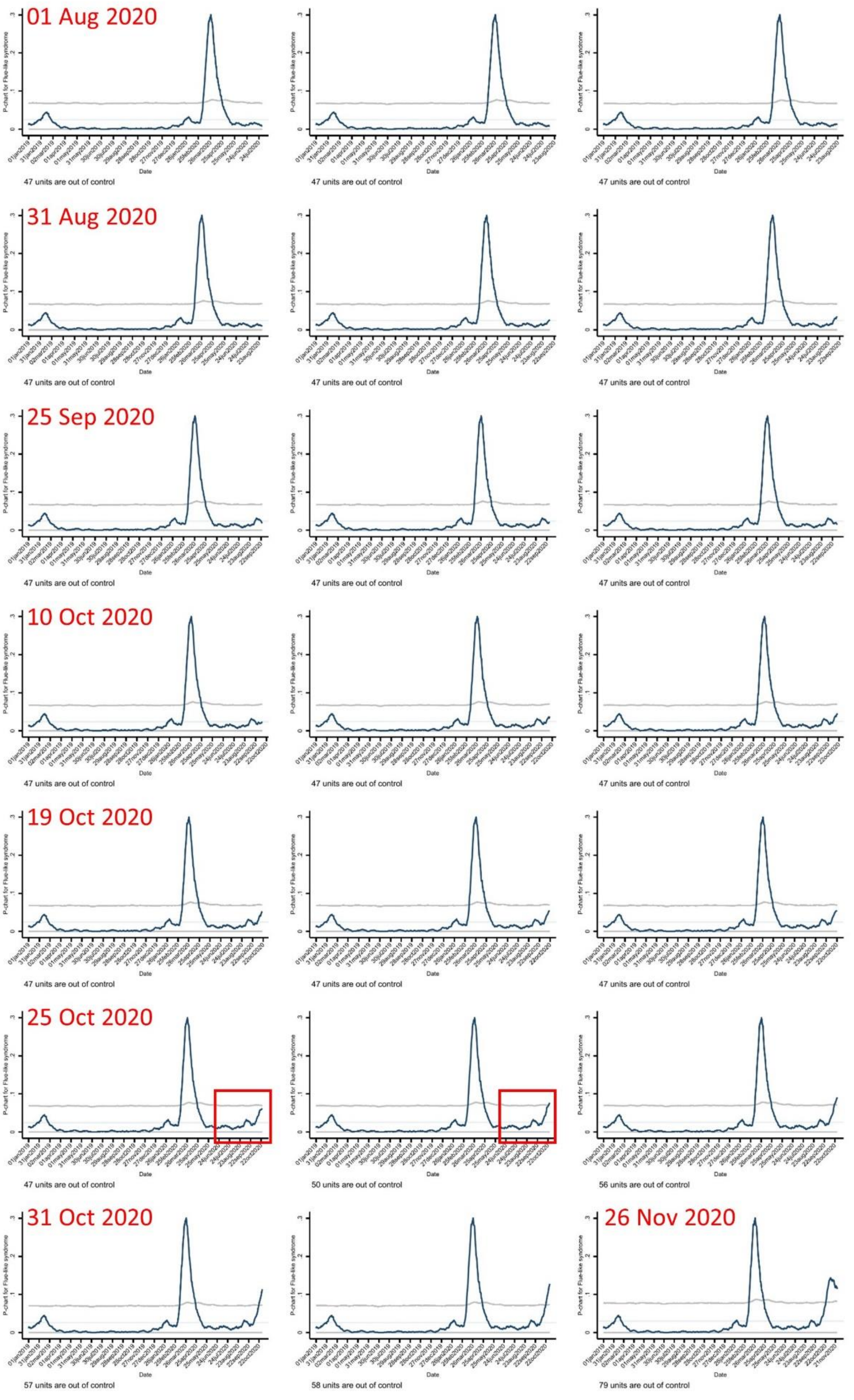

Ageron F-X, *et al. BMJ Open* 2022; 12:e054504. doi: 10.1136/bmjopen-2021-054504

eSupplement 4. Time series by daily ED specific activity at Lausanne University Hospital (trauma, cardiology and stroke).

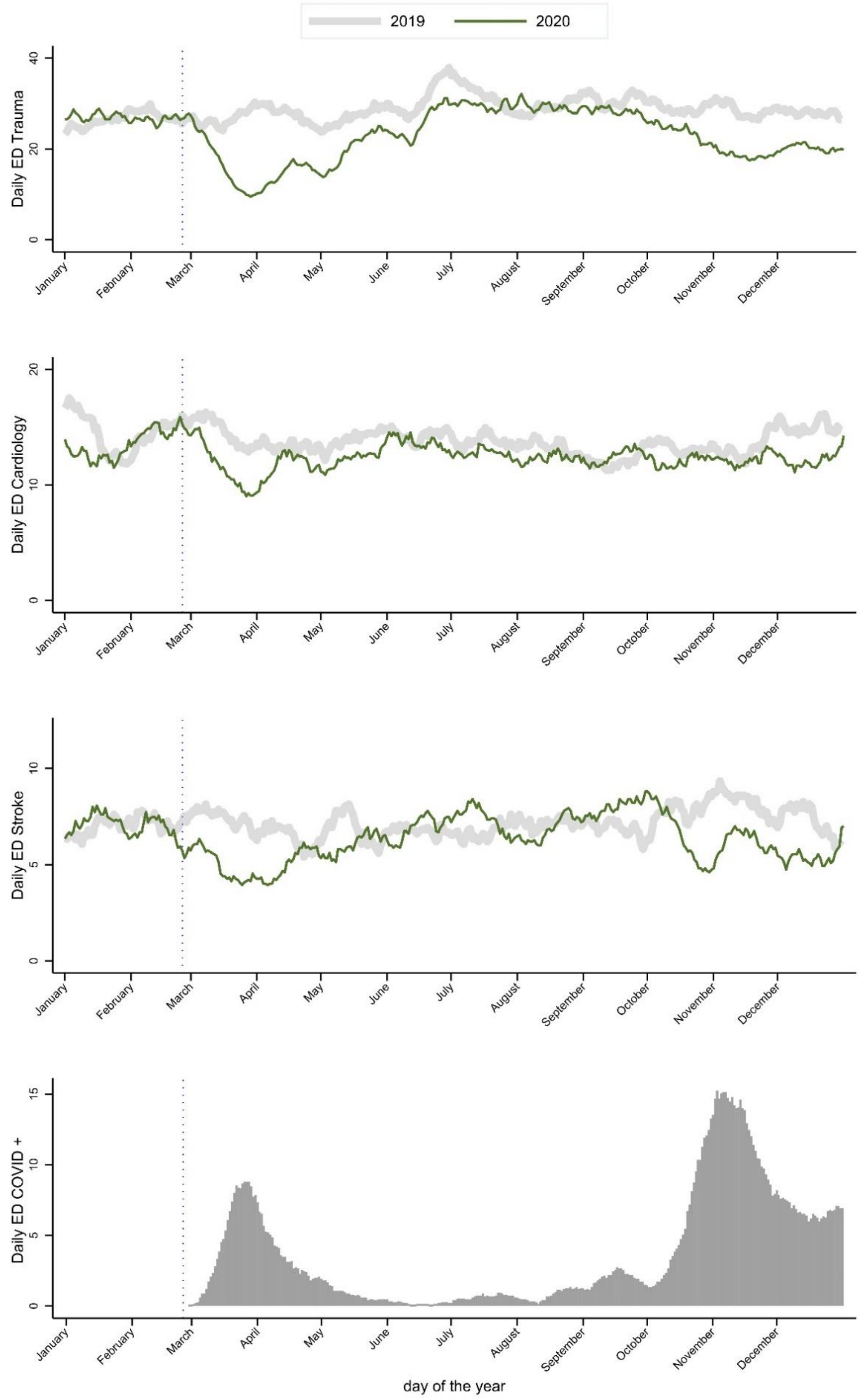

Ageron F-X, *et al. BMJ Open* 2022; 12:e054504. doi: 10.1136/bmjopen-2021-054504

eSupplement 5. Time series by daily ED specific activity at Lausanne University Hospital (GIB, diabetes, allergy).

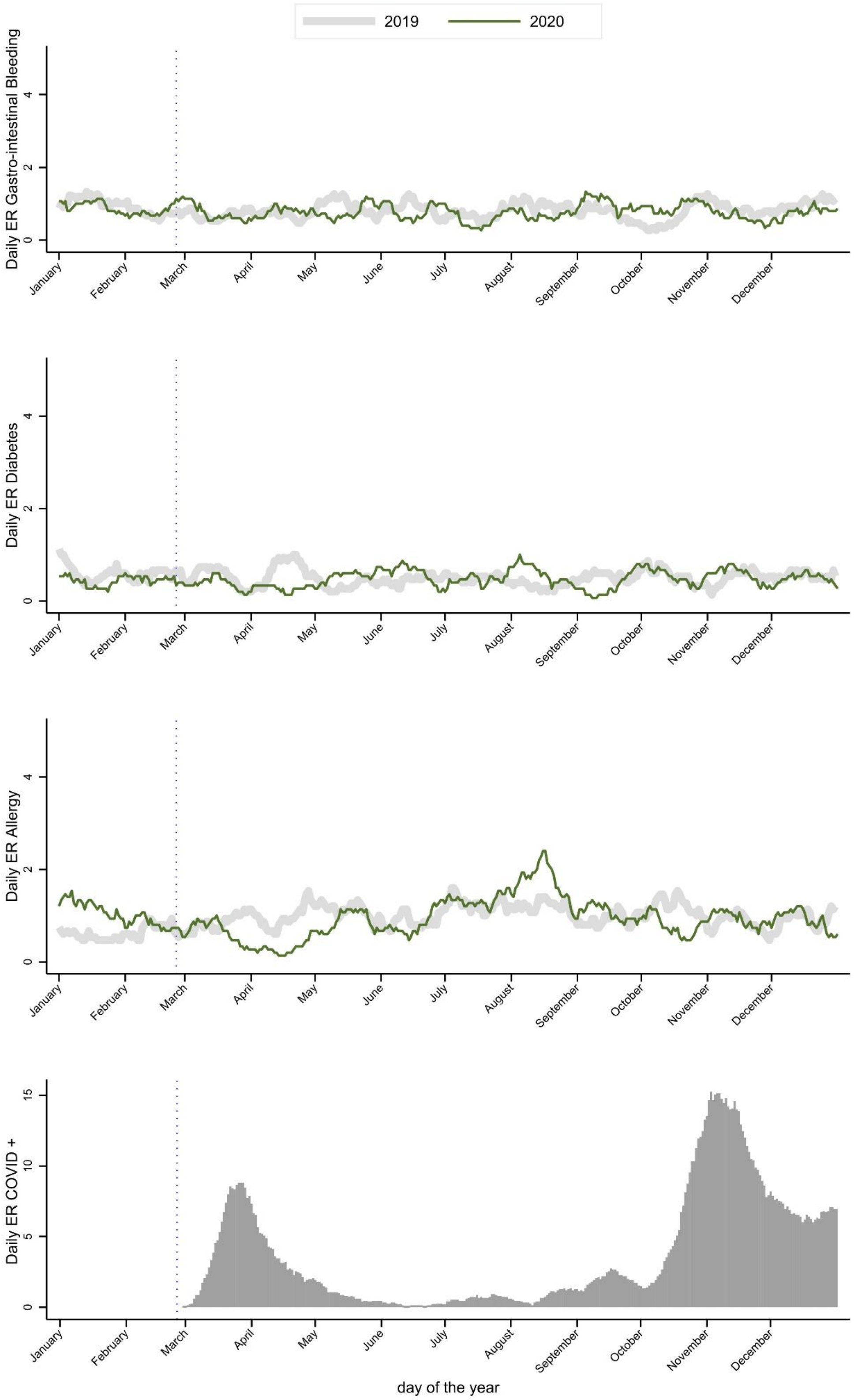

5

eSupplement 6. Time series by ED length of stay and waiting time at Lausanne University Hospital.

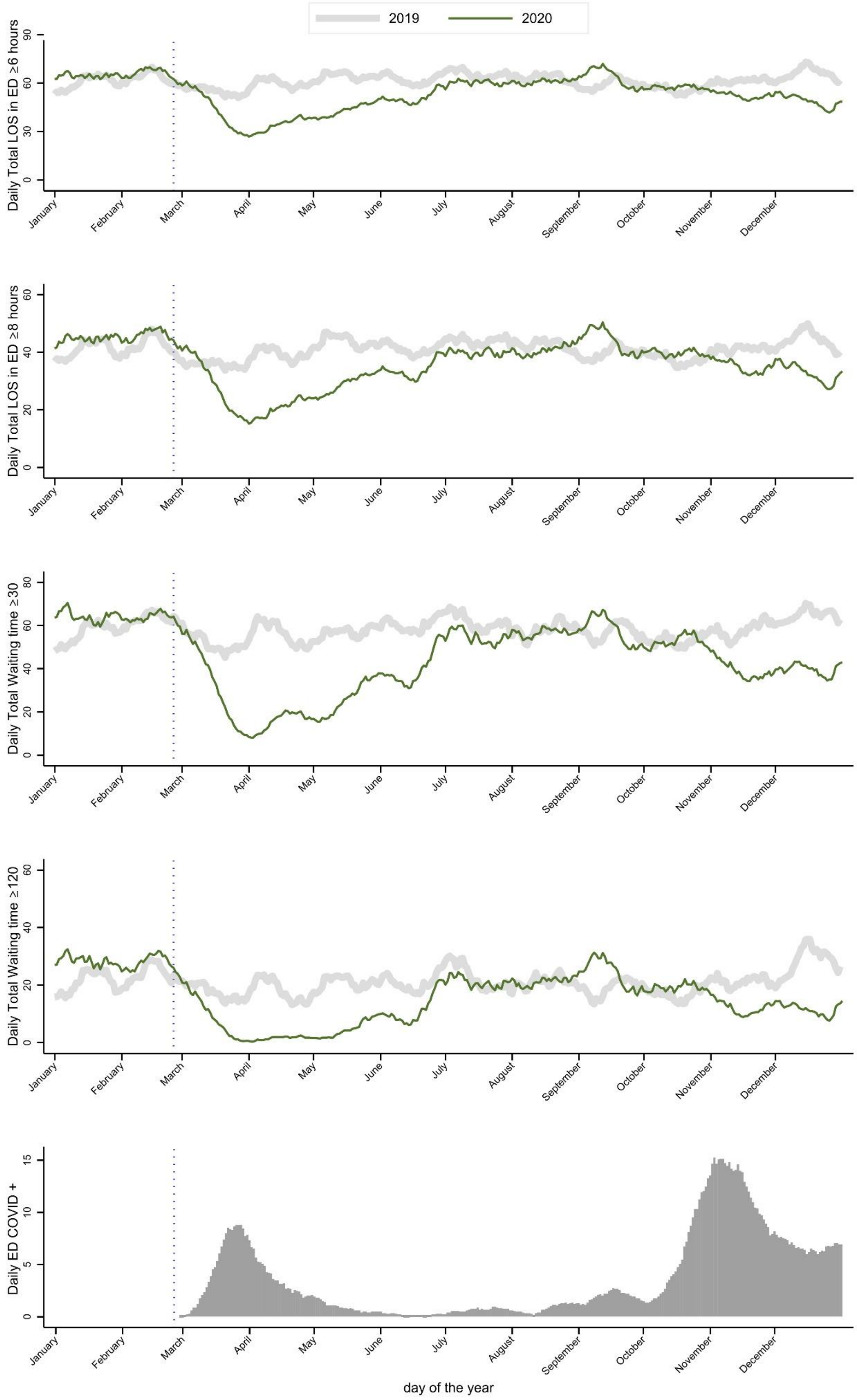

Ageron F-X, *et al. BMJ Open* 2022; 12:e054504. doi: 10.1136/bmjopen-2021-054504
