# Supplementary material for: Lessons from COVID-19 syndromic surveillance through emergency department activity: a prospective time series study from western Switzerland
Source: BMJ Open. 2022 May 6;12(5):e054504. doi: 10.1136/bmjopen-2021-054504 (PMC9082728; doi:10.1136/bmjopen-2021-054504)
Supplement: Author's manuscript [file bmjopen-2021-054504.draft_revisions.pdf]

BMJ Open

BMJ Open is committed to open peer review. As part of this commitment we make the peer review history of every article we publish publicly available.

When an article is published we post the peer reviewers' comments and the authors' responses online. We also post the versions of the paper that were used during peer review. These are the versions that the peer review comments apply to.

The versions of the paper that follow are the versions that were submitted during the peer review process. They are not the versions of record or the final published versions. They should not be cited or distributed as the published version of this manuscript.

BMJ Open is an open access journal and the full, final, typeset and author-corrected version of record of the manuscript is available on our site with no access controls, subscription charges or pay-per-view fees (http://bmjopen.bmj.com).

If you have any questions on BMJ Open's open peer review process please email info.bmjopen@bmj.com

BMJ Open

Lessons from COVID-19 syndromic surveillance through emergency department activity: a prospective time-series study from western Switzerland

Journal:	*BMJ Open*
Manuscript ID	bmjopen-2021-054504
Article Type:	Original research
Date Submitted by the Author:	14-Jun-2021
Complete List of Authors:	AGERON, Francois-Xavier; Lausanne University Hospital, Emergency Department<br>Hugli, Olivier; Lausanne University Hospital, Emergency department<br>Dami, Fabrice; Lausanne University Hospital, Emergency Department<br>Caillet-Bois, David; Lausanne University Hospital, Emergency Department<br>Pittet, Valerie; Centre Hospitalier Universitaire Vaudois, Institute of Social and Preventive Medicine<br>Eckert, Philippe; Lausanne University Hospital<br>Beysard, Nicolas; Lausanne University Hospital, Emergency<br>Carron, Pierre-Nicolas; Lausanne University Hospital, Emergency
Keywords:	COVID-19, Public health < INFECTIOUS DISEASES, ACCIDENT & EMERGENCY MEDICINE

SCHOLARONE™
Manuscripts

I, the Submitting Author has the right to grant and does grant on behalf of all authors of the Work (as defined in the below author licence), an exclusive licence and/or a non-exclusive licence for contributions from authors who are: i) UK Crown employees; ii) where BMJ has agreed a CC-BY licence shall apply, and/or iii) in accordance with the terms applicable for US Federal Government officers or employees acting as part of their official duties; on a worldwide, perpetual, irrevocable, royalty-free basis to BMJ Publishing Group Ltd ("BMJ") its licensees and where the relevant Journal is co-owned by BMJ to the co-owners of the Journal, to publish the Work in this journal and any other BMJ products and to exploit all rights, as set out in our licence.

The Submitting Author accepts and understands that any supply made under these terms is made by BMJ to the Submitting Author unless you are acting as an employee on behalf of your employer or a postgraduate student of an affiliated institution which is paying any applicable article publishing charge ("APC") for Open Access articles. Where the Submitting Author wishes to make the Work available on an Open Access basis (and intends to pay the relevant APC), the terms of reuse of such Open Access shall be governed by a Creative Commons licence – details of these licences and which Creative Commons licence will apply to this Work are set out in our licence referred to above.

Other than as permitted in any relevant BMJ Author's Self Archiving Policies, I confirm this Work has not been accepted for publication elsewhere, is not being considered for publication elsewhere and does not duplicate material already published. I confirm all authors consent to publication of this Work and authorise the granting of this licence.

For peer review only

Lessons from COVID-19 syndromic surveillance through emergency department activity: a prospective time-series study from western Switzerland

François-Xavier Ageron[1], Oliver Hugli[1], Fabrice Dami[1], David Caillet-Bois[1], Valérie Pittet[2], Philippe Eckert[3], Nicolas Beysard[1], Pierre-Nicolas Carron[1]

1 Emergency Department, Lausanne University Hospital, 46 Rue du Bugnon, 1011 Lausanne, Switzerland

2 Centre for Primary Care and Public Health, 10 route de la Corniche, University of Lausanne, Lausanne, Switzerland

3 General Directorate, Lausanne University Hospital, 21 Rue du Bugnon, 1011 Lausanne, Switzerland

Submitted to: *BMJ open*

Word count: 2678; 3 figures; 2 tables

Corresponding author:

Dr. Francois-Xavier AGERON, MD, PhD

Emergency Department

Lausanne University Hospital

46 Rue du Bugnon

1011 Lausanne, Switzerland

Tel.: +41 79 556 88 69

E-mail: francois-xavier.ageron@chuv.ch

For peer review only - http://bmjopen.bmj.com/site/about/guidelines.xhtml

Abstract

Objective: We aimed to assess if emergency department (ED) syndromic surveillance during the first and second waves of the COVID-19 outbreak could have improved our surveillance system.

Design, settings: We did an observational study using aggregated data from the ED of a university hospital and public health authorities in western Switzerland.

Participants: All patients admitted at the ED were included.

Primary outcome measure: The main outcome was intensive care unit (ICU) occupancy. We used time series methods for ED syndromic surveillance (flu-like syndrome, droplet isolation) and usual indicators from public health authorities (new cases, proportion of positive test in the population).

Results: Based on 37319 ED visits during the COVID-19 outbreak, 1421 ED visits (3.8%) were positive for severe acute respiratory syndrome coronavirus 2 (SARS-CoV-2). Patients with flu-like syndrome or droplet isolation in the ED showed a similar correlation to ICU occupancy as confirmed cases in the general population with a time lag of approximately 13 days (0.73, 95% CI 0·64-0.80; 0.79, 95% CI 0.71-0.86; and 0.76, 95% CI 0.67-0.83, respectively). The proportion of positive tests in the population showed the best correlation with ICU occupancy (0.95, 95% CI 0.85-0.96).

Conclusion: ED syndromic surveillance is an effective tool to detect and monitor a COVID-19 outbreak and to predict hospital resource needs, and would have allowed to anticipate ICU occupancy by 13 days, including detection of significant aberration at the beginning of the second wave.

Keywords: emergency department, COVID-19, surveillance system, public health

For peer review only

Article summary

Strengths and limitations of this study

- This observational study showed that Emergency department syndromic surveillance presents a reliable correlation with confirmed new cases in the general population during the first and second waves of the COVID-19 outbreak.
- We used rigorous method to detect significant early signal of the second waves and to predict ICU occupancy with a time lag of two weeks.
- As the Emergency department stands at the interface between the community and the hospital, Emergency department syndromic surveillance represents real-time monitoring tool and strategic information for health care authorities and policy-makers.
- Surveillance data could be difficult to interpret at the initial stage of a new pandemic and studies to assess the ability of ED surveillance systems to detect a potential new threat need to be performed prospectively in real time.

For peer review only

For peer review only - http://bmjopen.bmj.com/site/about/guidelines.xhtml

Introduction

In early 2020, the World Health Organisation declared the COVID-19 outbreak to be a public health emergency of international concern.[1] Europe was particularly badly hit in spring 2020. After a relative lull in the summer, a second wave occurred in Europe in autumn 2020. Switzerland was among the most affected countries during this period with a much higher COVID-19 incidence compared to the first wave and with a 7-day incidence higher than 600 confirmed cases per 100,000.[2–4] Even if the second wave was expected, its beginning, timing and magnitude were not fully anticipated by the public health authorities.

Current public health surveillance systems include laboratory tests, death rates, hospital-based surveillance, and sentinel networks in primary care. Usual surveillance reports use the notification rate of confirmed cases and deaths, laboratory tests, hospital and intensive care unit (ICU) admission and occupancy rates. Primary care sentinel surveillance collects syndromic symptoms related to seasonal flu-like syndrome. Emergency departments (ED) are uniquely positioned at the interface of the community and hospitals and could serve as early warning systems to identify emerging threats and support decisions of public health authorities.[5] However, only one-third of European countries include ED data in their syndromic surveillance.[6] Until now, this has not been the case in Switzerland where ED data sets have not been used to detect or monitor epidemic outbreaks and, more specifically, the COVID-19 outbreak.

From July to early September 2020, most European countries observed an increase in the incidence of COVID-19 in young people <35 years, but without any significant simultaneous increase in hospital and ICU occupancy. These two concurrent numbers potentially contributed to erroneously reassure public health and political authorities. In addition, a retrospective analysis indicated a persistent higher incidence at the end of the summer in Switzerland, particularly in the western region.[7] However, it remains unknown if the monitoring of cases admitted to the ED would have provided early predictive clues on the resurgence of the pandemic. The aim of our study was to assess if ED

For peer review only

For peer review only - http://bmjopen.bmj.com/site/about/guidelines.xhtml

syndromic surveillance during the first and second waves of the COVID-19 outbreak could have improved health surveillance and provided additional information for the earlier detection of outbreak signals.

Methods

Study design and population

We did an observational study to assess whether ED syndromic surveillance would have improved the management of the first and second waves of the COVID-19 outbreak in a health system in western Switzerland, based on routine data from the Canton of Vaud and Lausanne University Hospital.

We used aggregated data from the ED of Lausanne University Hospital, one of the five university hospitals in Switzerland, located in the French-speaking region. It serves as a primary care hospital for the Lausanne area with a population of 250,000 inhabitants and as a tertiary hospital for western Switzerland with a population of 1 million inhabitants. The ED triage includes approximatively 65,000 adult patients per year, two-thirds of whom are admitted to the ED, and one-third to the primary care consultation.

We used data from all consecutive visits leading to ED admission from 25 Feb, 2019 to 19 Jan, 2020 (pre-COVID period; used as a control period) and from 25 Feb, 2020 (date of the first infection due to severe acute respiratory syndrome coronavirus 2 [SARS-CoV-2] in Switzerland) to 25 Feb, 2021. Patients referred to the primary care consultation after ED triage were excluded. We also considered aggregated data for the entire population of the Canton of Vaud collected by the emergency medical service (EMS) dispatch centre and the public health authorities.

Data collection

For peer review only

For peer review only - http://bmjopen.bmj.com/site/about/guidelines.xhtml

We collected aggregated data from the ED including date and hour of admission, age group in categories, gender, main complaints at admission classified using the Swiss Emergency Triage Scale (SETS),[8] deaths in the ED, hospital admissions to the ward or intensive care unit (ICU), and positive COVID-19 test notification. The modified early warning score (MEWS) and the national early warning score (NEWS) were calculated from the initial triage vital signs.[9,10] Data were extracted from the ED patient flow management software (Gyroflux®, Lausanne University Hospital, Switzerland) including triage vital signs, symptoms, isolation, ED length of stay, COVID test result, discharge diagnosis and destination of ED patients. Only patient flow aggregated data available in real time were collected, without additional data from medical records. In addition, data were collected from the EMS dispatch centre ("Centrale d'appels sanitaires d'urgence 144") of the Canton of Vaud, including daily emergency calls and ambulance dispatch. We also collected daily hospital occupancy for COVID-19 patients in general wards and the ICU, as well as data from the Vaud health authority surveillance system (notification of new cases) and laboratory surveillance (results of PCR and antigen tests).[11]

Outcome

We selected daily absolute ICU occupancy as the primary outcome. ICU beds are a scarce resource requiring trained staff and specific medical devices. The prediction and anticipation of critical care resources has been a key issue in the COVID-19 outbreak. We considered the absolute ICU bed occupancy and not the ICU occupancy rate as the total number of ICU beds regularly evolved during the pandemic, according to needs and available resources (i.e. an increase from 35 to 76 beds).

Surveillance indicators

We studied and compared "usual" and ED-specific surveillance indicators for COVID-19. Usual surveillance indicators were: 1) number of new confirmed cases of COVID-19 in the population (notification by cantonal public health authorities); and 2) laboratory surveillance with the proportion of positive tests (PCR and antigen) from all tests performed. ED surveillance indicators were: 1)

For peer review only - http://bmjopen.bmj.com/site/about/guidelines.xhtml

number of confirmed cases of COVID-19 during ED stay; 2) number of patients subjected to droplet isolation measures in the ED; 3) syndromic surveillance with flu-like syndrome in the ED at triage; 4) number of EMS calls; and 5) number of ambulance dispatches.

Data analysis

We applied time series analyses for ED COVID-19 visits and for syndromic surveillance, including infectious disease, respiratory disease, cardiac symptoms including chest pain, neurologic symptoms including acute paralysis, gastrointestinal bleeding, trauma, psychiatric disorders, and hyper- or hypoglycaemia. We plotted the time series of syndromic surveillance data during the COVID-19 period and compared these to the same period of 2019. We smoothed time-series curves based on the moving 7-day average. We compared graphically ED-EMS surveillance and usual surveillance in the general population and explored the relationship between ICU occupancy and ED-EMS surveillance and traditional surveillance indicators by cross-correlation, and plotted correlograms. We tested the correlation between time series using the Breusch-Godfrey test for higher-order serial correlation and Durbin's alternative test for serial correlation.[12,13] The time lag in days between surveillance indicators and ICU occupancy was determined by estimating which lag showed the highest correlation on correlograms. We performed a Vector Auto-Regression (VAR) model and considered the optimal time lag for the lowest final prediction error and the lowest Akaike's information criterion. We performed Granger-causality with a linear regression model and a VAR model to determine which indicator was the best to predict ICU occupancy.[14] Quality control charts were then used to detect early aberration in daily data. The Early Aberration Reporting System (EARS) uses different methods for temporal aberration detection, including the Shewhart chart (P-chart), moving average, and variation of the cumulative sum.[15,16] To assess the usefulness of the ED surveillance system, we assessed graphically the moving average for ED flu-like syndrome aberrations detected by P-chart during the second wave. The P-chart measures the fraction of

For peer review only - http://bmjopen.bmj.com/site/about/guidelines.xhtml

nonconforming units in a sample. The control limits for the P-chart were estimated using the formula: Pr ± $\sqrt{\frac{\mathrm{Pr}\,(1-\mathrm{Pr})}{N}}$ where Pr is the estimated fraction.

We did not reported any missing value for syndromic surveillance in the ED (mandatory item in the software).

Data were analysed using Stata version 16.0 (StataCorp, College Station, TX, USA).

Patient and Public involvement statement

Patients were not involved in the research question and in the design of the study.

Results

We collected 37319 ED visits from 25 Feb, 2020 to 25 Feb, 2021 (COVID-19 period) and 42584 ED visits from 25 Feb, 2019 to 19 Jan, 2020 (pre-COVID [control] period). We reported 1421 (3.8%) confirmed cases of COVID-19 during ED stay, 2181 (5.8%) flu-like syndromes, and 4124 (11.1%) ED visits with droplet isolation (table 1). An increase of flu-like syndromes was observed during the COVID-19 period. The frequency of ICU admission also increased during the COVID-19 period by 30% (OR 1.30; 95% CI 1.15-1.47; P<.001).

We plotted routine surveillance indicators (confirmed cases, laboratory surveillance and ICU occupancy) and emergency surveillance indicators (EMS and ED indicators) (figure 1). The frequency of positive laboratory tests and confirmed cases first immediately increased, followed by ED flu-like syndrome, ED isolation droplet, and confirmed ED COVID-19. All indicators followed exactly the trend in ICU occupancy with a time lag, depending on the indicators. ED flu-like syndrome and ED droplet isolation showed a higher increase in the first wave than the second wave compared to ED COVID-19-confirmed cases. All surveillance indicators, except the EMS total number of calls, showed a good correlation with ICU occupancy (table 2). Correlograms showed a positive correlation for all indicators during the second wave (eFigure 1 in the supplement). The highest correlations between

For peer review only - http://bmjopen.bmj.com/site/about/guidelines.xhtml

For peer review only

ED-EMS surveillance indicators and ICU occupancy were obtained with time lags of 10 to 13 days (table 2). A selection of daily P-charts for ED flu-like syndrome during the second wave are presented in figure 2. A significant aberration was detected as of the 25 Oct, 2021 (figure 2). Aberrations were detected three weeks before the maximum ICU occupancy was reached.

Daily ED activity is presented in figure 3. The total number of ED visits decreased during the first and second waves compared to ED activity the previous year. Hospital admission remained stable, with a slight increase during the second wave. The number of patients who presented an intermediate-to-high risk of critical care (NEWS ≥ 5) increased during the first and second waves. Compared to 2019, trauma, cardiology, and stroke activity decreased during the first wave and to a lesser extent during the second wave (eFigure 2 in the supplement). Gastrointestinal bleeding and diabetes were unchanged during both waves (eFigure 3 in the supplement). Allergy decreased during the spring lockdown and increased during the summer break. ED length of stay and waiting time decreased during the first wave (eFigure 4 in the supplement). During the second wave, ED length of stay decreased on a smaller scale.

Discussion

Our study shows the potential for ED syndromic surveillance as an effective tool to detect and monitor COVID-19 outbreaks and to predict hospital resource needs. The ED surveillance system correlated with ICU occupancy and would have allowed to anticipate ICU occupancy by 11 to 13 days. Of note, it would have also enabled the detection of significant aberration at the beginning of the second wave. In addition, ED surveillance would provide useful information to plan hospital bed needs, including the number and severity of patients admitted to the ED, hospital and ICU admissions, and hospital resources required for trauma, cardiology and neurology patients.

Comparison with other studies

For peer review only - http://bmjopen.bmj.com/site/about/guidelines.xhtml

Similar to others, we found a decrease in the total number of ED visits during the first wave.[17–20] Many countries implemented a lockdown during the first wave of the outbreak that explained the decrease in ED visits to a large extent.[21] Importantly, our syndromic surveillance results allow to describe with finer granularity the change in ED activity. During lockdown, we observed a decrease of certain diseases associated with exposure to environmental factors, such as allergy, or CO_2 emission. Jephcote et al. also reported a change in air quality during lockdown in the United Kingdom.[22] Kuitunen at al showed that the volume of road traffic and ED visits decreased at the same time, and we also observed a reduction in the number of minor and major traumatic injuries.[21] The COVID-19 outbreak well illustrated that a change in human activities contributing to pollution affect immediately population health.

We showed that ED surveillance data was accurate to detect changes in the epidemiology of the COVID-19 outbreak, based on our current system using syndromic flu-like presentations and isolation measures for droplet. In the USA, Pulia and al. showed that the surveillance of patients placed in respiratory isolation for an acute respiratory infection was useful to identify and monitor trends during the pandemic.[23] In Paris, researchers found that ED visits and EMS calls were correlated with ICU admission, as was the proportion of positive PCR tests.[24] We showed that ED surveillance predicts ICU occupancy with a time lag of 13 days and the proportion of positive laboratory tests with a lag of 15 days, the same lag as the Paris study.

Clinical implications

ED visits have constantly risen during the last decade and one-fourth to one-third of the population visit an ED annually.[25] EDs have become an important player in the public health system and an interface between primary care and the hospital. Indeed, the ED represents today almost the only clinical pathway to unscheduled in-hospital care. For this reason, the ED has the potential to become a real-time observatory of public health if properly designed with well-defined indicators. Consequently, it is not surprising that ED surveillance would have been an effective tool to detect

For peer review only - http://bmjopen.bmj.com/site/about/guidelines.xhtml

and monitor COVID-19 outbreak activity as it provides simple indicators for real-time monitoring that allow a rapid response from health care authorities.

Inside hospitals, ED surveillance would be useful to plan ICU or intermediate-care unit resources by predicting ICU occupancy with a significant time lag, specific to the epidemic. Additional syndromic surveillance for surgery and other medical specialities would also be helpful to reduce some activities and re-allocate resources where they are the most needed. Of note, ED surveillance would enable to detect the indirect consequences of a pandemic, such as the change in ED visits for life-threatening conditions. It is unlikely that myocardial infarction and strokes decreased during lockdown, but the decrease in chest pain and stroke symptoms observed in the ED suggests that patients avoided attending the ED as a consequence of the stay-at-home campaign and fear of nosocomial COVID-19 infection.[26] This type of ED surveillance data could incite health authorities to inform the population to alert emergency services in case of chest pain and stroke symptoms, regardless of the COVID-19 outbreak.

Strengths and weaknesses

Our study presents some strengths and weaknesses. First, we used a well-described cohort of consecutive ED patient visits without missing data for outcome, syndromic surveillance, and triage severity. Follow-up was complete. Second, we used simple observations to assess the obvious correlation between ICU occupancy and surveillance indicators in the first and second waves of the COVID-19 outbreak. We confirmed these observations with rigorous methods used in econometric science and in studies on surveillance systems. Third, we excluded patients attending at ED triage and requiring primary care that might lead to selection bias. However, the objective of the study was to use “real-life data” available in “real time” to predict ICU occupancy and detect aberration in syndromic surveillance. Fourth, even if P-charts and the correlation between ICU occupancy and ED surveillance data are obvious in retrospect, surveillance data could be difficult to interpret at the initial stage of a new pandemic and studies to assess the ability of ED surveillance systems to detect a

For peer review only - http://bmjopen.bmj.com/site/about/guidelines.xhtml

potential new threat need to be performed prospectively in real time. Fifth, our study lacks external validity. The results are dependent on the health care system, hospital resources, and the triage criteria used in our ED.

In conclusion, ED syndromic surveillance provides additional effective information not accessible in the usual surveillance system. The real-time availability of data makes ED syndromic surveillance a powerful tool for health care and political authorities. Future studies on the potential role of emergency services as a public health observatory are needed to further demonstrate their ability to detect and provide data on a larger scale, such as at national level or in situations of infectious diseases, but also in non-infectious diseases related to toxicological, meteorological or psychological diseases.

For peer review only - http://bmjopen.bmj.com/site/about/guidelines.xhtml

The Corresponding Author has the right to grant on behalf of all authors and does grant on behalf of all authors, a worldwide licence to the Publishers and its licensees in perpetuity, in all forms, formats and media (whether known now or created in the future), to i) publish, reproduce, distribute, display and store the Contribution, ii) translate the Contribution into other languages, create adaptations, reprints, include within collections and create summaries, extracts and/or, abstracts of the Contribution, iii) create any other derivative work(s) based on the Contribution, iv) to exploit all subsidiary rights in the Contribution, v) the inclusion of electronic links from the Contribution to third party material where-ever it may be located; and, vi) licence any third party to do any or all of the above.

Competing interests: All authors have completed the ICMJE uniform disclosure form at www.icmje.org/coi_disclosure.pdf and declare no support from any organisation for the submitted work; no financial relationships with any organisations that might have an interest in the submitted work in the previous three years; no other relationships or activities that could appear to have influenced the submitted work.

Funding: This study received no funding.

Author Contributions: FXA and PNC designed the study. DCB and FXA were responsible for the data management plan. DCB, FD and VP extracted data. FXA was responsible for data analysis. OH, PNC and FXA interpreted data and drafted the manuscript. NB, VP, DCB, FD, PE, OH, PNC and FXA contributed to the interpretation of the results, critical revision of the manuscript, and approved the final version. NB, VP, DCB, FD, PE, OH, PNC and FXA agreed to be accountable for all aspects of the work.

Ethical approval: This study was approved by the Ethics Review Board of the Canton of Vaud (CER-VD 2020-00731).

For peer review only - http://bmjopen.bmj.com/site/about/guidelines.xhtml

Data sharing: Data are available on reasonable request and with agreement from Lausanne University Hospital and Public Health Authorities of the Canton of Vaud.

Acknowledgments: The authors thank Rosemary Sudan for editorial assistance.

For peer review only

References

1 WHO. COVID-19 Public Health Emergency of International Concern (PHEIC) global research and innovation forum : towards a research roadmap. *Geneva; United Nation* 2020;:7.

2 Salathé M, Althaus CL, Neher R, *et al.* COVID-19 epidemic in Switzerland: on the importance of testing, contact tracing and isolation. *Swiss Medical Weekly* 2020;**150**. doi:10.4414/smw.2020.20225

3 Nørgaard SK, Vestergaard LS, Nielsen J, *et al.* Real-time monitoring shows substantial excess all-cause mortality during second wave of COVID-19 in Europe, October to December 2020. *Euro Surveill* 2021;**26**. doi:10.2807/1560-7917.ES.2021.26.1.2002023

4 Switzerland: WHO Coronavirus Disease (COVID-19) Dashboard. https://covid19.who.int/region/euro/country/ch (accessed 24 Mar 2021).

5 Hughes HE, Hughes TC, Morbey R, *et al.* Emergency department use during COVID-19 as described by syndromic surveillance. *Emerg Med J* 2020;**37**:600–4. doi:10.1136/emermed-2020-209980

6 Hughes HE, Edeghere O, O'Brien SJ, *et al.* Emergency department syndromic surveillance systems: a systematic review. *BMC Public Health* 2020;**20**:1891. doi:10.1186/s12889-020-09949-y

7 Haag C, Höglinger M, Moser A, *et al.* Social mixing and risk exposures for SARS-CoV-2 infections in elderly persons. *Swiss Medical Weekly* 2020;**150**. doi:10.4414/smw.2020.20416

8 Rutschmann OT, Hugli OW, Marti C, *et al.* Reliability of the revised Swiss Emergency Triage Scale: a computer simulation study. *Eur J Emerg Med* 2018;**25**:264–9. doi:10.1097/MEJ.0000000000000449

9 Subbe CP, Kruger M, Rutherford P, *et al.* Validation of a modified Early Warning Score in medical admissions. *QJM* 2001;**94**:521–6. doi:10.1093/qjmed/94.10.521

10 Smith GB, Prytherch DR, Meredith P, *et al.* The ability of the National Early Warning Score (NEWS) to discriminate patients at risk of early cardiac arrest, unanticipated intensive care unit admission, and death. *Resuscitation* 2013;**84**:465–70. doi:10.1016/j.resuscitation.2012.12.016

11 Gossin M, Walther D, Blanco J-M, *et al.* [SICOVID: a cantonal COVID information system for public health decision-making]. *Rev Med Suisse* 2020;**16**:2177–82.

12 hamilton J Douglas. *Time Series Analysis*. Princeton University Press. Princeton University Press 1994. https://press.princeton.edu/books/hardcover/9780691042893/time-series-analysis (accessed 18 Mar 2021).

13 Brown RL, Durbin J, Evans JM. Techniques for Testing the Constancy of Regression Relationships Over Time. *Journal of the Royal Statistical Society: Series B (Methodological)* 1975;**37**:149–63. doi:https://doi.org/10.1111/j.2517-6161.1975.tb01532.x

14 Rossi B, Wang Y. Vector autoregressive-based Granger causality test in the presence of instabilities. *The Stata Journal* 2019;**19**:883–99. doi:10.1177/1536867X19893631

For peer review only - http://bmjopen.bmj.com/site/about/guidelines.xhtml

15 Hutwagner L, Thompson W, Seeman GM, *et al.* The bioterrorism preparedness and response Early Aberration Reporting System (EARS). *J Urban Health* 2003;**80**:i89-96. doi:10.1007/pl00022319

16 Lawson BM, Fitzhugh EC, Hall SP, *et al.* Multifaceted Syndromic Surveillance in a Public Health Department Using the Early Aberration Reporting System. *Journal of Public Health Management and Practice* 2005;**11**:274–281.

17 Jeffery MM, D'Onofrio G, Paek H, *et al.* Trends in Emergency Department Visits and Hospital Admissions in Health Care Systems in 5 States in the First Months of the COVID-19 Pandemic in the US. *JAMA Intern Med* 2020;**180**:1328–33. doi:10.1001/jamainternmed.2020.3288

18 Hartnett KP, Kite-Powell A, DeVies J, *et al.* Impact of the COVID-19 Pandemic on Emergency Department Visits - United States, January 1, 2019-May 30, 2020. *MMWR Morb Mortal Wkly Rep* 2020;**69**:699–704. doi:10.15585/mmwr.mm6923e1

19 Casalino E, Choquet C, Bouzid D, *et al.* Analysis of Emergency Department Visits and Hospital Activity during Influenza Season, COVID-19 Epidemic, and Lockdown Periods in View of Managing a Future Disaster Risk: A Multicenter Observational Study. *Int J Environ Res Public Health* 2020;**17**. doi:10.3390/ijerph17228302

20 Boserup B, McKenney M, Elkbuli A. The impact of the COVID-19 pandemic on emergency department visits and patient safety in the United States. *Am J Emerg Med* 2020;**38**:1732–6. doi:10.1016/j.ajem.2020.06.007

21 Kuitunen I, Ponkilainen VT, Launonen AP, *et al.* The effect of national lockdown due to COVID-19 on emergency department visits. *Scandinavian Journal of Trauma, Resuscitation and Emergency Medicine* 2020;**28**:114. doi:10.1186/s13049-020-00810-0

22 Jephcote C, Hansell AL, Adams K, *et al.* Changes in air quality during COVID-19 'lockdown' in the United Kingdom. *Environmental Pollution* 2021;**272**:116011. doi:10.1016/j.envpol.2020.116011

23 Pulia MS, Hekman DJ, Glazer JM, *et al.* Electronic Health Record-Based Surveillance for Community Transmitted COVID-19 in the Emergency Department. *West J Emerg Med* 2020;**21**:748–51. doi:10.5811/westjem.2020.5.47606

24 By the COVID-19 APHP-Universities-INRIA-INSERM. Early indicators of intensive care unit bed requirement during the COVID-19 epidemic: A retrospective study in Ile-de-France region, France. *PLOS ONE* 2020;**15**:e0241406. doi:10.1371/journal.pone.0241406

25 Sanchez B, Hirzel AH, Bingisser R, *et al.* State of Emergency Medicine in Switzerland: a national profile of emergency departments in 2006. *Int J Emerg Med* 2013;**6**:23. doi:10.1186/1865-1380-6-23

26 Mesnier J, Cottin Y, Coste P, *et al.* Hospital admissions for acute myocardial infarction before and after lockdown according to regional prevalence of COVID-19 and patient profile in France: a registry study. *The Lancet Public Health* 2020;**5**:e536–42. doi:10.1016/S2468-2667(20)30188-2

Figure legends

Figure 1. Time series surveillance indicators.

For peer review only - http://bmjopen.bmj.com/site/about/guidelines.xhtml

(A) ED surveillance at Lausanne University Hospital.

(B) EMS surveillance in the Canton of Vaud.

(C) Usual surveillance in Canton of Vaud (confirmed cases and laboratory testing).

Grey areas represent ICU occupancy at Lausanne University Hospital.

Figure 2. Daily P-charts of emergency department flu-like syndrome during the second wave of the COVID-19 outbreak at Lausanne University Hospital.

Figure 3. Time series daily emergency department general activity at Lausanne University Hospital.

For peer review only

For peer review only - http://bmjopen.bmj.com/site/about/guidelines.xhtml

Table 1. Patient characteristics

	COVID period 25 Feb 20 to 25 Feb 21		Previous period 25 Feb 19 to 24 Feb 20	
	N	% (95% CI)	N	% (95% CI)
Total ED visits	37,319		42,584	
ED flu-like syndrome	2,181	5.8 (5.6-6.1)	235	0.6 (0.5-0.6)
ED isolation droplet	4,124	11.1 (10.7-11.4)	510	1.2 (1.1-1.3)
ED respiratory syndrome	3,454	9.3 (9.0-9.6)	2,713	6.4 (6.1-6.6)
ED COVID-19 confirmed	1,421	3.8 (3.6-4.0)	-	-
ED visits				
Medicine	14,558	39.0 (38.5-39.5)	15,261	35.8 (35.4-36.3)
Surgery	3,098	8.3 (8.0-8.6)	3,799	8.9 (8.7-9.2)
Resuscitation room	2,340	6.3 (6.0-6.5)	2,201	5.2 (5.0-5.4)
Ambulatory care	18,491	49.5 (49.0-50.1)	22,038	51.8 (51.3-52.2)
Age (years)				
15 -29	6,429	17.2 (16.6-17.6)	8,567	20.1 (19.7-20.5)
30 -44	7,571	20.3 (19.9-20.7)	8,929	21.0 (20.6-21.4)
45 -54	4,574	12.3 (11.9-12.6)	5,223	12.3 (12.0-12.6)
55 – 64	4,957	13.3 (12.9-13.6)	5,258	12.3 (12.0-12.7)
65 -74	4,496	12.0 (11.7-12.4)	4,826	11.3 (11.0-11.6)
≥ 75	8,987	24.1 (23.6-24.5)	9,350	22.0 (21.6-22.4)
Gender (female)	17,171	46.0 (45.5-46.5)	19,745	46.4 (45.9-46.9)
Hospitalisation	15,325	41.1 (40.6-41.6)	15,545	36.5 (36.0-37.0)
ICU	615	1.7 (1.5-1.8)	562	1.3 (1.2-1.4)
MEWS ≥5	225	0.6 (0.5-0.7)	188	0.4 (0.4-0.5)
NEWS ≥5	1,028	2.7 (2.6-2.9)	849	2.0 (1.9-2.1)
Length of stay in ED ≥ 6 hours	18,926	50.7 (50.2-51.2)	22,581	53.0 (52.6-53.5)
Death in the ED	63	0.2 (0.1-0.2)	59	0.1 (0.1-0.2)

ED: emergency department; MEWS: Modified Early Warning Score; NEWS: National Early Warning Score; ICU: intensive care unit

For peer review only - http://bmjopen.bmj.com/site/about/guidelines.xhtml

Table 2. Correlation and time lag between surveillance indicators and ICU occupancy.

	Highest correlation Coefficient 95% CI	Time Lag 1* [days]	Time Lag 2* [days]	P value for Breusch-Godfrey test and Durbin's test	P value for Granger causality	
					Lag 1	Lag 2
Confirmed cases	0.76 (0.67-0.83)	18	16	<.001	<.001	.901
Proportion of positive laboratory tests	0.92 (0.85-0.96)	15	20	<.001	<.001	.009
EMS call	0.47 (0.38-0.56)	20	7	<.001	<.001	.368
Ambulance dispatch	0.33 (0.25-0.42)	33	7	<.001	<.001	.221
ED droplet isolation	0.79 (0.71-0.86)	11	6	<.001	<.001	<.001
ED flu-like syndrome	0.73 (0.64-0.80)	13	7	<.001	<.001	<.001
ED COVID-19 confirmed	0.81 (0.73-0.88)	13	7	<.001	<.001	.020

*Lag 1 estimated by the highest correlation coefficient on correlograms and Lag 2 estimated by the lowest final prediction error (FPE) and the lowest Akaike's information criterion (AIC).

For peer review only

For peer review only - http://bmjopen.bmj.com/site/about/guidelines.xhtml

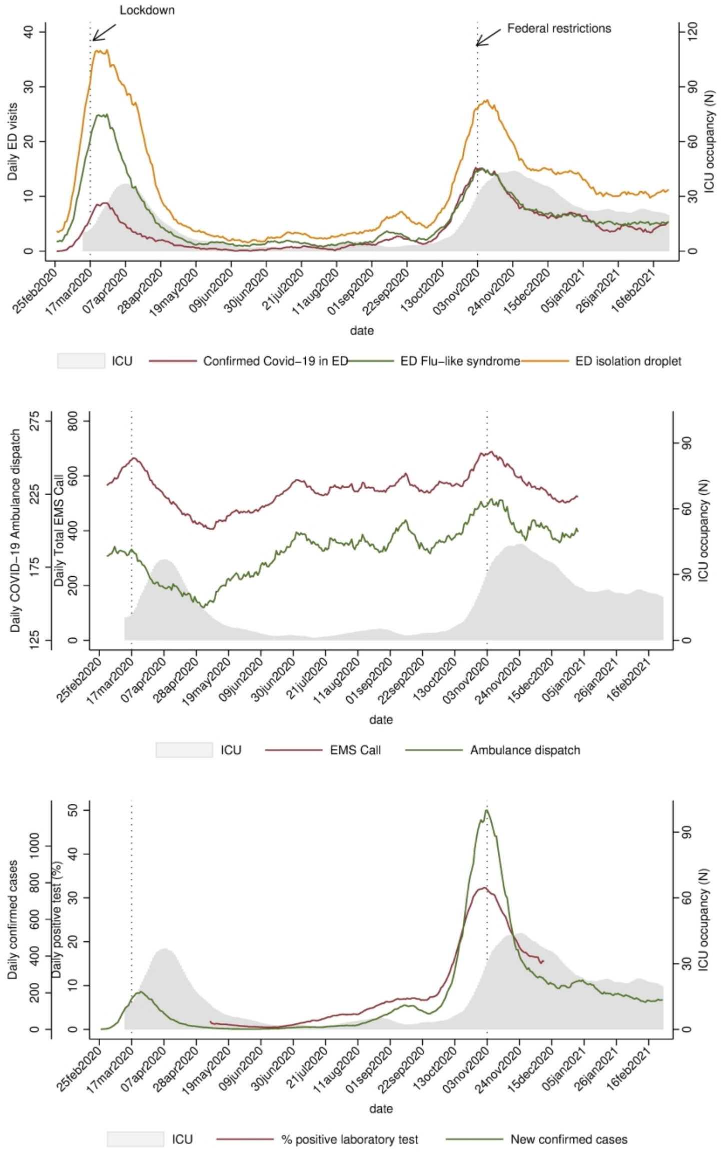

Figure 1. Time series surveillance indicators.

177x279mm (300 x 300 DPI)

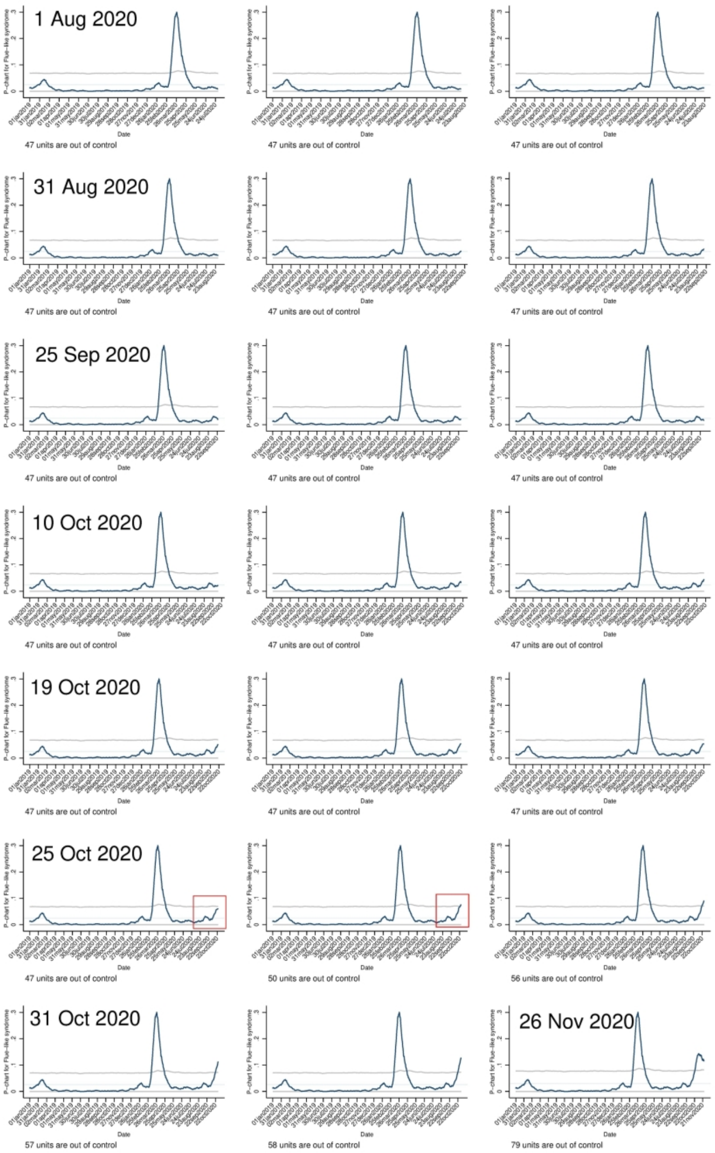

Figure 2. Daily P-charts of emergency department flu-like syndrome during the second wave of the COVID-19 outbreak at Lausanne University Hospital.

177x279mm (300 x 300 DPI)

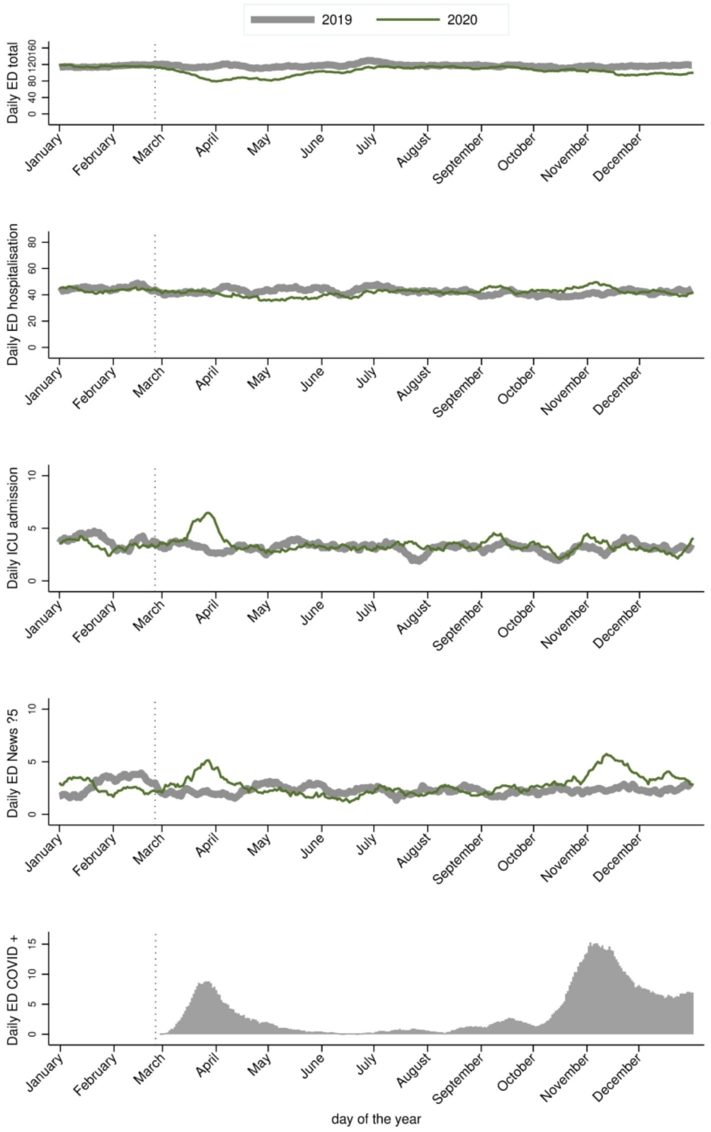

Figure 3. Time series daily emergency department general activity at Lausanne University Hospital.

177x279mm (300 x 300 DPI)

Web Appendix

eFigure 1. Correlogram between ICU occupancy and surveillance indicators at Lausanne University Hospital.

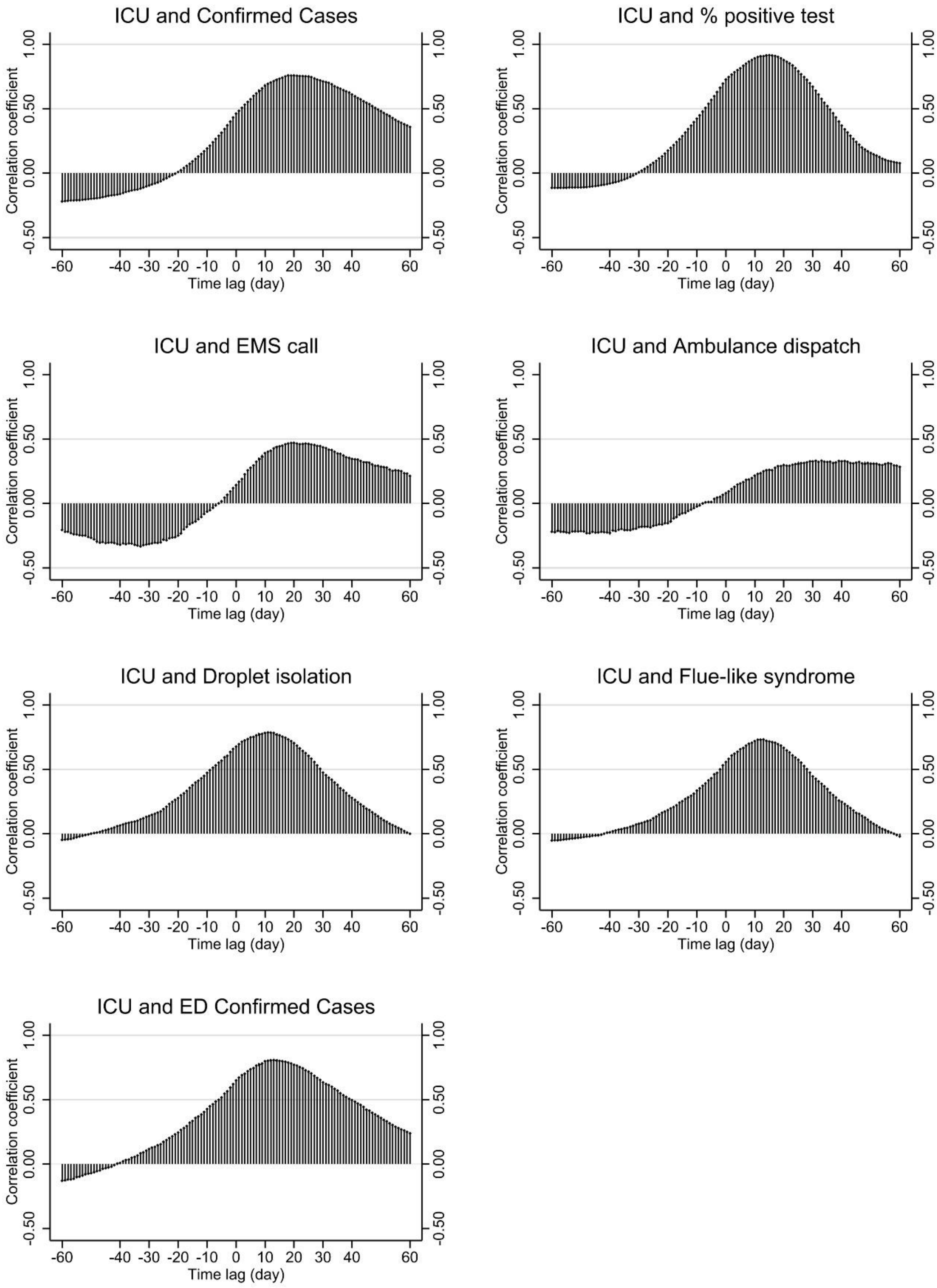

eFigure 2. Time series by daily ED specific activity at Lausanne University Hospital (trauma, cardiology and stroke).

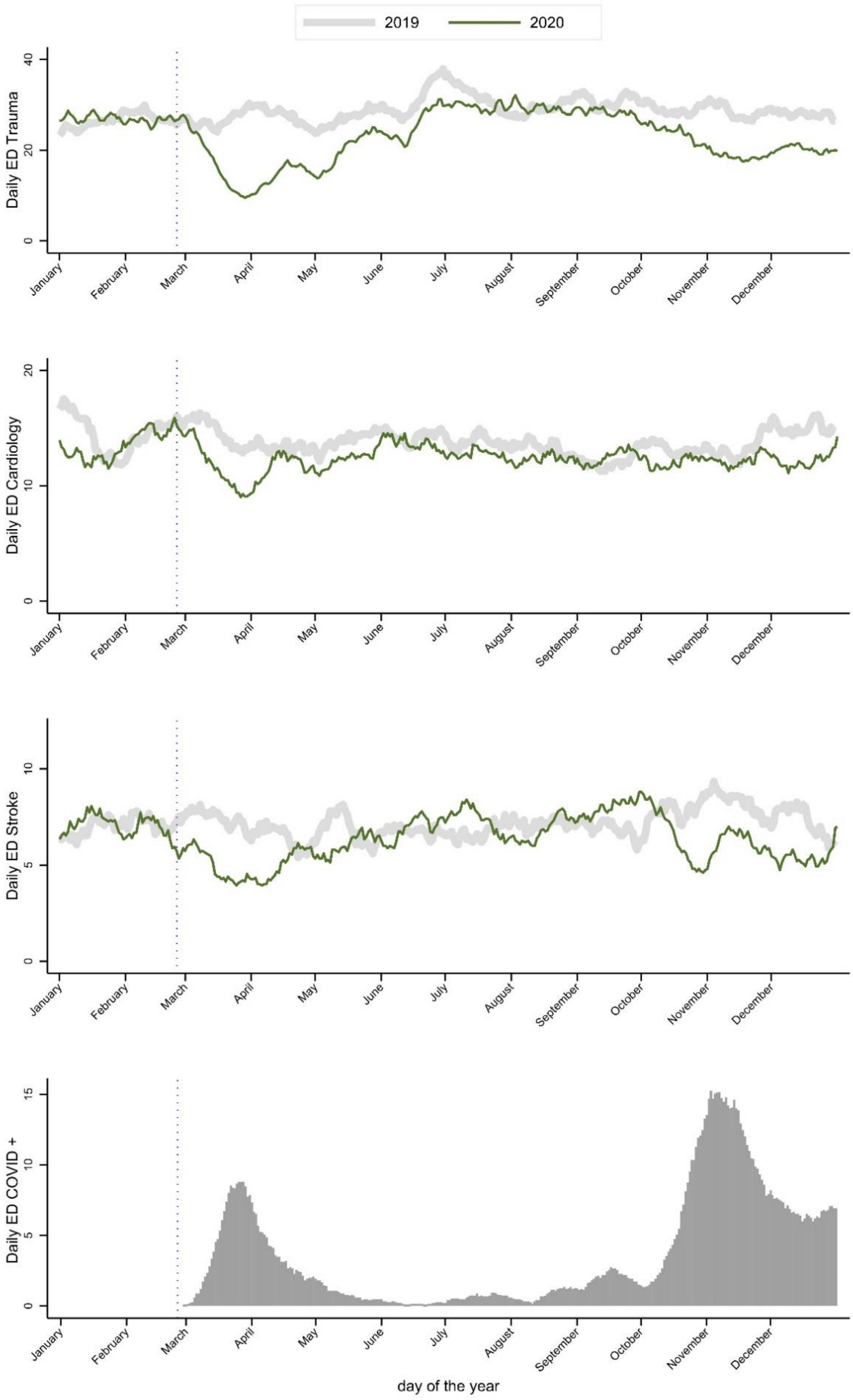

eFigure 3. Time series by daily ED specific activity at Lausanne University Hospital (GIB, diabetes, allergy).

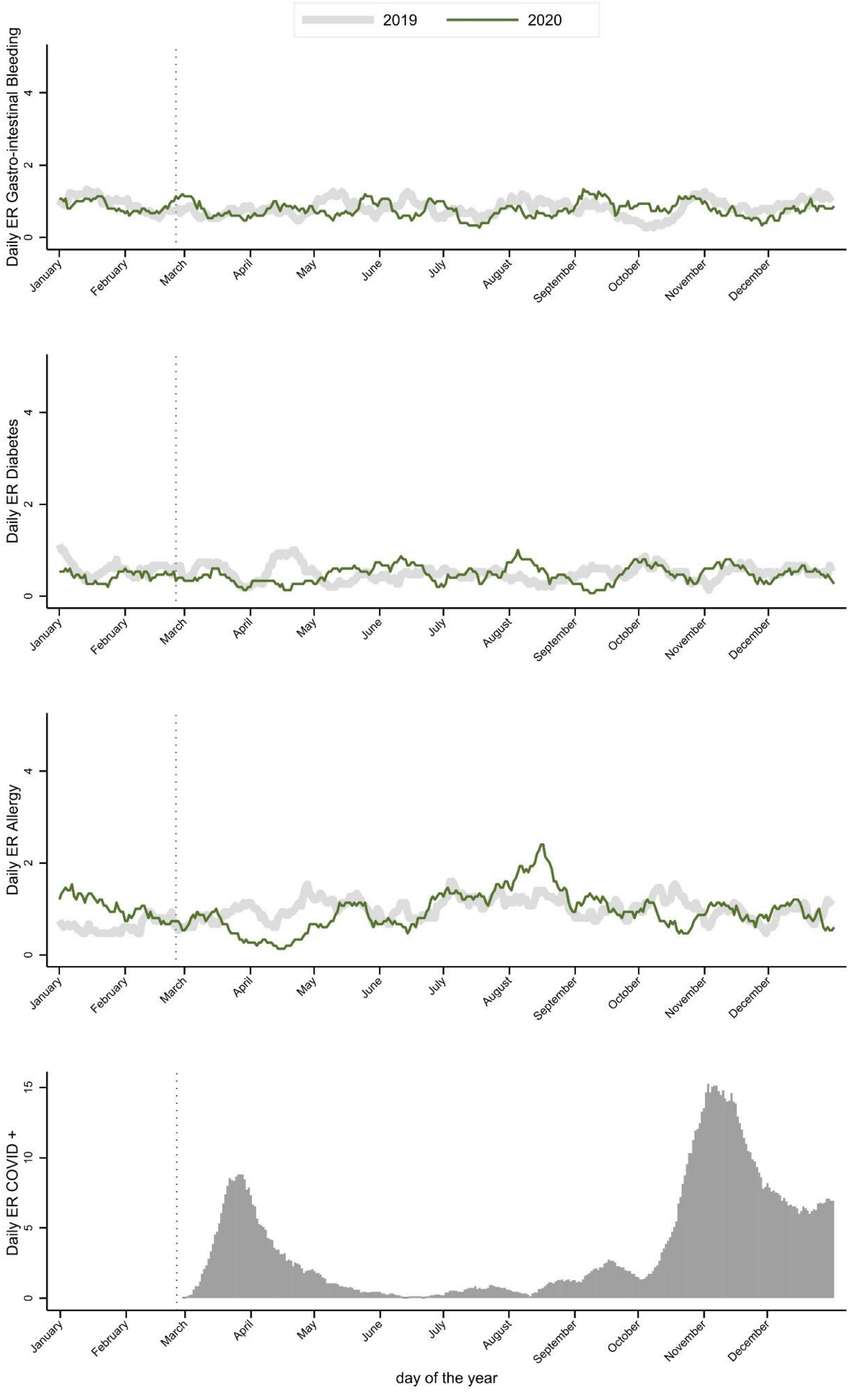

eFigure 4. Time series by ED length of stay and waiting time at Lausanne University Hospital.

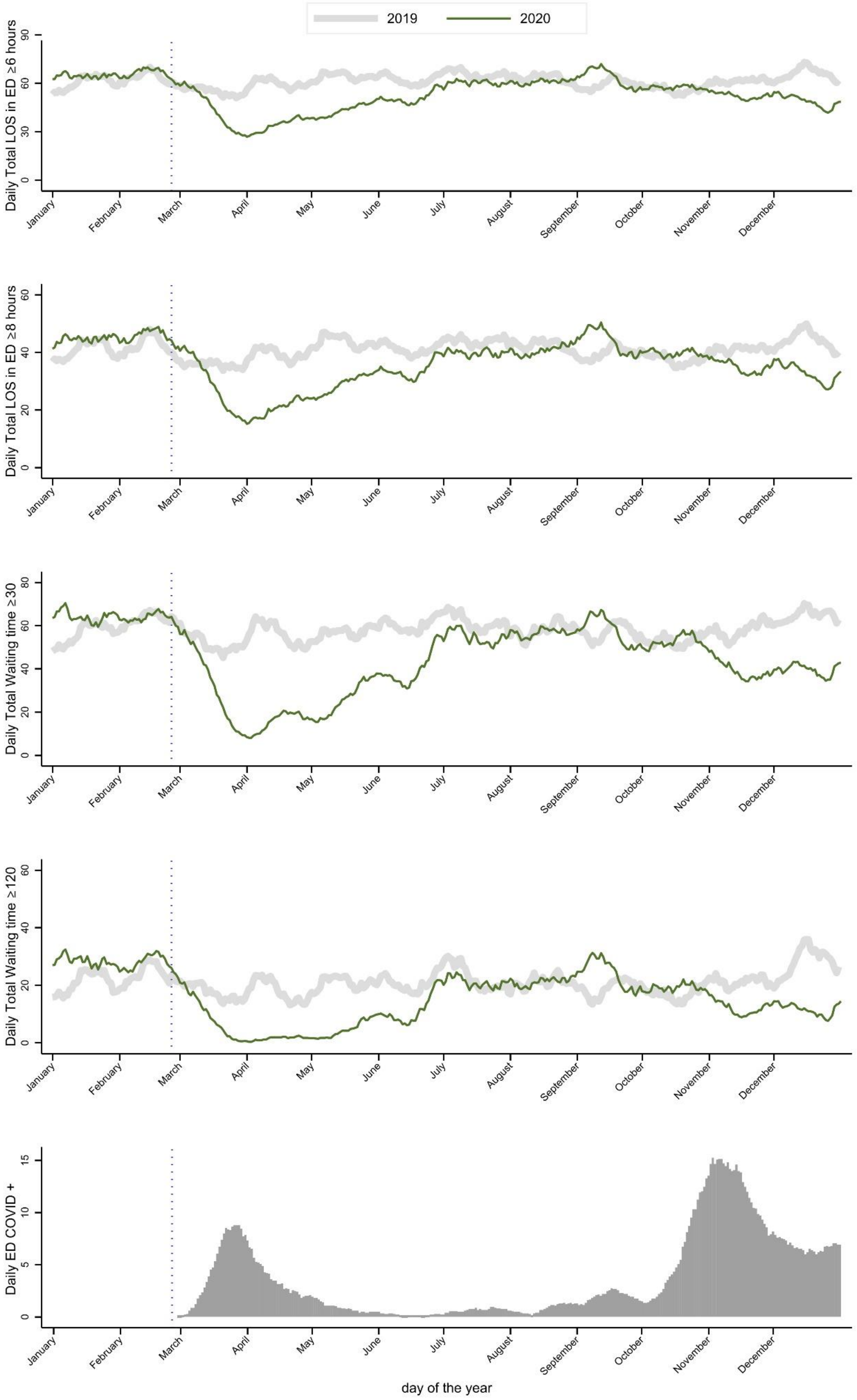

STROBE Statement—Checklist of items that should be included in reports of ***cross-sectional studies***

	Item No	Recommendation	Page No
**Title and abstract**	1	(*a*) Indicate the study's design with a commonly used term in the title or the abstract	1
		(*b*) Provide in the abstract an informative and balanced summary of what was done and what was found	2
**Introduction**			
Background/rationale	2	Explain the scientific background and rationale for the investigation being reported	4
Objectives	3	State specific objectives, including any prespecified hypotheses	4,5
**Methods**			
Study design	4	Present key elements of study design early in the paper	5
Setting	5	Describe the setting, locations, and relevant dates, including periods of recruitment, exposure, follow-up, and data collection	5
Participants	6	(*a*) Give the eligibility criteria, and the sources and methods of selection of participants	5
Variables	7	Clearly define all outcomes, exposures, predictors, potential confounders, and effect modifiers. Give diagnostic criteria, if applicable	6
Data sources/ measurement	8*	For each variable of interest, give sources of data and details of methods of assessment (measurement). Describe comparability of assessment methods if there is more than one group	6,7
Bias	9	Describe any efforts to address potential sources of bias	8
Study size	10	Explain how the study size was arrived at	NA
Quantitative variables	11	Explain how quantitative variables were handled in the analyses. If applicable, describe which groupings were chosen and why	NA
Statistical methods	12	(*a*) Describe all statistical methods, including those used to control for confounding	7,8
		(*b*) Describe any methods used to examine subgroups and interactions	7
		(*c*) Explain how missing data were addressed	8
		(*d*) If applicable, describe analytical methods taking account of sampling strategy	NA
		(*e*) Describe any sensitivity analyses	7,8
**Results**			
Participants	13*	(a) Report numbers of individuals at each stage of study—eg numbers potentially eligible, examined for eligibility, confirmed eligible, included in the study, completing follow-up, and analysed	8
		(b) Give reasons for non-participation at each stage	-
		(c) Consider use of a flow diagram	-
Descriptive data	14*	(a) Give characteristics of study participants (eg demographic, clinical, social) and information on exposures and potential confounders	8,9, table 1
		(b) Indicate number of participants with missing data for each variable of interest	Table 1
Outcome data	15*	Report numbers of outcome events or summary measures	8,9

For peer review only - http://bmjopen.bmj.com/site/about/guidelines.xhtml

For peer review only

Main results	16	(*a*) Give unadjusted estimates and, if applicable, confounder-adjusted estimates and their precision (eg, 95% confidence interval). Make clear which confounders were adjusted for and why they were included	Table 2, Fig 1, Fig 2
		(*b*) Report category boundaries when continuous variables were categorized	NA
		(*c*) If relevant, consider translating estimates of relative risk into absolute risk for a meaningful time period	NA
Other analyses	17	Report other analyses done—eg analyses of subgroups and interactions, and sensitivity analyses	Fig 3, Fig4, Web appendix
**Discussion**			
Key results	18	Summarise key results with reference to study objectives	9
Limitations	19	Discuss limitations of the study, taking into account sources of potential bias or imprecision. Discuss both direction and magnitude of any potential bias	11,12
Interpretation	20	Give a cautious overall interpretation of results considering objectives, limitations, multiplicity of analyses, results from similar studies, and other relevant evidence	10,11
Generalisability	21	Discuss the generalisability (external validity) of the study results	12
**Other information**			
Funding	22	Give the source of funding and the role of the funders for the present study and, if applicable, for the original study on which the present article is based	12

*Give information separately for exposed and unexposed groups.

Note: An Explanation and Elaboration article discusses each checklist item and gives methodological background and published examples of transparent reporting. The STROBE checklist is best used in conjunction with this article (freely available on the Web sites of PLoS Medicine at http://www.plosmedicine.org/, Annals of Internal Medicine at http://www.annals.org/, and Epidemiology at http://www.epidem.com/). Information on the STROBE Initiative is available at www.strobe-statement.org.

For peer review only

Lessons from COVID-19 syndromic surveillance through emergency department activity: a prospective time-series study from western Switzerland

Journal:	*BMJ Open*
Manuscript ID	bmjopen-2021-054504.R1
Article Type:	Original research
Date Submitted by the Author:	13-Dec-2021
Complete List of Authors:	AGERON, Francois-Xavier; Lausanne University Hospital, Emergency Department; University of Lausanne<br>Hugli, Olivier; Lausanne University Hospital, Emergency department<br>Dami, Fabrice; Lausanne University Hospital, Emergency Department<br>Caillet-Bois, David; Lausanne University Hospital, Emergency Department<br>Pittet, Valerie; University of Lausanne, Institute of Social and Preventive Medicine<br>Eckert, Philippe; Lausanne University Hospital<br>Beysard, Nicolas; Lausanne University Hospital, Emergency<br>Carron, Pierre-Nicolas; Lausanne University Hospital, Emergency
**Primary Subject Heading**:	Public health
Secondary Subject Heading:	Emergency medicine, Epidemiology, Infectious diseases, Intensive care
Keywords:	COVID-19, Public health < INFECTIOUS DISEASES, ACCIDENT & EMERGENCY MEDICINE

I, the Submitting Author has the right to grant and does grant on behalf of all authors of the Work (as defined in the below author licence), an exclusive licence and/or a non-exclusive licence for contributions from authors who are: i) UK Crown employees; ii) where BMJ has agreed a CC-BY licence shall apply, and/or iii) in accordance with the terms applicable for US Federal Government officers or employees acting as part of their official duties; on a worldwide, perpetual, irrevocable, royalty-free basis to BMJ Publishing Group Ltd ("BMJ") its licensees and where the relevant Journal is co-owned by BMJ to the co-owners of the Journal, to publish the Work in this journal and any other BMJ products and to exploit all rights, as set out in our licence.

The Submitting Author accepts and understands that any supply made under these terms is made by BMJ to the Submitting Author unless you are acting as an employee on behalf of your employer or a postgraduate student of an affiliated institution which is paying any applicable article publishing charge ("APC") for Open Access articles. Where the Submitting Author wishes to make the Work available on an Open Access basis (and intends to pay the relevant APC), the terms of reuse of such Open Access shall be governed by a Creative Commons licence – details of these licences and which Creative Commons licence will apply to this Work are set out in our licence referred to above.

Other than as permitted in any relevant BMJ Author's Self Archiving Policies, I confirm this Work has not been accepted for publication elsewhere, is not being considered for publication elsewhere and does not duplicate material already published. I confirm all authors consent to publication of this Work and authorise the granting of this licence.

For peer review only

Lessons from COVID-19 syndromic surveillance through emergency department activity: a prospective time-series study from western Switzerland

François-Xavier Ageron[1], Oliver Hugli[1], Fabrice Dami[1], David Caillet-Bois[1], Valérie Pittet[2], Philippe Eckert[3], Nicolas Beysard[1], Pierre-Nicolas Carron[1]

1 Emergency Department, Lausanne University Hospital, 46 Rue du Bugnon, 1011 Lausanne, Switzerland

2 Centre for Primary Care and Public Health, 10 route de la Corniche, University of Lausanne, Lausanne, Switzerland

3 General Directorate, Lausanne University Hospital, 21 Rue du Bugnon, 1011 Lausanne, Switzerland

For peer review only

Submitted to: *BMJ open*

Word count: 2678; 3 figures; 2 tables

Corresponding author:

Dr. Francois-Xavier AGERON, MD, PhD

Emergency Department

Lausanne University Hospital

46 Rue du Bugnon

1011 Lausanne, Switzerland

Tel.: +41 79 556 88 69

E-mail: francois-xavier.ageron@chuv.ch

For peer review only - http://bmjopen.bmj.com/site/about/guidelines.xhtml

Abstract

Objective: We aimed to assess if emergency department (ED) syndromic surveillance during the first and second waves of the COVID-19 outbreak could have improved our surveillance system.

Design, settings: We did an observational study using aggregated data from the ED of a university hospital and public health authorities in western Switzerland.

Participants: All patients admitted to the ED were included.

Primary outcome measure: The main outcome was intensive care unit (ICU) occupancy. We used time series methods for ED syndromic surveillance (flu-like syndrome, droplet isolation) and usual indicators from public health authorities (new cases, proportion of positive tests in the population).

Results: Based on 37,319 ED visits during the COVID-19 outbreak, 1421 ED visits (3.8%) were positive for severe acute respiratory syndrome coronavirus 2 (SARS-CoV-2). Patients with flu-like syndrome or droplet isolation in the ED showed a similar correlation to ICU occupancy as confirmed cases in the general population, with a time lag of approximately 13 days (0.73 [95% CI 0·64-0.80]; 0.79 [95% CI 0.71-0.86]; and 0.76 [95% CI 0.67-0.83], respectively). The proportion of positive tests in the population showed the best correlation with ICU occupancy (0.95 [95% CI 0.85-0.96]).

Conclusion: ED syndromic surveillance is an effective tool to detect and monitor a COVID-19 outbreak and to predict hospital resource needs. It would have allowed to anticipate ICU occupancy by 13 days, including significant aberration detection at the beginning of the second wave.

Keywords: emergency department, COVID-19, surveillance system, public health

For peer review only - http://bmjopen.bmj.com/site/about/guidelines.xhtml

Article summary

Strengths and limitations of this study

- A major strength of our study is that we performed and compared time series of surveillance data from the emergency department and the usual surveillance system (health regional authority and laboratory surveillance) during the COVID-19 outbreak.
- Emergency department surveillance data was correlated with intensive care unit occupancy during the outbreak.
- Rigorous methods were applied to detect a significant signal of the second wave before intensive care unit saturation, such as the Early Aberration Reporting System using a Shewhart chart.
- A limitation is that despite a good correlation and early detection of a significant signal, our study lacks external validity.
- Our study highlights that surveillance data could be difficult to interpret at the initial stage of a new pandemic and studies to assess the ability of ED surveillance systems to detect a potential new threat need to be performed in real time.

For peer review only

For peer review only - http://bmjopen.bmj.com/site/about/guidelines.xhtml

Introduction

In early 2020, the World Health Organization declared the COVID-19 outbreak to be a public health emergency of international concern.[1] Europe was particularly badly hit in spring 2020. After a relative lull in the summer, a second wave occurred in Europe in autumn 2020. Switzerland was among the most affected countries during this period with a much higher COVID-19 incidence compared to the first wave and with a 7-day incidence higher than 600 confirmed cases per 100,000.[2–4] Even if the second wave was expected, its beginning, timing and magnitude were not fully anticipated by the public health authorities.

Current public health surveillance systems include laboratory tests, death rates, hospital-based surveillance, and sentinel networks in primary care. Usual surveillance reports use the notification rate of confirmed cases and deaths, laboratory tests, hospital and intensive care unit (ICU) admission and occupancy rates. Primary care sentinel surveillance collects syndromic symptoms related to seasonal flu-like syndrome. Emergency departments (ED) are uniquely positioned at the interface of the community and hospitals and could serve as early warning systems to identify emerging threats and support decisions of public health authorities.[5] However, only one-third of European countries include ED data in their syndromic surveillance.[6] Until now, this has not been the case in Switzerland where ED data sets have not been used to detect or monitor epidemic outbreaks and, more specifically, the COVID-19 outbreak.

From July to early September 2020, most European countries observed an increase in the incidence of COVID-19 in young people <35 years, but without any significant simultaneous increase in hospital and ICU occupancy. These two concurrent numbers potentially contributed to erroneously reassure public health and political authorities. In addition, a retrospective analysis indicated a persistent higher incidence at the end of the summer in Switzerland, particularly in the western region.[7] However, it remains unknown if the monitoring of cases admitted to the ED would have provided early predictive clues on the resurgence of the pandemic. The aim of our study was to assess if ED

For peer review only - http://bmjopen.bmj.com/site/about/guidelines.xhtml

For peer review only

syndromic surveillance during the first and second waves of the COVID-19 outbreak could have improved health surveillance and provided additional information for the earlier detection of outbreak signals.

Methods

Study design and population

We did an observational study to assess whether ED syndromic surveillance would have improved the management of the first and second waves of the COVID-19 outbreak in a health system in western Switzerland, based on routine data from the Canton of Vaud and Lausanne University Hospital. The first wave occurred in March 2020 and reached a peak at the beginning of April 2020. The first lockdown in Switzerland started on 17 March 2020 and ended on 11 May 2020. The second wave occurred in November 2020. There was no lockdown applied for the second wave, but some federal restrictions were applied from 3 November 2020, such as restaurant, bar, cinema, museum and library closures.

We used aggregated data from the ED of Lausanne University Hospital, one of the five university hospitals in Switzerland, located in the French-speaking region. It serves as a primary care hospital for the Lausanne area with a population of 250,000 inhabitants and as a tertiary hospital for western Switzerland with a population of 1 million inhabitants. The ED triage includes approximatively 65,000 adult patients per year, two-thirds of whom are admitted to the ED, and one-third to the primary care consultation.

We used data from all consecutive visits leading to ED admission from 25 February, 2019 to 19 January, 2020 (pre-COVID period used as a control period) and from 25 February, 2020 (date of the first infection due to severe acute respiratory syndrome coronavirus 2 [SARS-CoV-2] in Switzerland) to 25 February, 2021. Patients referred to the primary care consultation after ED triage were

For peer review only - http://bmjopen.bmj.com/site/about/guidelines.xhtml

excluded. We also considered aggregated data for the entire population of the Canton of Vaud collected by the emergency medical service (EMS) dispatch centre and the public health authorities.

Data collection

We collected aggregated data from the ED including date and hour of admission, age group in categories, gender, main complaints at the time of admission classified using the Swiss Emergency Triage Scale (SETS),[8] deaths in the ED, hospital admissions to the ward or intensive care unit (ICU), and positive COVID-19 test notification. The modified early warning score (MEWS) and the national early warning score (NEWS) were calculated from the initial triage vital signs.[9,10] Data were extracted from the ED patient flow management software (Gyroflux®, Lausanne University Hospital, Switzerland) including triage vital signs, symptoms, isolation, ED length of stay, COVID test result, discharge diagnosis, and destination of ED patients. Only patient flow aggregated data available in real time were collected, without additional data from medical records. In addition, data were collected from the EMS dispatch centre ('Centrale d'appels sanitaires d'urgence 144') of the Canton of Vaud, including daily emergency calls and ambulance dispatch. We also collected daily hospital occupancy for COVID-19 patients in general wards and the ICU, as well as data from the Vaud health authority surveillance system (notification of new cases) and laboratory surveillance (results of PCR and antigen tests).[11]

Outcome

We selected daily absolute ICU occupancy as the primary outcome. ICU beds are a scarce resource requiring trained staff and specific medical devices. The prediction and anticipation of critical care resources has been a key issue in the COVID-19 outbreak. We considered the absolute ICU bed occupancy and not the ICU occupancy rate as the total number of ICU beds regularly evolved during the pandemic, according to needs and available resources (i.e. an increase from 35 to 76 beds).

Surveillance indicators

For peer review only - http://bmjopen.bmj.com/site/about/guidelines.xhtml

We studied and compared 'usual' and ED-specific surveillance indicators for COVID-19. Usual surveillance indicators were: 1) number of new confirmed cases of COVID-19 in the population (notification to cantonal public health authorities by medical laboratories or a general practitioner based on a PCR or antigen test); and 2) laboratory surveillance with the proportion of positive tests (PCR and antigen) from all tests performed. ED surveillance indicators were: 1) number of confirmed cases of COVID-19 during ED stay (PCR test); 2) number of patients subjected to droplet isolation measures in the ED; 3) syndromic surveillance with flu-like syndrome in the ED at triage; 4) number of EMS calls; and 5) number of ambulance dispatches.

Data analysis

We applied time series analyses for ED COVID-19 visits and for syndromic surveillance, including infectious disease, respiratory disease, cardiac symptoms including chest pain, neurologic symptoms including acute paralysis, gastrointestinal bleeding, trauma, psychiatric disorders, and hyper- or hypoglycaemia. We plotted the time series of syndromic surveillance data during the COVID-19 period and compared these to the same period of 2019. We smoothed time-series curves based on the moving 7-day average. We compared graphically ED-EMS surveillance and usual surveillance in the general population and explored the relationship between ICU occupancy and ED-EMS surveillance and traditional surveillance indicators by cross-correlation, and plotted correlograms. We tested the correlation between time series using the Breusch-Godfrey test for higher-order serial correlation and Durbin's alternative test for serial correlation.[12,13] The time lag in days between surveillance indicators and ICU occupancy was determined by estimating which lag showed the highest correlation on correlograms. We performed a Vector Auto-Regression (VAR) model and considered the optimal time lag for the lowest final prediction error and the lowest Akaike's information criterion. We performed Granger-causality with a linear regression model and a VAR model to determine which indicator was the best to predict ICU occupancy.[14] Quality control charts were then used to detect early aberration in daily data. The Early Aberration Reporting System

For peer review only - http://bmjopen.bmj.com/site/about/guidelines.xhtml

(EARS) uses different methods for temporal aberration detection, including the Shewhart chart (P-chart), moving average, and variation of the cumulative sum.[15,16] To assess the usefulness of the ED surveillance system, we assessed graphically the moving average for ED flu-like syndrome aberrations detected by P-chart during the second wave. The P-chart measures the fraction of nonconforming units in a sample. The control limits for the P-chart were estimated based on the confidence interval of the estimated fraction of the event in the time period using the normal approximation. The formula for the upper and lower limit was: $\mathrm{Pr} \pm 3\sqrt{\frac{\mathrm{Pr}\,(1-\mathrm{Pr})}{N}}$ where Pr was the estimated fraction in the time period. Detection of aberration occurs when the value is outside the 99.5% confidence interval. We detail the method in the supplement file (eSupplement 1). We did not reported any missing value for syndromic surveillance in the ED (mandatory item in the software). Data were analysed using Stata version 16.0 (StataCorp, College Station, TX, USA).

Patient and public involvement statement

Patients were not involved in the research question and in the design of the study.

Results

We collected 37,319 ED visits from 25 February 2020 to 25 February 2021 (COVID-19 period) and 42,584 ED visits from 25 February 2019 to 19 January 2020 (pre-COVID [control] period). We reported 1421 (3.8%) confirmed cases of COVID-19 during ED stay, 2181 (5.8%) flu-like syndromes, and 4124 (11.1%) ED visits with droplet isolation (table 1). An increase of flu-like syndromes was observed during the COVID-19 period. The frequency of ICU admission also increased during the COVID-19 period by 30% (OR 1.30; 95% CI 1.15-1.47; P<.001).

We plotted routine surveillance indicators (confirmed cases, laboratory surveillance and ICU occupancy) and emergency surveillance indicators (EMS and ED indicators) (figure 1). The frequency of positive laboratory tests and confirmed cases first immediately increased, followed by ED flu-like syndrome, ED isolation droplet, and confirmed ED COVID-19. All indicators followed exactly the trend

For peer review only - http://bmjopen.bmj.com/site/about/guidelines.xhtml

in ICU occupancy with a time lag, depending on the indicators. ED flu-like syndrome and ED droplet isolation showed a higher increase in the first wave than the second wave compared to ED COVID-19-confirmed cases. All surveillance indicators, except the EMS total number of calls, showed a good correlation with ICU occupancy (table 2). Correlograms showed a positive correlation for all indicators during the second wave (eSupplement 2). The highest correlations between ED-EMS surveillance indicators and ICU occupancy were obtained with time lags of 10 to 13 days (table 2). A significant aberration was detected as of 8 March 2020 for the first wave and as of 25 October 2020 for the second wave (figure 2). Aberrations were detected more than three weeks before the maximum ICU occupancy was reached. A selection of daily P-charts for ED flu-like syndrome during the second wave are presented in the supplement (eSupplement 3).

Daily ED activity is presented in figure 3. The total number of ED visits decreased during the first and second waves compared to ED activity in the previous year. Hospital admission remained stable, with a slight increase during the second wave. The number of patients who presented an intermediate-to-high risk of critical care (NEWS ≥ 5) increased during the first and second waves. Compared to 2019, trauma, cardiology, and stroke activity decreased during the first wave and to a lesser extent during the second wave (eSupplement 4). Gastrointestinal bleeding and diabetes were unchanged during both waves (eSupplement 5). Allergy decreased during the spring lockdown and increased during the summer break. ED length of stay and waiting time decreased during the first wave (eSupplement 6). During the second wave, ED length of stay decreased on a smaller scale.

Discussion

Our study shows the potential for ED syndromic surveillance as an effective tool to detect and monitor COVID-19 outbreaks and to predict hospital resource needs. The ED surveillance system correlated with ICU occupancy and would have allowed to anticipate ICU occupancy by 11 to 13 days. Of note, it would have also enabled significant aberration detection at the beginning of the second

For peer review only

For peer review only - http://bmjopen.bmj.com/site/about/guidelines.xhtml

wave. In addition, ED surveillance would provide useful information to plan hospital bed needs, including the number and severity of patients admitted to the ED, hospital and ICU admissions, and hospital resources required for trauma, cardiology and neurology patients.

Comparison with other studies

Similar to others, we found a decrease in the total number of ED visits during the first wave.[17–20] Many countries implemented a lockdown during the first wave of the outbreak that explained the decrease in ED visits to a large extent.[21] Importantly, our syndromic surveillance results allow to describe with finer granularity the change in ED activity. During lockdown, we observed a decrease of certain diseases associated with exposure to environmental factors, such as allergy or CO_2 emission. Jephcote et al also reported a change in air quality during lockdown in the United Kingdom.[22] Kuitunen et al showed that the volume of road traffic and ED visits decreased at the same time and we also observed a reduction in the number of minor and major traumatic injuries.[21] The COVID-19 outbreak well illustrated that a change in human activities contributing to pollution has an immediate effect on population health.

We showed that ED surveillance data was sufficiently accurate to detect changes in the epidemiology of the COVID-19 outbreak, based on our current system using syndromic flu-like presentations and isolation measures for droplet. In the USA, Pulia and al showed that the surveillance of patients placed in respiratory isolation for an acute respiratory infection was useful to identify and monitor trends during the pandemic.[23] In Paris (France), researchers found that ED visits and EMS calls were correlated with ICU admission, as was the proportion of positive PCR tests.[24] Similar to the Paris study, we showed that ED surveillance predicts ICU occupancy with a time lag of 13 days and the proportion of positive laboratory tests with a lag of 15 days.

Clinical implications

ED visits have constantly risen during the last decade and one-fourth to one-third of the population visit an ED annually.[25] EDs have become an important player in the public health system and an

For peer review only - http://bmjopen.bmj.com/site/about/guidelines.xhtml

For peer review only

interface between primary care and the hospital. Indeed, the ED represents today almost the only clinical pathway to unscheduled in-hospital care. For this reason, the ED has the potential to become a real-time observatory of public health if properly designed with well-defined indicators. Consequently, it is not surprising that ED surveillance would have been an effective tool to detect and monitor COVID-19 outbreak activity as it provides simple indicators for real-time monitoring that allow a rapid response from health care authorities.

Inside hospitals, ED surveillance would be useful to plan ICU or intermediate-care unit resources by predicting ICU occupancy with a significant time lag, specific to the epidemic. Additional syndromic surveillance for surgery and other medical specialities would also be helpful to reduce some activities and re-allocate resources where they are the most needed. Of note, ED surveillance would enable to detect the indirect consequences of a pandemic, such as the change in ED visits for life-threatening conditions. It is unlikely that myocardial infarction and strokes decreased during lockdown, but the decrease in chest pain and stroke symptoms observed in the ED suggests that patients avoided attending the ED as a consequence of the 'stay-at-home' campaign and fear of nosocomial COVID-19 infection.[26] This type of ED surveillance data could incite health authorities to inform the population to alert emergency services in case of chest pain and stroke symptoms, regardless of the COVID-19 outbreak.

Strengths and weaknesses

Our study has some strengths and weaknesses. First, we used a well-described cohort of consecutive ED patient visits without missing data for outcome, syndromic surveillance, and triage severity. Follow-up was complete. Second, we used simple observations to assess the obvious correlation between ICU occupancy and surveillance indicators in the first and second waves of the COVID-19 outbreak. We confirmed these observations with rigorous methods used in econometric science and in studies on surveillance systems. Third, we excluded patients attending at ED triage and requiring primary care that might lead to selection bias. However, the objective of the study was to use 'real-

For peer review only

For peer review only - http://bmjopen.bmj.com/site/about/guidelines.xhtml

life data' available in 'real time' to predict ICU occupancy and detect aberration in syndromic surveillance. Fourth, even if P-charts and the correlation between ICU occupancy and ED surveillance data are obvious in retrospect, surveillance data could be difficult to interpret at the initial stage of a new pandemic and studies to assess the ability of ED surveillance systems to detect a potential new threat need to be performed prospectively in real time. Fifth, our study lacks external validity. The results are dependent on the health care system, hospital resources, and the triage criteria used in our ED.

In conclusion, ED syndromic surveillance provides additional effective information not accessible in the usual surveillance system. The real-time availability of data makes ED syndromic surveillance a powerful tool for health care and political authorities. Future studies on the potential role of emergency services as a public health observatory are needed to further demonstrate their ability to detect and provide data on a larger scale, such as at national level or in situations of infectious diseases, but also in non-infectious diseases related to toxicological, meteorological or psychological diseases.

For peer review only

For peer review only - http://bmjopen.bmj.com/site/about/guidelines.xhtml

The Corresponding Author has the right to grant on behalf of all authors and does grant on behalf of all authors, *a worldwide licence* to the Publishers and its licensees in perpetuity, in all forms, formats and media (whether known now or created in the future), to i) publish, reproduce, distribute, display and store the Contribution, ii) translate the Contribution into other languages, create adaptations, reprints, include within collections and create summaries, extracts and/or, abstracts of the Contribution, iii) create any other derivative work(s) based on the Contribution, iv) to exploit all subsidiary rights in the Contribution, v) the inclusion of electronic links from the Contribution to third party material where-ever it may be located; and, vi) licence any third party to do any or all of the above.

Competing interests: All authors have completed the ICMJE uniform disclosure form at www.icmje.org/coi_disclosure.pdf and declare no support from any organisation for the submitted work; no financial relationships with any organisations that might have an interest in the submitted work in the previous three years; no other relationships or activities that could appear to have influenced the submitted work.

Funding: This study received no funding.

Author Contributions: FXA and PNC designed the study. DCB and FXA were responsible for the data management plan. DCB, FD and VP extracted data. FXA was responsible for data analysis. OH, PNC and FXA interpreted data and drafted the manuscript. NB, VP, DCB, FD, PE, OH, PNC and FXA contributed to the interpretation of the results, critical revision of the manuscript, and approved the final version. NB, VP, DCB, FD, PE, OH, PNC and FXA agreed to be accountable for all aspects of the work.

Ethical approval: This study was approved by the Ethics Review Board of the Canton of Vaud (CER-VD 2020-00731).

For peer review only - http://bmjopen.bmj.com/site/about/guidelines.xhtml

Data sharing: Data are available on reasonable request and with agreement from Lausanne University Hospital and Public Health Authorities of the Canton of Vaud.

Acknowledgments: The authors thank Rosemary Sudan for editorial assistance.

For peer review only

References

1 WHO. COVID-19 Public Health Emergency of International Concern (PHEIC) global research and innovation forum : towards a research roadmap. *Geneva; United Nation* 2020;:7.

2 Salathé M, Althaus CL, Neher R, *et al.* COVID-19 epidemic in Switzerland: on the importance of testing, contact tracing and isolation. *Swiss Medical Weekly* 2020;**150**. doi:10.4414/smw.2020.20225

3 Nørgaard SK, Vestergaard LS, Nielsen J, *et al.* Real-time monitoring shows substantial excess all-cause mortality during second wave of COVID-19 in Europe, October to December 2020. *Euro Surveill* 2021;**26**. doi:10.2807/1560-7917.ES.2021.26.1.2002023

4 Switzerland: WHO Coronavirus Disease (COVID-19) Dashboard. https://covid19.who.int/region/euro/country/ch (accessed 24 Mar 2021).

5 Hughes HE, Hughes TC, Morbey R, *et al.* Emergency department use during COVID-19 as described by syndromic surveillance. *Emerg Med J* 2020;**37**:600–4. doi:10.1136/emermed-2020-209980

6 Hughes HE, Edeghere O, O'Brien SJ, *et al.* Emergency department syndromic surveillance systems: a systematic review. *BMC Public Health* 2020;**20**:1891. doi:10.1186/s12889-020-09949-y

7 Haag C, Höglinger M, Moser A, *et al.* Social mixing and risk exposures for SARS-CoV-2 infections in elderly persons. *Swiss Medical Weekly* 2020;**150**. doi:10.4414/smw.2020.20416

8 Rutschmann OT, Hugli OW, Marti C, *et al.* Reliability of the revised Swiss Emergency Triage Scale: a computer simulation study. *Eur J Emerg Med* 2018;**25**:264–9. doi:10.1097/MEJ.0000000000000449

9 Subbe CP, Kruger M, Rutherford P, *et al.* Validation of a modified Early Warning Score in medical admissions. *QJM* 2001;**94**:521–6. doi:10.1093/qjmed/94.10.521

10 Smith GB, Prytherch DR, Meredith P, *et al.* The ability of the National Early Warning Score (NEWS) to discriminate patients at risk of early cardiac arrest, unanticipated intensive care unit admission, and death. *Resuscitation* 2013;**84**:465–70. doi:10.1016/j.resuscitation.2012.12.016

11 Gossin M, Walther D, Blanco J-M, *et al.* [SICOVID: a cantonal COVID information system for public health decision-making]. *Rev Med Suisse* 2020;**16**:2177–82.

12 hamilton J Douglas. *Time Series Analysis*. Princeton University Press. Princeton University Press 1994. https://press.princeton.edu/books/hardcover/9780691042893/time-series-analysis (accessed 18 Mar 2021).

13 Brown RL, Durbin J, Evans JM. Techniques for Testing the Constancy of Regression Relationships Over Time. *Journal of the Royal Statistical Society: Series B (Methodological)* 1975;**37**:149–63. doi:https://doi.org/10.1111/j.2517-6161.1975.tb01532.x

14 Rossi B, Wang Y. Vector autoregressive-based Granger causality test in the presence of instabilities. *The Stata Journal* 2019;**19**:883–99. doi:10.1177/1536867X19893631

For peer review only - http://bmjopen.bmj.com/site/about/guidelines.xhtml

15 Hutwagner L, Thompson W, Seeman GM, *et al.* The bioterrorism preparedness and response Early Aberration Reporting System (EARS). *J Urban Health* 2003;**80**:i89-96. doi:10.1007/pl00022319

16 Lawson BM, Fitzhugh EC, Hall SP, *et al.* Multifaceted Syndromic Surveillance in a Public Health Department Using the Early Aberration Reporting System. *Journal of Public Health Management and Practice* 2005;**11**:274–281.

17 Jeffery MM, D'Onofrio G, Paek H, *et al.* Trends in Emergency Department Visits and Hospital Admissions in Health Care Systems in 5 States in the First Months of the COVID-19 Pandemic in the US. *JAMA Intern Med* 2020;**180**:1328–33. doi:10.1001/jamainternmed.2020.3288

18 Hartnett KP, Kite-Powell A, DeVies J, *et al.* Impact of the COVID-19 Pandemic on Emergency Department Visits - United States, January 1, 2019-May 30, 2020. *MMWR Morb Mortal Wkly Rep* 2020;**69**:699–704. doi:10.15585/mmwr.mm6923e1

19 Casalino E, Choquet C, Bouzid D, *et al.* Analysis of Emergency Department Visits and Hospital Activity during Influenza Season, COVID-19 Epidemic, and Lockdown Periods in View of Managing a Future Disaster Risk: A Multicenter Observational Study. *Int J Environ Res Public Health* 2020;**17**. doi:10.3390/ijerph17228302

20 Boserup B, McKenney M, Elkbuli A. The impact of the COVID-19 pandemic on emergency department visits and patient safety in the United States. *Am J Emerg Med* 2020;**38**:1732–6. doi:10.1016/j.ajem.2020.06.007

21 Kuitunen I, Ponkilainen VT, Launonen AP, *et al.* The effect of national lockdown due to COVID-19 on emergency department visits. *Scandinavian Journal of Trauma, Resuscitation and Emergency Medicine* 2020;**28**:114. doi:10.1186/s13049-020-00810-0

22 Jephcote C, Hansell AL, Adams K, *et al.* Changes in air quality during COVID-19 'lockdown' in the United Kingdom. *Environmental Pollution* 2021;**272**:116011. doi:10.1016/j.envpol.2020.116011

23 Pulia MS, Hekman DJ, Glazer JM, *et al.* Electronic Health Record-Based Surveillance for Community Transmitted COVID-19 in the Emergency Department. *West J Emerg Med* 2020;**21**:748–51. doi:10.5811/westjem.2020.5.47606

24 By the COVID-19 APHP-Universities-INRIA-INSERM. Early indicators of intensive care unit bed requirement during the COVID-19 epidemic: A retrospective study in Ile-de-France region, France. *PLOS ONE* 2020;**15**:e0241406. doi:10.1371/journal.pone.0241406

25 Sanchez B, Hirzel AH, Bingisser R, *et al.* State of Emergency Medicine in Switzerland: a national profile of emergency departments in 2006. *Int J Emerg Med* 2013;**6**:23. doi:10.1186/1865-1380-6-23

26 Mesnier J, Cottin Y, Coste P, *et al.* Hospital admissions for acute myocardial infarction before and after lockdown according to regional prevalence of COVID-19 and patient profile in France: a registry study. *The Lancet Public Health* 2020;**5**:e536–42. doi:10.1016/S2468-2667(20)30188-2

For peer review only - http://bmjopen.bmj.com/site/about/guidelines.xhtml

For peer review only

Figure legends

Figure 1. Time series surveillance indicators.

Figure 2. P-charts of emergency department flu-like syndrome during the COVID-19 outbreak at Lausanne University Hospital.

Figure 3. Time series daily emergency department general activity at Lausanne University Hospital.

For peer review only

For peer review only - http://bmjopen.bmj.com/site/about/guidelines.xhtml

Table 1. Patient characteristics

	COVID period 25 Feb 20 to 25 Feb 21		Previous period 25 Feb 19 to 24 Feb 20	
	N	% (95% CI)	N	% (95% CI)
Total ED visits	37,319		42,584	
ED flu-like syndrome	2,181	5.8 (5.6-6.1)	235	0.6 (0.5-0.6)
ED isolation droplet	4,124	11.1 (10.7-11.4)	510	1.2 (1.1-1.3)
ED respiratory syndrome	3,454	9.3 (9.0-9.6)	2,713	6.4 (6.1-6.6)
ED COVID-19 confirmed	1,421	3.8 (3.6-4.0)	-	-
ED visits				
Medicine	14,558	39.0 (38.5-39.5)	15,261	35.8 (35.4-36.3)
Surgery	3,098	8.3 (8.0-8.6)	3,799	8.9 (8.7-9.2)
Resuscitation room	2,340	6.3 (6.0-6.5)	2,201	5.2 (5.0-5.4)
Ambulatory care	18,491	49.5 (49.0-50.1)	22,038	51.8 (51.3-52.2)
Age (years)				
15 -29	6,429	17.2 (16.6-17.6)	8,567	20.1 (19.7-20.5)
30 -44	7,571	20.3 (19.9-20.7)	8,929	21.0 (20.6-21.4)
45 -54	4,574	12.3 (11.9-12.6)	5,223	12.3 (12.0-12.6)
55 – 64	4,957	13.3 (12.9-13.6)	5,258	12.3 (12.0-12.7)
65 -74	4,496	12.0 (11.7-12.4)	4,826	11.3 (11.0-11.6)
≥ 75	8,987	24.1 (23.6-24.5)	9,350	22.0 (21.6-22.4)
Gender (female)	17,171	46.0 (45.5-46.5)	19,745	46.4 (45.9-46.9)
Hospitalisation	15,325	41.1 (40.6-41.6)	15,545	36.5 (36.0-37.0)
ICU	615	1.7 (1.5-1.8)	562	1.3 (1.2-1.4)
MEWS ≥5	225	0.6 (0.5-0.7)	188	0.4 (0.4-0.5)
NEWS ≥5	1,028	2.7 (2.6-2.9)	849	2.0 (1.9-2.1)
Length of stay in ED ≥ 6 hours	18,926	50.7 (50.2-51.2)	22,581	53.0 (52.6-53.5)
Death in the ED	63	0.2 (0.1-0.2)	59	0.1 (0.1-0.2)

ED: emergency department; MEWS: Modified Early Warning Score; NEWS: National Early Warning Score; ICU: intensive care unit

For peer review only

For peer review only - http://bmjopen.bmj.com/site/about/guidelines.xhtml

Table 2. Correlation and time lag between surveillance indicators and ICU occupancy.

	Highest correlation Coefficient 95% CI	Time Lag 1* [days]	Time Lag 2* [days]	P value for Breusch-Godfrey test and Durbin's test	P value for Granger causality	
					Lag 1	Lag 2
Confirmed cases	0.76 (0.67-0.83)	18	16	<.001	<.001	.901
Proportion of positive laboratory tests	0.92 (0.85-0.96)	15	20	<.001	<.001	.009
EMS call	0.47 (0.38-0.56)	20	7	<.001	<.001	.368
Ambulance dispatch	0.33 (0.25-0.42)	33	7	<.001	<.001	.221
ED droplet isolation	0.79 (0.71-0.86)	11	6	<.001	<.001	<.001
ED flu-like syndrome	0.73 (0.64-0.80)	13	7	<.001	<.001	<.001
ED COVID-19 confirmed	0.81 (0.73-0.88)	13	7	<.001	<.001	.020

*Lag 1 estimated by the highest correlation coefficient on correlograms and Lag 2 estimated by the lowest final prediction error (FPE) and the lowest Akaike's information criterion (AIC).

For peer review only

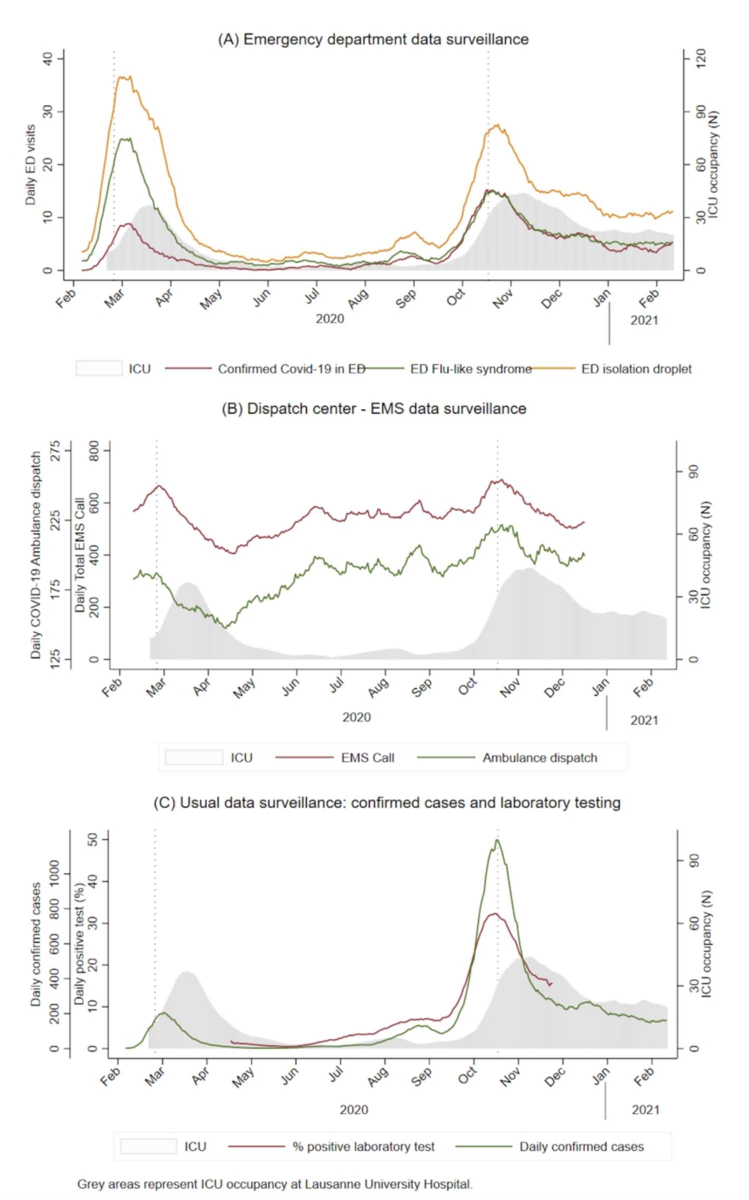

Figure 1. Time series surveillance indicators

119x192mm (300 x 300 DPI)

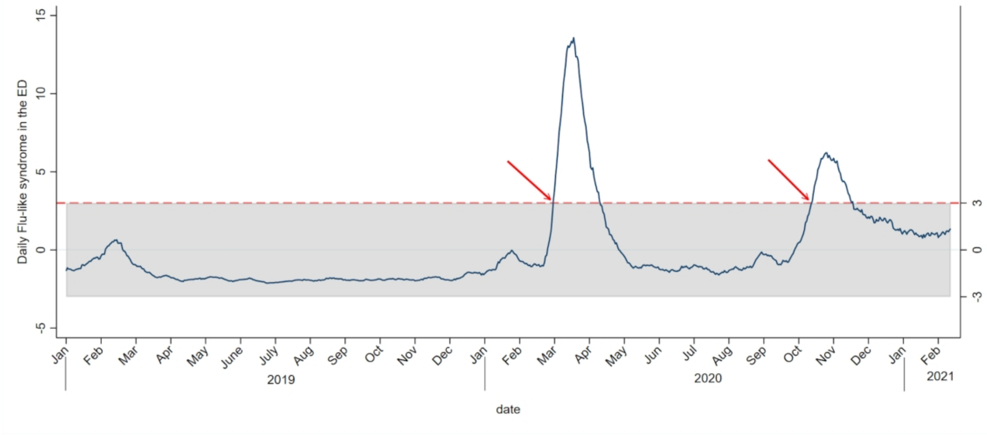

Figure 2. P-charts of emergency department flu-like syndrome during the COVID-19 outbreak at Lausanne University Hospital

202x90mm (300 x 300 DPI)

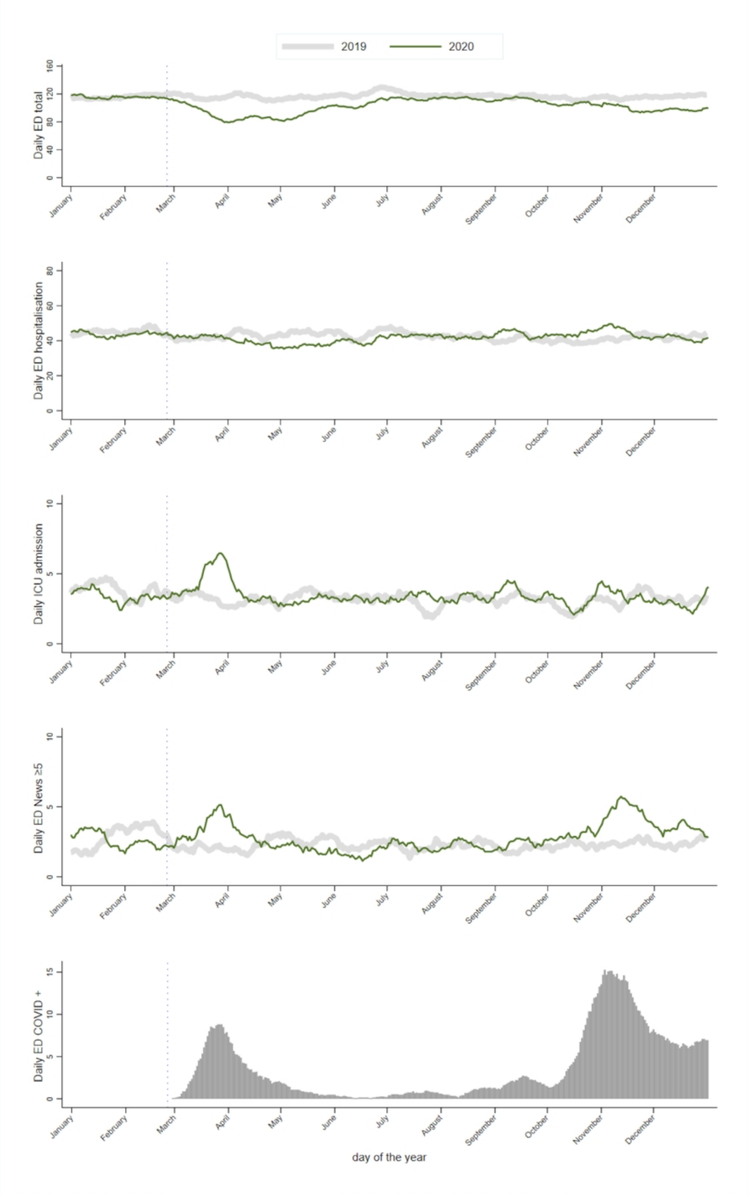

Figure 3. Time series daily emergency department general activity at Lausanne University Hospital

124x199mm (300 x 300 DPI)

Web Appendix

eSupplement 1. Method for Early Aberration Reporting System (STATA computing)

1. Identify the flu-like syndrome in emergency department software using the main complaint reported by the patient: combine the flu-like syndrome or fever/cough/shortness of breath and isolation droplet depending on the main complaint collected.

```
.gen flu_like_syndrome=1 if fever==1 | shortness_breath==1 | cough==1 & isolation_droplet
```

2. Extract the number of flu-like syndromes by day and the total number of patients attending the ED by day.

```
.collapse (sum) flu_like_syndrome total_ed id, by(date_admission)
```

3. Perform a moving average for the number of flu-like syndromes by day (7 days or less).

```
.tssmooth ma flu_like_sd_ma= flu_like_syndrome, window(7 1 7)

.tssmooth ma total_ed_ma= total_ed, window(7 1 7)
```

4. Plot the P-chart (using the usual limit $\Pr \pm 3\sqrt{\frac{\Pr(1-\Pr)}{N}}$).

```
. pchart flu_like_sd_ma date_admission total_ed_ma, sta recast(line) ytitle("Daily Flu-like syndrome in the ED",size(small)) title("P-chart moving average fraction defective", size(small)) xtitle("date",size(small)) ylabel(, labsize(small) axis(1)) ylabel(, labsize(small))
```


For peer review only - http://bmjopen.bmj.com/site/about/guidelines.xhtml

eSupplement 2. Correlogram between ICU occupancy and surveillance indicators at Lausanne University Hospital.

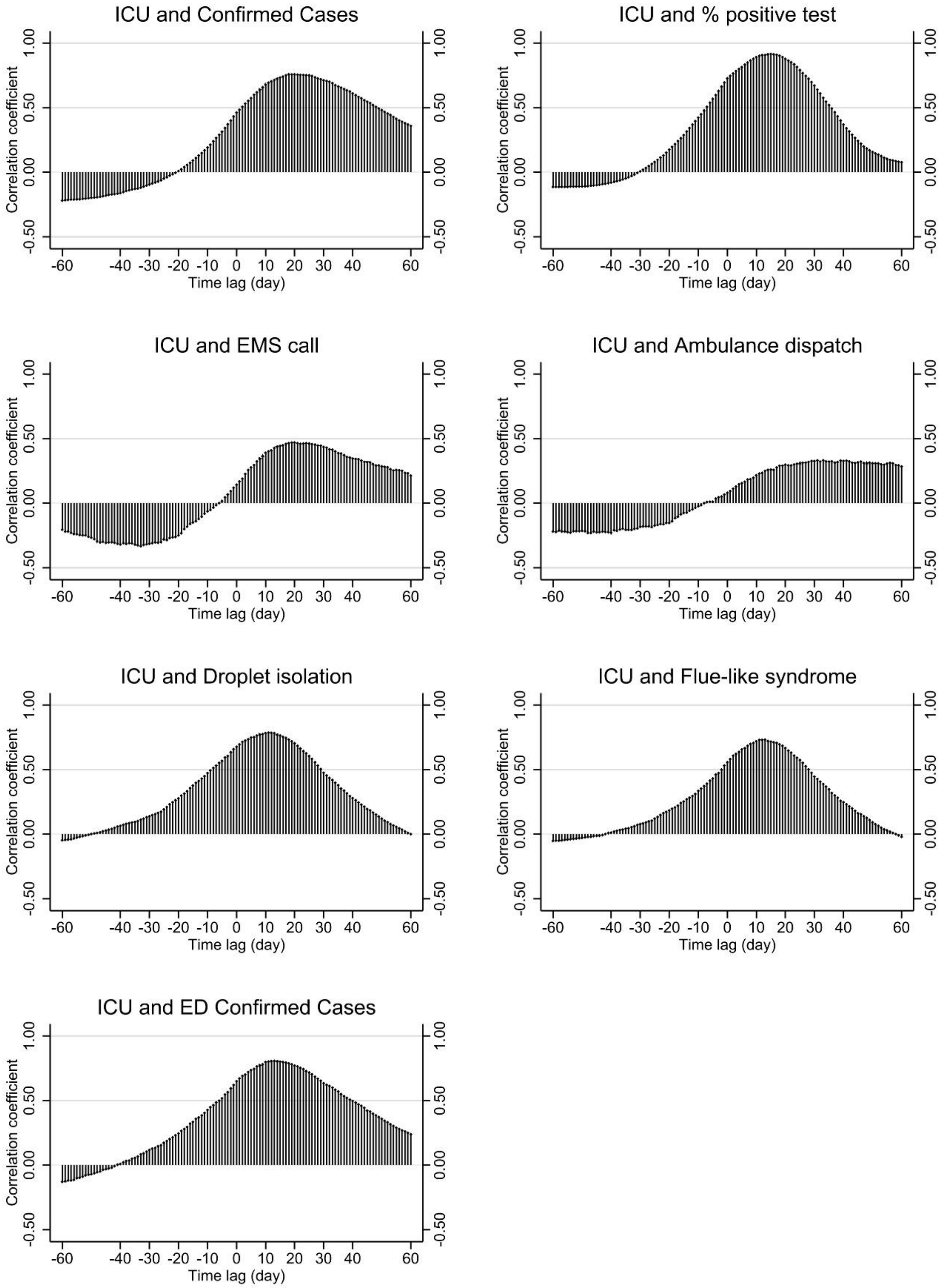

eSupplement 3. Daily P-charts of emergency department flu-like syndrome during the second wave of the COVID-19 outbreak at Lausanne University Hospital.

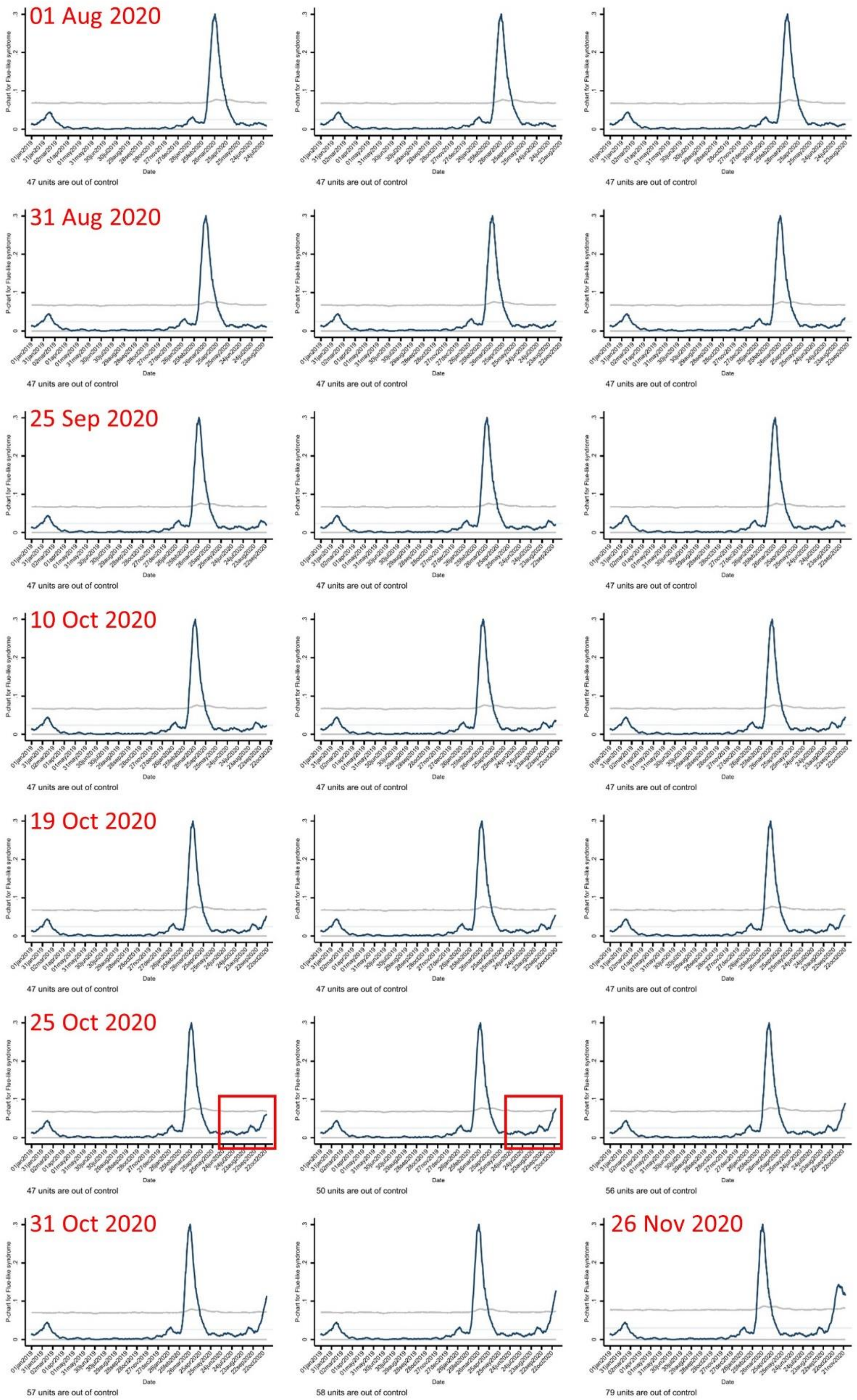

eSupplement 4. Time series by daily ED specific activity at Lausanne University Hospital (trauma, cardiology and stroke).

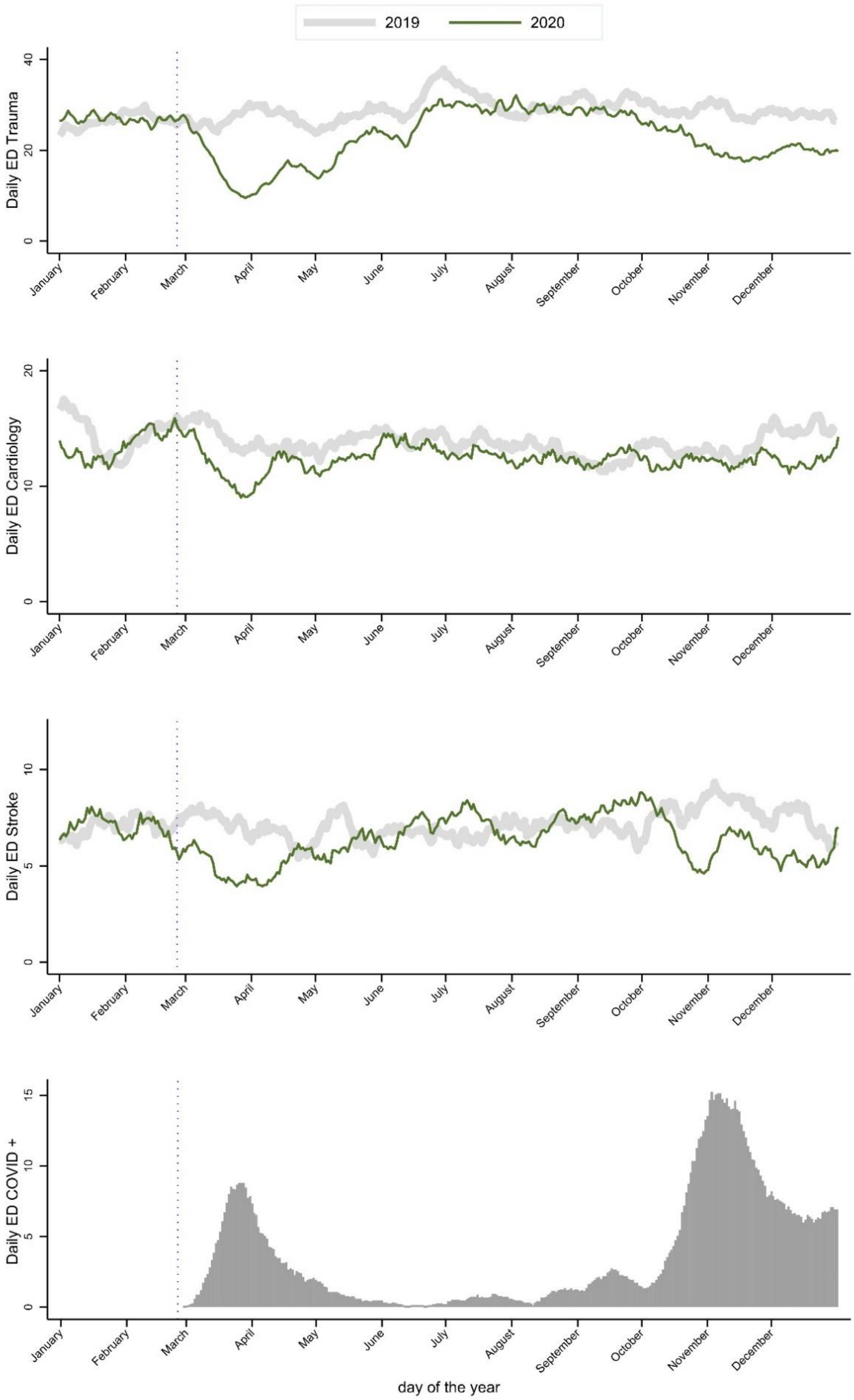

eSupplement 5. Time series by daily ED specific activity at Lausanne University Hospital (GIB, diabetes, allergy).

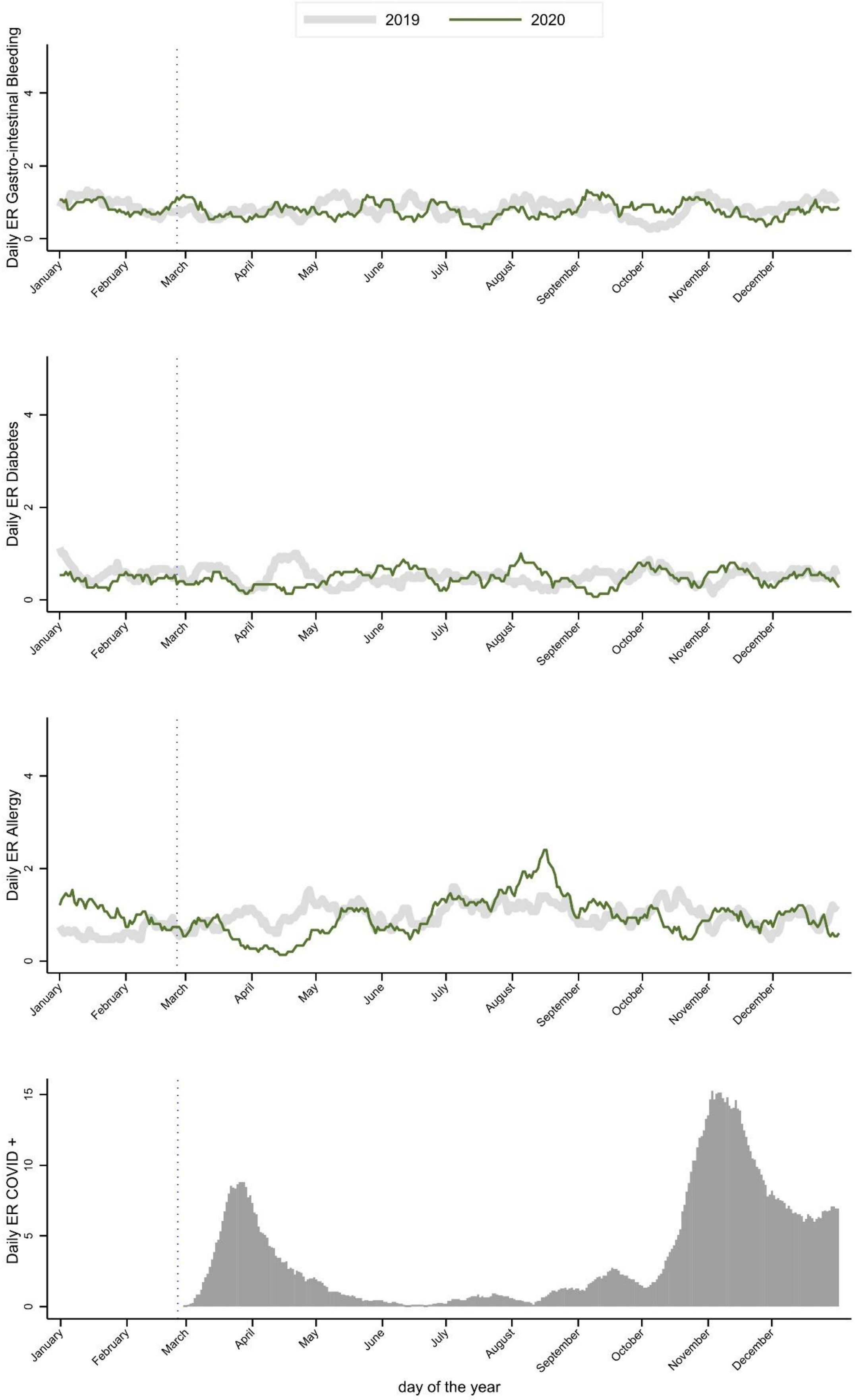

For peer review only - http://bmjopen.bmj.com/site/about/guidelines.xhtml

eSupplement 6. Time series by ED length of stay and waiting time at Lausanne University Hospital.

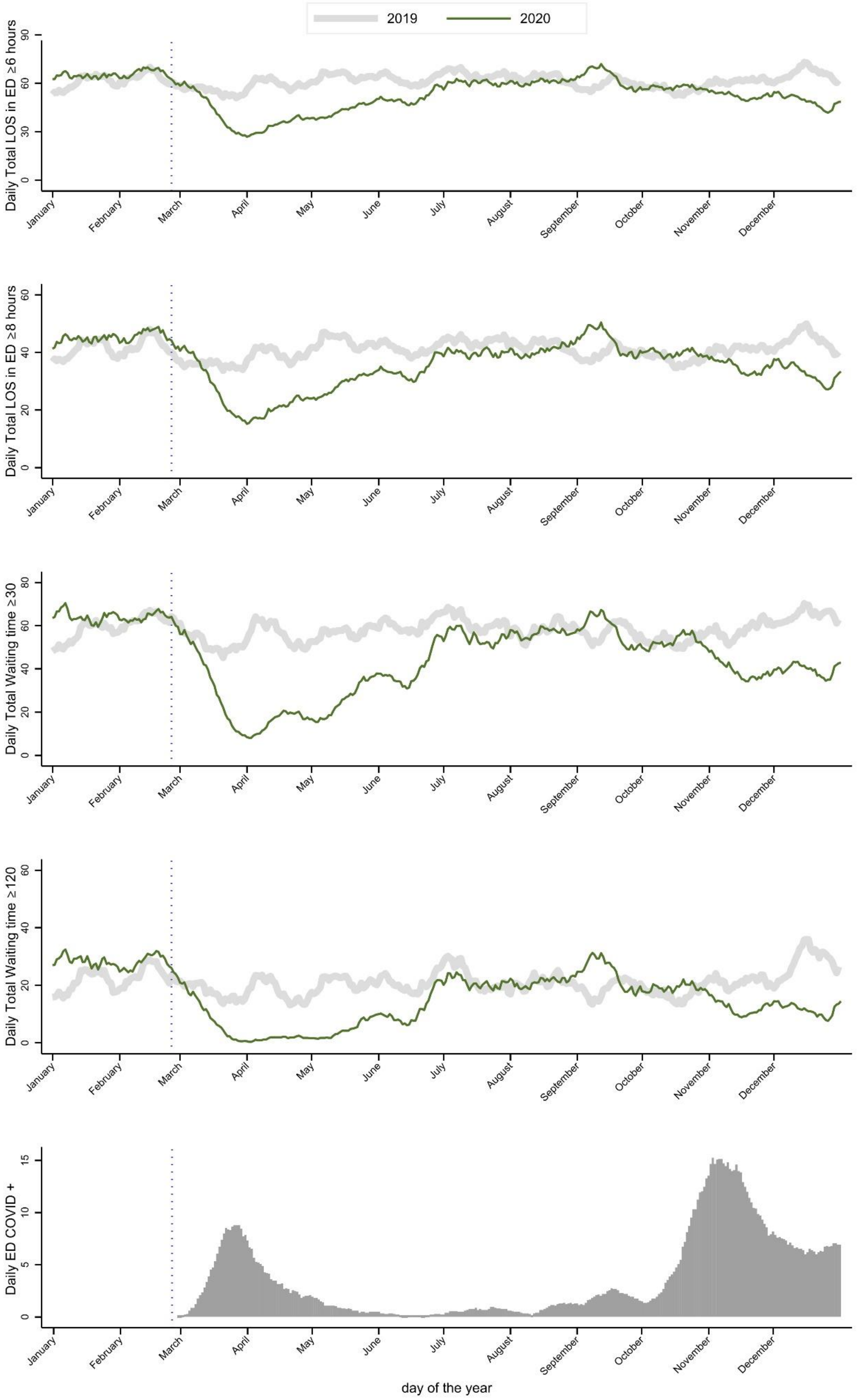

STROBE Statement—Checklist of items that should be included in reports of ***cross-sectional studies***

	Item No	Recommendation	Page No
**Title and abstract**	1	(*a*) Indicate the study's design with a commonly used term in the title or the abstract	1
		(*b*) Provide in the abstract an informative and balanced summary of what was done and what was found	2
**Introduction**			
Background/rationale	2	Explain the scientific background and rationale for the investigation being reported	4
Objectives	3	State specific objectives, including any prespecified hypotheses	4,5
**Methods**			
Study design	4	Present key elements of study design early in the paper	5
Setting	5	Describe the setting, locations, and relevant dates, including periods of recruitment, exposure, follow-up, and data collection	5
Participants	6	(*a*) Give the eligibility criteria, and the sources and methods of selection of participants	5
Variables	7	Clearly define all outcomes, exposures, predictors, potential confounders, and effect modifiers. Give diagnostic criteria, if applicable	6
Data sources/ measurement	8*	For each variable of interest, give sources of data and details of methods of assessment (measurement). Describe comparability of assessment methods if there is more than one group	6,7
Bias	9	Describe any efforts to address potential sources of bias	8
Study size	10	Explain how the study size was arrived at	NA
Quantitative variables	11	Explain how quantitative variables were handled in the analyses. If applicable, describe which groupings were chosen and why	NA
Statistical methods	12	(*a*) Describe all statistical methods, including those used to control for confounding	7,8
		(*b*) Describe any methods used to examine subgroups and interactions	7
		(*c*) Explain how missing data were addressed	8
		(*d*) If applicable, describe analytical methods taking account of sampling strategy	NA
		(*e*) Describe any sensitivity analyses	7,8
**Results**			
Participants	13*	(a) Report numbers of individuals at each stage of study—eg numbers potentially eligible, examined for eligibility, confirmed eligible, included in the study, completing follow-up, and analysed	8
		(b) Give reasons for non-participation at each stage	-
		(c) Consider use of a flow diagram	-
Descriptive data	14*	(a) Give characteristics of study participants (eg demographic, clinical, social) and information on exposures and potential confounders	8,9, table 1
		(b) Indicate number of participants with missing data for each variable of interest	Table 1
Outcome data	15*	Report numbers of outcome events or summary measures	8,9

For peer review only

Main results	16	(*a*) Give unadjusted estimates and, if applicable, confounder-adjusted estimates and their precision (eg, 95% confidence interval). Make clear which confounders were adjusted for and why they were included	Table 2, Fig 1, Fig 2
		(*b*) Report category boundaries when continuous variables were categorized	NA
		(*c*) If relevant, consider translating estimates of relative risk into absolute risk for a meaningful time period	NA
Other analyses	17	Report other analyses done—eg analyses of subgroups and interactions, and sensitivity analyses	Fig 3, Fig4, Web appendix
**Discussion**			
Key results	18	Summarise key results with reference to study objectives	9
Limitations	19	Discuss limitations of the study, taking into account sources of potential bias or imprecision. Discuss both direction and magnitude of any potential bias	11,12
Interpretation	20	Give a cautious overall interpretation of results considering objectives, limitations, multiplicity of analyses, results from similar studies, and other relevant evidence	10,11
Generalisability	21	Discuss the generalisability (external validity) of the study results	12
**Other information**			
Funding	22	Give the source of funding and the role of the funders for the present study and, if applicable, for the original study on which the present article is based	12

*Give information separately for exposed and unexposed groups.

Note: An Explanation and Elaboration article discusses each checklist item and gives methodological background and published examples of transparent reporting. The STROBE checklist is best used in conjunction with this article (freely available on the Web sites of PLoS Medicine at http://www.plosmedicine.org/, Annals of Internal Medicine at http://www.annals.org/, and Epidemiology at http://www.epidem.com/). Information on the STROBE Initiative is available at www.strobe-statement.org.

For peer review only

BMJ Open

Lessons from COVID-19 syndromic surveillance through emergency department activity: a prospective time-series study from western Switzerland

Journal:	*BMJ Open*
Manuscript ID	bmjopen-2021-054504.R2
Article Type:	Original research
Date Submitted by the Author:	21-Feb-2022
Complete List of Authors:	AGERON, Francois-Xavier; Lausanne University Hospital, Emergency Department; University of Lausanne<br>Hugli, Olivier; Lausanne University Hospital, Emergency department<br>Dami, Fabrice; Lausanne University Hospital, Emergency Department<br>Caillet-Bois, David; Lausanne University Hospital, Emergency Department<br>Pittet, Valerie; University of Lausanne, Institute of Social and Preventive Medicine<br>Eckert, Philippe; Lausanne University Hospital<br>Beysard, Nicolas; Lausanne University Hospital, Emergency<br>Carron, Pierre-Nicolas; Lausanne University Hospital, Emergency
**Primary Subject Heading**:	Public health
Secondary Subject Heading:	Emergency medicine, Epidemiology, Infectious diseases, Intensive care
Keywords:	COVID-19, Public health < INFECTIOUS DISEASES, ACCIDENT & EMERGENCY MEDICINE

SCHOLARONE™
Manuscripts

I, the Submitting Author has the right to grant and does grant on behalf of all authors of the Work (as defined in the below author licence), an exclusive licence and/or a non-exclusive licence for contributions from authors who are: i) UK Crown employees; ii) where BMJ has agreed a CC-BY licence shall apply, and/or iii) in accordance with the terms applicable for US Federal Government officers or employees acting as part of their official duties; on a worldwide, perpetual, irrevocable, royalty-free basis to BMJ Publishing Group Ltd ("BMJ") its licensees and where the relevant Journal is co-owned by BMJ to the co-owners of the Journal, to publish the Work in this journal and any other BMJ products and to exploit all rights, as set out in our licence.

The Submitting Author accepts and understands that any supply made under these terms is made by BMJ to the Submitting Author unless you are acting as an employee on behalf of your employer or a postgraduate student of an affiliated institution which is paying any applicable article publishing charge ("APC") for Open Access articles. Where the Submitting Author wishes to make the Work available on an Open Access basis (and intends to pay the relevant APC), the terms of reuse of such Open Access shall be governed by a Creative Commons licence – details of these licences and which Creative Commons licence will apply to this Work are set out in our licence referred to above.

Other than as permitted in any relevant BMJ Author's Self Archiving Policies, I confirm this Work has not been accepted for publication elsewhere, is not being considered for publication elsewhere and does not duplicate material already published. I confirm all authors consent to publication of this Work and authorise the granting of this licence.

For peer review only

Lessons from COVID-19 syndromic surveillance through emergency department activity: a prospective time-series study from western Switzerland

François-Xavier Ageron[1], Oliver Hugli[1], Fabrice Dami[1], David Caillet-Bois[1], Valérie Pittet[2], Philippe Eckert[3], Nicolas Beysard[1], Pierre-Nicolas Carron[1]

1 Emergency Department, Lausanne University Hospital, 46 Rue du Bugnon, 1011 Lausanne, Switzerland

2 Centre for Primary Care and Public Health, 10 route de la Corniche, University of Lausanne, Lausanne, Switzerland

3 General Directorate, Lausanne University Hospital, 21 Rue du Bugnon, 1011 Lausanne, Switzerland

Submitted to**:** *BMJ open*

Word count: 2678; 3 figures; 2 tables

Corresponding author:

Dr. Francois-Xavier AGERON, MD, PhD

Emergency Department

Lausanne University Hospital

46 Rue du Bugnon

1011 Lausanne, Switzerland

Tel.: +41 79 556 88 69

E-mail: francois-xavier.ageron@chuv.ch

For peer review only - http://bmjopen.bmj.com/site/about/guidelines.xhtml

Abstract

Objective: We aimed to assess if emergency department (ED) syndromic surveillance during the first and second waves of the COVID-19 outbreak could have improved our surveillance system.

Design, settings: We did an observational study using aggregated data from the ED of a university hospital and public health authorities in western Switzerland.

Participants: All patients admitted to the ED were included.

Primary outcome measure: The main outcome was intensive care unit (ICU) occupancy. We used time series methods for ED syndromic surveillance (flu-like syndrome, droplet isolation) and usual indicators from public health authorities (new cases, proportion of positive tests in the population).

Results: Based on 37,319 ED visits during the COVID-19 outbreak, 1421 ED visits (3.8%) were positive for severe acute respiratory syndrome coronavirus 2 (SARS-CoV-2). Patients with flu-like syndrome or droplet isolation in the ED showed a similar correlation to ICU occupancy as confirmed cases in the general population, with a time lag of approximately 13 days (0.73 [95% CI 0·64-0.80]; 0.79 [95% CI 0.71-0.86]; and 0.76 [95% CI 0.67-0.83], respectively). The proportion of positive tests in the population showed the best correlation with ICU occupancy (0.95 [95% CI 0.85-0.96]).

Conclusion: ED syndromic surveillance is an effective tool to detect and monitor a COVID-19 outbreak and to predict hospital resource needs. It would have allowed to anticipate ICU occupancy by 13 days, including significant aberration detection at the beginning of the second wave.

Keywords: emergency department, COVID-19, surveillance system, public health

For peer review only - http://bmjopen.bmj.com/site/about/guidelines.xhtml

Article summary

Strengths and limitations of this study

- A major strength of our study is that we performed and compared time series of surveillance data from the emergency department and the usual surveillance system (health regional authority and laboratory surveillance) during the COVID-19 outbreak.
- Emergency department surveillance data was correlated with intensive care unit occupancy during the outbreak.
- Rigorous methods were applied to detect a significant signal of the second wave before intensive care unit saturation, such as the Early Aberration Reporting System using a Shewhart chart.
- A limitation is that despite a good correlation and early detection of a significant signal, our study lacks external validity.
- Our study highlights that surveillance data could be difficult to interpret at the initial stage of a new pandemic and studies to assess the ability of ED surveillance systems to detect a potential new threat need to be performed in real time.

For peer review only

For peer review only - http://bmjopen.bmj.com/site/about/guidelines.xhtml

Introduction

In early 2020, the World Health Organization declared the COVID-19 outbreak to be a public health emergency of international concern.[1] Europe was particularly badly hit in spring 2020. After a relative lull in the summer, a second wave occurred in Europe in autumn 2020. Switzerland was among the most affected countries during this period with a much higher COVID-19 incidence compared to the first wave and with a 7-day incidence higher than 600 confirmed cases per 100,000.[2–4] Even if the second wave was expected, its beginning, timing and magnitude were not fully anticipated by the public health authorities.

Current public health surveillance systems include laboratory tests, death rates, hospital-based surveillance, and sentinel networks in primary care. Usual surveillance reports use the notification rate of confirmed cases and deaths, laboratory tests, hospital and intensive care unit (ICU) admission and occupancy rates. Primary care sentinel surveillance collects syndromic symptoms related to seasonal flu-like syndrome. Emergency departments (ED) are uniquely positioned at the interface of the community and hospitals and could serve as early warning systems to identify emerging threats and support decisions of public health authorities.[5] However, only one-third of European countries include ED data in their syndromic surveillance.[6] Until now, this has not been the case in Switzerland where ED data sets have not been used to detect or monitor epidemic outbreaks and, more specifically, the COVID-19 outbreak.

From July to early September 2020, most European countries observed an increase in the incidence of COVID-19 in young people <35 years, but without any significant simultaneous increase in hospital and ICU occupancy. These two concurrent numbers potentially contributed to erroneously reassure public health and political authorities. In addition, a retrospective analysis indicated a persistent higher incidence at the end of the summer in Switzerland, particularly in the western region.[7] However, it remains unknown if the monitoring of cases admitted to the ED would have provided early predictive clues on the resurgence of the pandemic. The aim of our study was to assess if ED

For peer review only - http://bmjopen.bmj.com/site/about/guidelines.xhtml

For peer review only

syndromic surveillance during the first and second waves of the COVID-19 outbreak could have improved health surveillance and provided additional information for the earlier detection of outbreak signals.

Methods

Study design and population

We did an observational study to assess whether ED syndromic surveillance would have improved the management of the first and second waves of the COVID-19 outbreak in a health system in western Switzerland, based on routine data from the Canton of Vaud and Lausanne University Hospital. The first wave occurred in March 2020 and reached a peak at the beginning of April 2020. The first lockdown in Switzerland started on 17 March 2020 and ended on 11 May 2020. The second wave occurred in November 2020. There was no lockdown applied for the second wave, but some federal restrictions were applied from 3 November 2020, such as restaurant, bar, cinema, museum and library closures.

We used aggregated data from the ED of Lausanne University Hospital, one of the five university hospitals in Switzerland, located in the French-speaking region. It serves as a primary care hospital for the Lausanne area with a population of 250,000 inhabitants and as a tertiary hospital for western Switzerland with a population of 1 million inhabitants. The ED triage includes approximatively 65,000 adult patients per year, two-thirds of whom are admitted to the ED, and one-third to the primary care consultation.

We used data from all consecutive visits leading to ED admission from 25 February, 2019 to 19 January, 2020 (pre-COVID period used as a control period) and from 25 February, 2020 (date of the first infection due to severe acute respiratory syndrome coronavirus 2 [SARS-CoV-2] in Switzerland) to 25 February, 2021. Patients referred to the primary care consultation after ED triage were

For peer review only - http://bmjopen.bmj.com/site/about/guidelines.xhtml

excluded. We also considered aggregated data for the entire population of the Canton of Vaud collected by the emergency medical service (EMS) dispatch centre and the public health authorities.

Data collection

We collected aggregated data from the ED including date and hour of admission, age group in categories, gender, main complaints at the time of admission classified using the Swiss Emergency Triage Scale (SETS),[8] deaths in the ED, hospital admissions to the ward or intensive care unit (ICU), and positive COVID-19 test notification. The modified early warning score (MEWS) and the national early warning score (NEWS) were calculated from the initial triage vital signs.[9,10] Data were extracted from the ED patient flow management software (Gyroflux®, Lausanne University Hospital, Switzerland) including triage vital signs, symptoms, isolation, ED length of stay, COVID test result, discharge diagnosis, and destination of ED patients. Only patient flow aggregated data available in real time were collected, without additional data from medical records. In addition, data were collected from the EMS dispatch centre ('Centrale d'appels sanitaires d'urgence 144') of the Canton of Vaud, including daily emergency calls and ambulance dispatch. We also collected daily hospital occupancy for COVID-19 patients in general wards and the ICU, as well as data from the Vaud health authority surveillance system (notification of new cases) and laboratory surveillance (results of PCR and antigen tests).[11]

Outcome

We selected daily absolute ICU occupancy as the primary outcome. ICU beds are a scarce resource requiring trained staff and specific medical devices. The prediction and anticipation of critical care resources has been a key issue in the COVID-19 outbreak. We considered the absolute ICU bed occupancy and not the ICU occupancy rate as the total number of ICU beds regularly evolved during the pandemic, according to needs and available resources (i.e. an increase from 35 to 76 beds).

Surveillance indicators

For peer review only - http://bmjopen.bmj.com/site/about/guidelines.xhtml

We studied and compared 'usual' and ED-specific surveillance indicators for COVID-19. Usual surveillance indicators were: 1) number of new confirmed cases of COVID-19 in the population (notification to cantonal public health authorities by medical laboratories or a general practitioner based on a PCR or antigen test); and 2) laboratory surveillance with the proportion of positive tests (PCR and antigen) from all tests performed. ED surveillance indicators were: 1) number of confirmed cases of COVID-19 during ED stay (PCR test); 2) number of patients subjected to droplet isolation measures in the ED; 3) syndromic surveillance with flu-like syndrome in the ED at triage; 4) number of EMS calls; and 5) number of ambulance dispatches.

Data analysis

We applied time series analyses for ED COVID-19 visits and for syndromic surveillance, including infectious disease, respiratory disease, cardiac symptoms including chest pain, neurologic symptoms including acute paralysis, gastrointestinal bleeding, trauma, psychiatric disorders, and hyper- or hypoglycaemia. We plotted the time series of syndromic surveillance data during the COVID-19 period and compared these to the same period of 2019. We smoothed time-series curves based on the moving 7-day average. We compared graphically ED-EMS surveillance and usual surveillance in the general population and explored the relationship between ICU occupancy and ED-EMS surveillance and traditional surveillance indicators by cross-correlation, and plotted correlograms. We tested the correlation between time series using the Breusch-Godfrey test for higher-order serial correlation and Durbin's alternative test for serial correlation.[12,13] The time lag in days between surveillance indicators and ICU occupancy was determined by estimating which lag showed the highest correlation on correlograms. We performed a Vector Auto-Regression (VAR) model and considered the optimal time lag for the lowest final prediction error and the lowest Akaike's information criterion. We performed Granger-causality with a linear regression model and a VAR model to determine which indicator was the best to predict ICU occupancy.[14] Quality control charts were then used to detect early aberration in daily data. The Early Aberration Reporting System

For peer review only - http://bmjopen.bmj.com/site/about/guidelines.xhtml

For peer review only

(EARS) uses different methods for temporal aberration detection, including the Shewhart chart (P-chart), moving average, and variation of the cumulative sum.[15,16] To assess the usefulness of the ED surveillance system, we assessed graphically the moving average for ED flu-like syndrome aberrations detected by P-chart during the second wave. The P-chart measures the fraction of nonconforming units in a sample. The control limits for the P-chart were estimated based on the confidence interval of the estimated fraction of the event in the time period using the normal approximation. The formula for the upper and lower limit was: $\mathrm{Pr} \pm 3\sqrt{\frac{\mathrm{Pr}\,(1-\mathrm{Pr})}{N}}$ where Pr was the estimated fraction in the time period. Detection of aberration occurs when the value is outside the 99.5% confidence interval. We detail the method in the supplement file (eSupplement 1). We did not reported any missing value for syndromic surveillance in the ED (mandatory item in the software). The sample size was fixed during the study period. We estimated that the minimal sample size was 2,668 participants to have a 90% chance of detecting, as significant level of 5%, a difference in the correlation coefficient from 0.75 to 0.80. Data were analysed using Stata version 16.0 (StataCorp, College Station, TX, USA).

Patient and public involvement statement

Patients were not involved in the research question and in the design of the study.

Results

We collected 37,319 ED visits from 25 February 2020 to 25 February 2021 (COVID-19 period) and 42,584 ED visits from 25 February 2019 to 19 January 2020 (pre-COVID [control] period). We reported 1421 (3.8%) confirmed cases of COVID-19 during ED stay, 2181 (5.8%) flu-like syndromes, and 4124 (11.1%) ED visits with droplet isolation (table 1). An increase of flu-like syndromes was observed during the COVID-19 period. The frequency of ICU admission also increased during the COVID-19 period by 30% (OR 1.30; 95% CI 1.15-1.47; P<.001).

For peer review only - http://bmjopen.bmj.com/site/about/guidelines.xhtml

We plotted routine surveillance indicators (confirmed cases, laboratory surveillance and ICU occupancy) and emergency surveillance indicators (EMS and ED indicators) (figure 1). The frequency of positive laboratory tests and confirmed cases first immediately increased, followed by ED flu-like syndrome, ED isolation droplet, and confirmed ED COVID-19. All indicators followed exactly the trend in ICU occupancy with a time lag, depending on the indicators. ED flu-like syndrome and ED droplet isolation showed a higher increase in the first wave than the second wave compared to ED COVID-19-confirmed cases. All surveillance indicators, except the EMS total number of calls, showed a good correlation with ICU occupancy (table 2). Correlograms showed a positive correlation for all indicators during the second wave (eSupplement 2). The highest correlations between ED-EMS surveillance indicators and ICU occupancy were obtained with time lags of 10 to 13 days (table 2). A significant aberration was detected as of 8 March 2020 for the first wave and as of 25 October 2020 for the second wave (figure 2). Aberrations were detected more than three weeks before the maximum ICU occupancy was reached. A selection of daily P-charts for ED flu-like syndrome during the second wave are presented in the supplement (eSupplement 3).

Daily ED activity is presented in figure 3. The total number of ED visits decreased during the first and second waves compared to ED activity in the previous year. Hospital admission remained stable, with a slight increase during the second wave. The number of patients who presented an intermediate-to-high risk of critical care (NEWS ≥ 5) increased during the first and second waves. Compared to 2019, trauma, cardiology, and stroke activity decreased during the first wave and to a lesser extent during the second wave (eSupplement 4). Gastrointestinal bleeding and diabetes were unchanged during both waves (eSupplement 5). Allergy decreased during the spring lockdown and increased during the summer break. ED length of stay and waiting time decreased during the first wave (eSupplement 6). During the second wave, ED length of stay decreased on a smaller scale.

Discussion

For peer review only - http://bmjopen.bmj.com/site/about/guidelines.xhtml

Our study shows the potential for ED syndromic surveillance as an effective tool to detect and monitor COVID-19 outbreaks and to predict hospital resource needs. The ED surveillance system correlated with ICU occupancy and would have allowed to anticipate ICU occupancy by 11 to 13 days. Of note, it would have also enabled significant aberration detection at the beginning of the second wave. In addition, ED surveillance would provide useful information to plan hospital bed needs, including the number and severity of patients admitted to the ED, hospital and ICU admissions, and hospital resources required for trauma, cardiology and neurology patients.

Comparison with other studies

Similar to others, we found a decrease in the total number of ED visits during the first wave.[17–20] Many countries implemented a lockdown during the first wave of the outbreak that explained the decrease in ED visits to a large extent.[21] Importantly, our syndromic surveillance results allow to describe with finer granularity the change in ED activity. During lockdown, we observed a decrease of certain diseases associated with exposure to environmental factors, such as allergy or CO_2 emission. Jephcote et al also reported a change in air quality during lockdown in the United Kingdom.[22] Kuitunen et al showed that the volume of road traffic and ED visits decreased at the same time and we also observed a reduction in the number of minor and major traumatic injuries.[21] The COVID-19 outbreak well illustrated that a change in human activities contributing to pollution has an immediate effect on population health.

We showed that ED surveillance data was sufficiently accurate to detect changes in the epidemiology of the COVID-19 outbreak, based on our current system using syndromic flu-like presentations and isolation measures for droplet. In the USA, Pulia and al showed that the surveillance of patients placed in respiratory isolation for an acute respiratory infection was useful to identify and monitor trends during the pandemic.[23] In Paris (France), researchers found that ED visits and EMS calls were correlated with ICU admission, as was the proportion of positive PCR tests.[24] Similar to the

For peer review only - http://bmjopen.bmj.com/site/about/guidelines.xhtml

Paris study, we showed that ED surveillance predicts ICU occupancy with a time lag of 13 days and the proportion of positive laboratory tests with a lag of 15 days.

Clinical implications

ED visits have constantly risen during the last decade and one-fourth to one-third of the population visit an ED annually.[25] EDs have become an important player in the public health system and an interface between primary care and the hospital. Indeed, the ED represents today almost the only clinical pathway to unscheduled in-hospital care. For this reason, the ED has the potential to become a real-time observatory of public health if properly designed with well-defined indicators. Consequently, it is not surprising that ED surveillance would have been an effective tool to detect and monitor COVID-19 outbreak activity as it provides simple indicators for real-time monitoring that allow a rapid response from health care authorities.

Inside hospitals, ED surveillance would be useful to plan ICU or intermediate-care unit resources by predicting ICU occupancy with a significant time lag, specific to the epidemic. Additional syndromic surveillance for surgery and other medical specialities would also be helpful to reduce some activities and re-allocate resources where they are the most needed. Of note, ED surveillance would enable to detect the indirect consequences of a pandemic, such as the change in ED visits for life-threatening conditions. It is unlikely that myocardial infarction and strokes decreased during lockdown, but the decrease in chest pain and stroke symptoms observed in the ED suggests that patients avoided attending the ED as a consequence of the 'stay-at-home' campaign and fear of nosocomial COVID-19 infection.[26] This type of ED surveillance data could incite health authorities to inform the population to alert emergency services in case of chest pain and stroke symptoms, regardless of the COVID-19 outbreak.

Strengths and weaknesses

Our study has some strengths and weaknesses. First, we used a well-described cohort of consecutive ED patient visits without missing data for outcome, syndromic surveillance, and triage severity.

For peer review only - http://bmjopen.bmj.com/site/about/guidelines.xhtml

Follow-up was complete. Second, we used simple observations to assess the obvious correlation between ICU occupancy and surveillance indicators in the first and second waves of the COVID-19 outbreak. We confirmed these observations with rigorous methods used in econometric science and in studies on surveillance systems. Third, we excluded patients attending at ED triage and requiring primary care that might lead to selection bias. However, the objective of the study was to use 'real-life data' available in 'real time' to predict ICU occupancy and detect aberration in syndromic surveillance. Fourth, even if P-charts and the correlation between ICU occupancy and ED surveillance data are obvious in retrospect, surveillance data could be difficult to interpret at the initial stage of a new pandemic and studies to assess the ability of ED surveillance systems to detect a potential new threat need to be performed prospectively in real time. Fifth, our study lacks external validity. The results are dependent on the health care system, hospital resources, and the triage criteria used in our ED.

In conclusion, ED syndromic surveillance provides additional effective information not accessible in the usual surveillance system. The real-time availability of data makes ED syndromic surveillance a powerful tool for health care and political authorities. Future studies on the potential role of emergency services as a public health observatory are needed to further demonstrate their ability to detect and provide data on a larger scale, such as at national level or in situations of infectious diseases, but also in non-infectious diseases related to toxicological, meteorological or psychological diseases.

For peer review only - http://bmjopen.bmj.com/site/about/guidelines.xhtml

The Corresponding Author has the right to grant on behalf of all authors and does grant on behalf of all authors, *a worldwide licence* to the Publishers and its licensees in perpetuity, in all forms, formats and media (whether known now or created in the future), to i) publish, reproduce, distribute, display and store the Contribution, ii) translate the Contribution into other languages, create adaptations, reprints, include within collections and create summaries, extracts and/or, abstracts of the Contribution, iii) create any other derivative work(s) based on the Contribution, iv) to exploit all subsidiary rights in the Contribution, v) the inclusion of electronic links from the Contribution to third party material where-ever it may be located; and, vi) licence any third party to do any or all of the above.

Competing interests: All authors have completed the ICMJE uniform disclosure form at www.icmje.org/coi_disclosure.pdf and declare no support from any organisation for the submitted work; no financial relationships with any organisations that might have an interest in the submitted work in the previous three years; no other relationships or activities that could appear to have influenced the submitted work.

Funding: This study received no funding.

Author Contributions: FXA and PNC designed the study. DCB and FXA were responsible for the data management plan. DCB, FD and VP extracted data. FXA was responsible for data analysis. OH, PNC and FXA interpreted data and drafted the manuscript. NB, VP, DCB, FD, PE, OH, PNC and FXA contributed to the interpretation of the results, critical revision of the manuscript, and approved the final version. NB, VP, DCB, FD, PE, OH, PNC and FXA agreed to be accountable for all aspects of the work.

Ethical approval: This study was approved by the Ethics Review Board of the Canton of Vaud (CER-VD 2020-00731).

For peer review only - http://bmjopen.bmj.com/site/about/guidelines.xhtml

Data sharing: Data are available on reasonable request and with agreement from Lausanne University Hospital and Public Health Authorities of the Canton of Vaud.

Acknowledgments: The authors thank Rosemary Sudan for editorial assistance.

For peer review only

References

1 WHO. COVID-19 Public Health Emergency of International Concern (PHEIC) global research and innovation forum : towards a research roadmap. *Geneva; United Nation* 2020;:7.

2 Salathé M, Althaus CL, Neher R, *et al.* COVID-19 epidemic in Switzerland: on the importance of testing, contact tracing and isolation. *Swiss Medical Weekly* 2020;**150**. doi:10.4414/smw.2020.20225

3 Nørgaard SK, Vestergaard LS, Nielsen J, *et al.* Real-time monitoring shows substantial excess all-cause mortality during second wave of COVID-19 in Europe, October to December 2020. *Euro Surveill* 2021;**26**. doi:10.2807/1560-7917.ES.2021.26.1.2002023

4 Switzerland: WHO Coronavirus Disease (COVID-19) Dashboard. https://covid19.who.int/region/euro/country/ch (accessed 24 Mar 2021).

5 Hughes HE, Hughes TC, Morbey R, *et al.* Emergency department use during COVID-19 as described by syndromic surveillance. *Emerg Med J* 2020;**37**:600–4. doi:10.1136/emermed-2020-209980

6 Hughes HE, Edeghere O, O'Brien SJ, *et al.* Emergency department syndromic surveillance systems: a systematic review. *BMC Public Health* 2020;**20**:1891. doi:10.1186/s12889-020-09949-y

7 Haag C, Höglinger M, Moser A, *et al.* Social mixing and risk exposures for SARS-CoV-2 infections in elderly persons. *Swiss Medical Weekly* 2020;**150**. doi:10.4414/smw.2020.20416

8 Rutschmann OT, Hugli OW, Marti C, *et al.* Reliability of the revised Swiss Emergency Triage Scale: a computer simulation study. *Eur J Emerg Med* 2018;**25**:264–9. doi:10.1097/MEJ.0000000000000449

9 Subbe CP, Kruger M, Rutherford P, *et al.* Validation of a modified Early Warning Score in medical admissions. *QJM* 2001;**94**:521–6. doi:10.1093/qjmed/94.10.521

10 Smith GB, Prytherch DR, Meredith P, *et al.* The ability of the National Early Warning Score (NEWS) to discriminate patients at risk of early cardiac arrest, unanticipated intensive care unit admission, and death. *Resuscitation* 2013;**84**:465–70. doi:10.1016/j.resuscitation.2012.12.016

11 Gossin M, Walther D, Blanco J-M, *et al.* [SICOVID: a cantonal COVID information system for public health decision-making]. *Rev Med Suisse* 2020;**16**:2177–82.

12 hamilton J Douglas. *Time Series Analysis*. Princeton University Press. Princeton University Press 1994. https://press.princeton.edu/books/hardcover/9780691042893/time-series-analysis (accessed 18 Mar 2021).

13 Brown RL, Durbin J, Evans JM. Techniques for Testing the Constancy of Regression Relationships Over Time. *Journal of the Royal Statistical Society: Series B (Methodological)* 1975;**37**:149–63. doi:https://doi.org/10.1111/j.2517-6161.1975.tb01532.x

14 Rossi B, Wang Y. Vector autoregressive-based Granger causality test in the presence of instabilities. *The Stata Journal* 2019;**19**:883–99. doi:10.1177/1536867X19893631

For peer review only - http://bmjopen.bmj.com/site/about/guidelines.xhtml

15 Hutwagner L, Thompson W, Seeman GM, *et al.* The bioterrorism preparedness and response Early Aberration Reporting System (EARS). *J Urban Health* 2003;**80**:i89-96. doi:10.1007/pl00022319

16 Lawson BM, Fitzhugh EC, Hall SP, *et al.* Multifaceted Syndromic Surveillance in a Public Health Department Using the Early Aberration Reporting System. *Journal of Public Health Management and Practice* 2005;**11**:274–281.

17 Jeffery MM, D'Onofrio G, Paek H, *et al.* Trends in Emergency Department Visits and Hospital Admissions in Health Care Systems in 5 States in the First Months of the COVID-19 Pandemic in the US. *JAMA Intern Med* 2020;**180**:1328–33. doi:10.1001/jamainternmed.2020.3288

18 Hartnett KP, Kite-Powell A, DeVies J, *et al.* Impact of the COVID-19 Pandemic on Emergency Department Visits - United States, January 1, 2019-May 30, 2020. *MMWR Morb Mortal Wkly Rep* 2020;**69**:699–704. doi:10.15585/mmwr.mm6923e1

19 Casalino E, Choquet C, Bouzid D, *et al.* Analysis of Emergency Department Visits and Hospital Activity during Influenza Season, COVID-19 Epidemic, and Lockdown Periods in View of Managing a Future Disaster Risk: A Multicenter Observational Study. *Int J Environ Res Public Health* 2020;**17**. doi:10.3390/ijerph17228302

20 Boserup B, McKenney M, Elkbuli A. The impact of the COVID-19 pandemic on emergency department visits and patient safety in the United States. *Am J Emerg Med* 2020;**38**:1732–6. doi:10.1016/j.ajem.2020.06.007

21 Kuitunen I, Ponkilainen VT, Launonen AP, *et al.* The effect of national lockdown due to COVID-19 on emergency department visits. *Scandinavian Journal of Trauma, Resuscitation and Emergency Medicine* 2020;**28**:114. doi:10.1186/s13049-020-00810-0

22 Jephcote C, Hansell AL, Adams K, *et al.* Changes in air quality during COVID-19 'lockdown' in the United Kingdom. *Environmental Pollution* 2021;**272**:116011. doi:10.1016/j.envpol.2020.116011

23 Pulia MS, Hekman DJ, Glazer JM, *et al.* Electronic Health Record-Based Surveillance for Community Transmitted COVID-19 in the Emergency Department. *West J Emerg Med* 2020;**21**:748–51. doi:10.5811/westjem.2020.5.47606

24 By the COVID-19 APHP-Universities-INRIA-INSERM. Early indicators of intensive care unit bed requirement during the COVID-19 epidemic: A retrospective study in Ile-de-France region, France. *PLOS ONE* 2020;**15**:e0241406. doi:10.1371/journal.pone.0241406

25 Sanchez B, Hirzel AH, Bingisser R, *et al.* State of Emergency Medicine in Switzerland: a national profile of emergency departments in 2006. *Int J Emerg Med* 2013;**6**:23. doi:10.1186/1865-1380-6-23

26 Mesnier J, Cottin Y, Coste P, *et al.* Hospital admissions for acute myocardial infarction before and after lockdown according to regional prevalence of COVID-19 and patient profile in France: a registry study. *The Lancet Public Health* 2020;**5**:e536–42. doi:10.1016/S2468-2667(20)30188-2

For peer review only - http://bmjopen.bmj.com/site/about/guidelines.xhtml

Figure legends

Figure 1. Time series surveillance indicators.

Figure 2. P-charts of emergency department flu-like syndrome during the COVID-19 outbreak at Lausanne University Hospital.

Figure 3. Time series daily emergency department general activity at Lausanne University Hospital.

For peer review only

For peer review only - http://bmjopen.bmj.com/site/about/guidelines.xhtml

Table 1. Patient characteristics

	COVID period 25 Feb 20 to 25 Feb 21		Previous period 25 Feb 19 to 24 Feb 20	
	N	% (95% CI)	N	% (95% CI)
Total ED visits	37,319		42,584	
ED flu-like syndrome	2,181	5.8 (5.6-6.1)	235	0.6 (0.5-0.6)
ED isolation droplet	4,124	11.1 (10.7-11.4)	510	1.2 (1.1-1.3)
ED respiratory syndrome	3,454	9.3 (9.0-9.6)	2,713	6.4 (6.1-6.6)
ED COVID-19 confirmed	1,421	3.8 (3.6-4.0)	-	-
ED visits				
Medicine	14,558	39.0 (38.5-39.5)	15,261	35.8 (35.4-36.3)
Surgery	3,098	8.3 (8.0-8.6)	3,799	8.9 (8.7-9.2)
Resuscitation room	2,340	6.3 (6.0-6.5)	2,201	5.2 (5.0-5.4)
Ambulatory care	18,491	49.5 (49.0-50.1)	22,038	51.8 (51.3-52.2)
Age (years)				
15 -29	6,429	17.2 (16.6-17.6)	8,567	20.1 (19.7-20.5)
30 -44	7,571	20.3 (19.9-20.7)	8,929	21.0 (20.6-21.4)
45 -54	4,574	12.3 (11.9-12.6)	5,223	12.3 (12.0-12.6)
55 – 64	4,957	13.3 (12.9-13.6)	5,258	12.3 (12.0-12.7)
65 -74	4,496	12.0 (11.7-12.4)	4,826	11.3 (11.0-11.6)
≥ 75	8,987	24.1 (23.6-24.5)	9,350	22.0 (21.6-22.4)
Gender (female)	17,171	46.0 (45.5-46.5)	19,745	46.4 (45.9-46.9)
Hospitalisation	15,325	41.1 (40.6-41.6)	15,545	36.5 (36.0-37.0)
ICU	615	1.7 (1.5-1.8)	562	1.3 (1.2-1.4)
MEWS ≥5	225	0.6 (0.5-0.7)	188	0.4 (0.4-0.5)
NEWS ≥5	1,028	2.7 (2.6-2.9)	849	2.0 (1.9-2.1)
Length of stay in ED ≥ 6 hours	18,926	50.7 (50.2-51.2)	22,581	53.0 (52.6-53.5)
Death in the ED	63	0.2 (0.1-0.2)	59	0.1 (0.1-0.2)

ED: emergency department; MEWS: Modified Early Warning Score; NEWS: National Early Warning Score; ICU: intensive care unit

For peer review only

For peer review only - http://bmjopen.bmj.com/site/about/guidelines.xhtml

Table 2. Correlation and time lag between surveillance indicators and ICU occupancy.

	Highest correlation Coefficient 95% CI	Time Lag 1* [days]	Time Lag 2* [days]	P value for Breusch-Godfrey test and Durbin's test	P value for Granger causality Lag 1	P value for Granger causality Lag 2
Confirmed cases	0.76 (0.67-0.83)	18	16	<.001	<.001	.901
Proportion of positive laboratory tests	0.92 (0.85-0.96)	15	20	<.001	<.001	.009
EMS call	0.47 (0.38-0.56)	20	7	<.001	<.001	.368
Ambulance dispatch	0.33 (0.25-0.42)	33	7	<.001	<.001	.221
ED droplet isolation	0.79 (0.71-0.86)	11	6	<.001	<.001	<.001
ED flu-like syndrome	0.73 (0.64-0.80)	13	7	<.001	<.001	<.001
ED COVID-19 confirmed	0.81 (0.73-0.88)	13	7	<.001	<.001	.020

*Lag 1 estimated by the highest correlation coefficient on correlograms and Lag 2 estimated by the lowest final prediction error (FPE) and the lowest Akaike's information criterion (AIC).

For peer review only

For peer review only - http://bmjopen.bmj.com/site/about/guidelines.xhtml

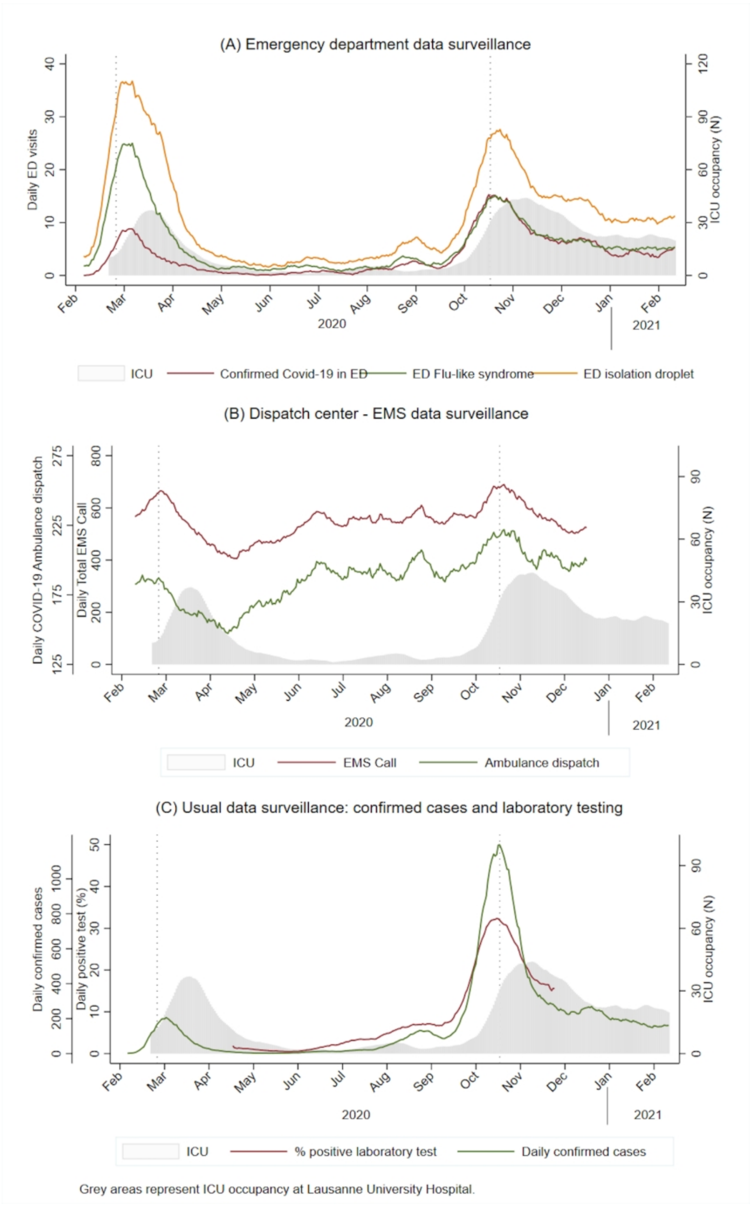

Figure 1. Time series surveillance indicators

119x192mm (300 x 300 DPI)

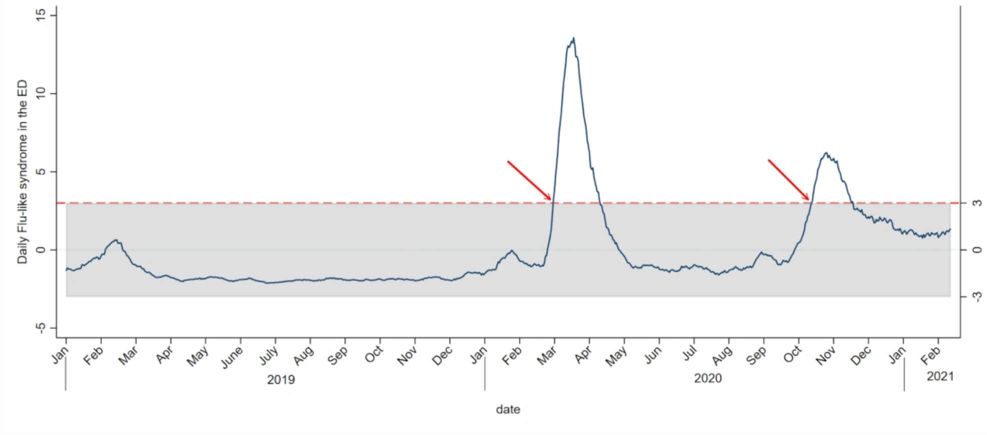

Figure 2. P-charts of emergency department flu-like syndrome during the COVID-19 outbreak at Lausanne University Hospital

202x90mm (300 x 300 DPI)

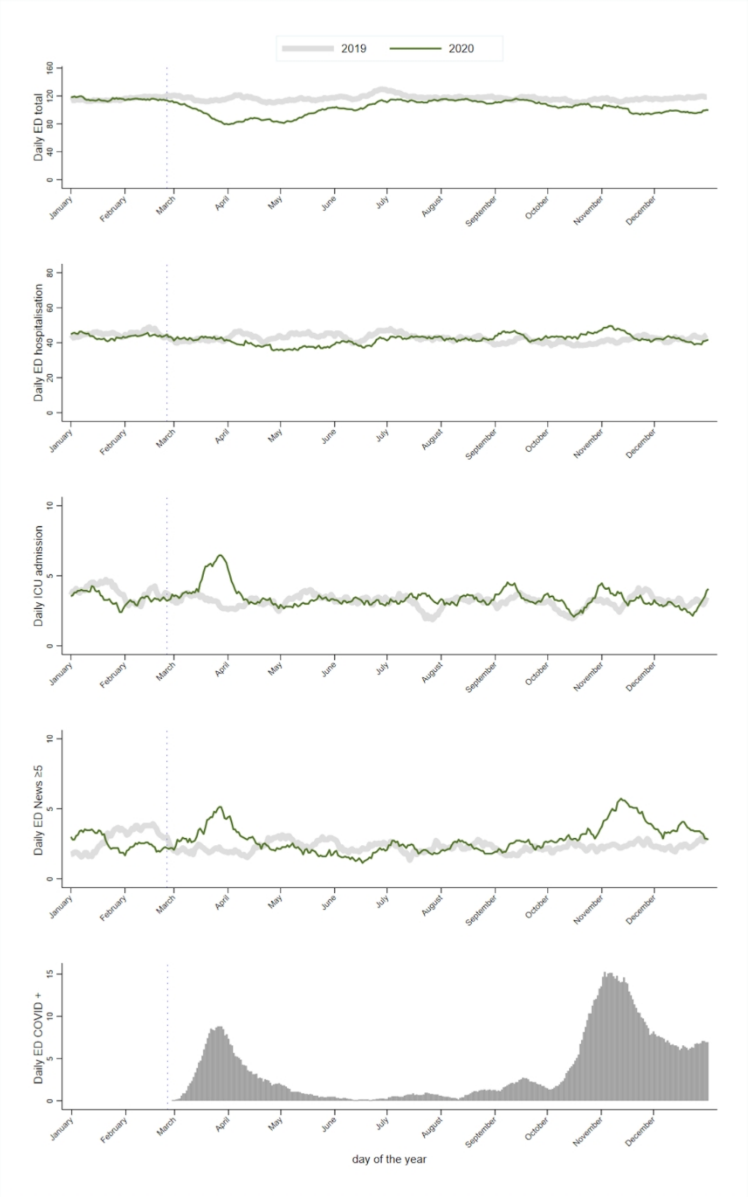

Figure 3. Time series daily emergency department general activity at Lausanne University Hospital

124x199mm (300 x 300 DPI)

Web Appendix

eSupplement 1. Method for Early Aberration Reporting System (STATA computing)

1. Identify the flu-like syndrome in emergency department software using the main complaint reported by the patient: combine the flu-like syndrome or fever/cough/shortness of breath and isolation droplet depending on the main complaint collected.

```
.gen flu_like_syndrome=1 if fever==1 | shortness_breath==1 | cough==1 & isolation_droplet
```

2. Extract the number of flu-like syndromes by day and the total number of patients attending the ED by day.

```
.collapse (sum) flu_like_syndrome total_ed id, by(date_admission)
```

3. Perform a moving average for the number of flu-like syndromes by day (7 days or less).

```
.tssmooth ma flu_like_sd_ma= flu_like_syndrome, window(7 1 7)
```

```
.tssmooth ma total_ed_ma= total_ed, window(7 1 7)
```

4. Plot the P-chart (using the usual limit $Pr \pm 3\sqrt{\frac{\mathrm{Pr}(1-\mathrm{Pr})}{N}}$).

```
. pchart flu_like_sd_ma date_admission total_ed_ma, sta recast(line) ytitle("Daily Flu-like syndrome in the ED",size(small)) title("P-chart moving average fraction defective", size(small)) xtitle("date",size(small)) ylabel(, labsize(small) axis(1)) ylabel(, labsize(small))
```


For peer review only - http://bmjopen.bmj.com/site/about/guidelines.xhtml

eSupplement 2. Correlogram between ICU occupancy and surveillance indicators at Lausanne University Hospital.

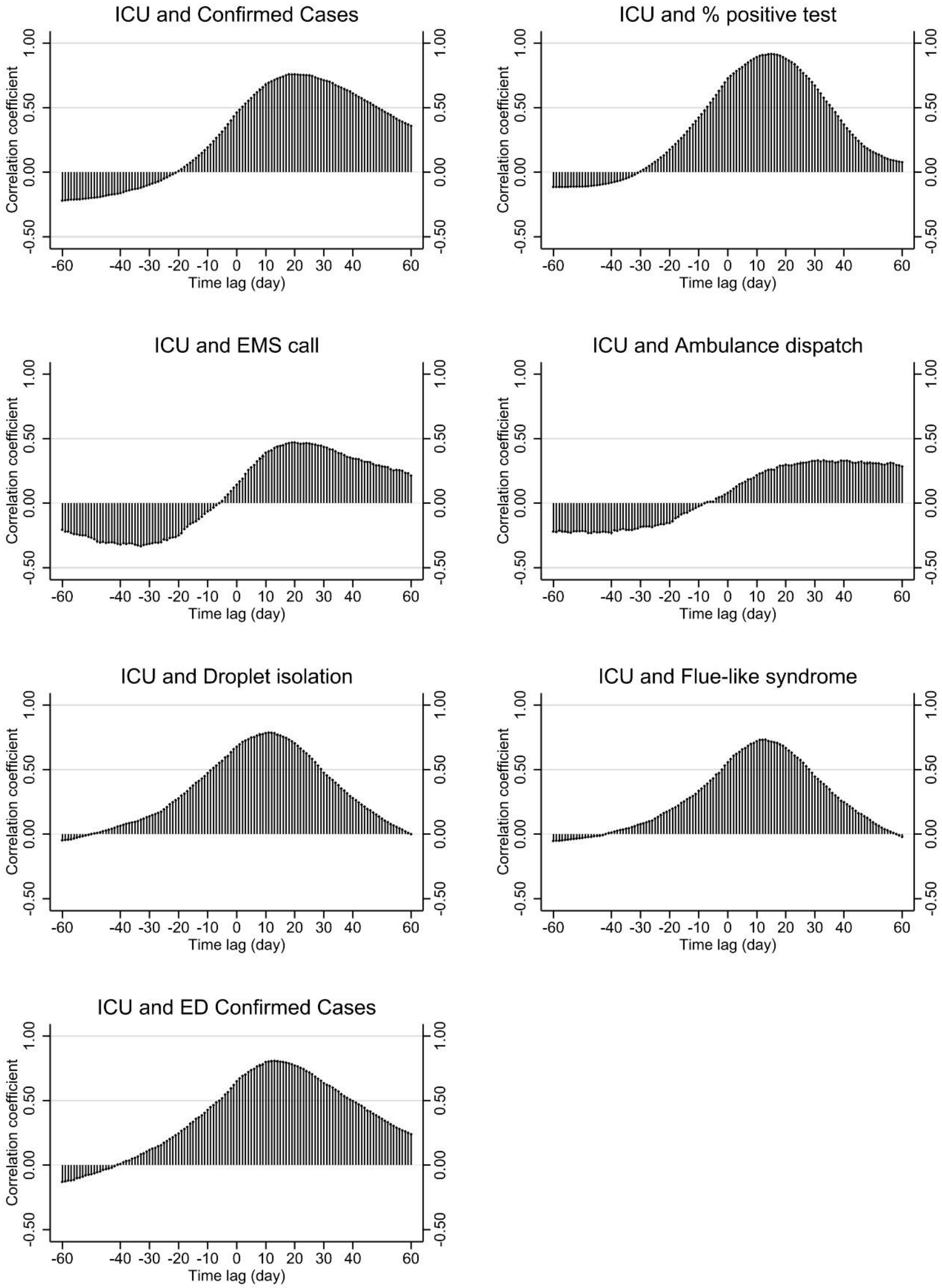

eSupplement 3. Daily P-charts of emergency department flu-like syndrome during the second wave of the COVID-19 outbreak at Lausanne University Hospital.

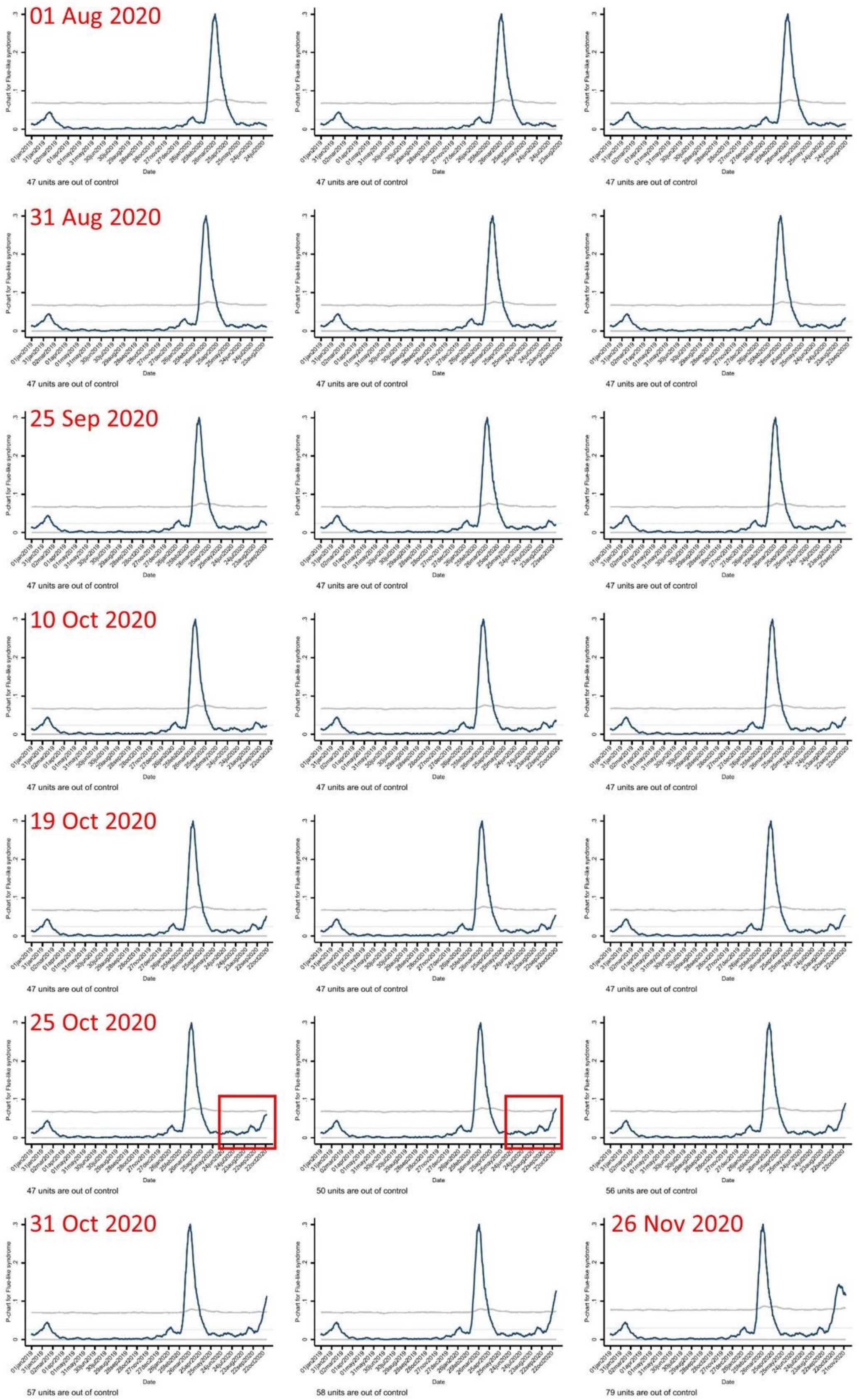

eSupplement 4. Time series by daily ED specific activity at Lausanne University Hospital (trauma, cardiology and stroke).

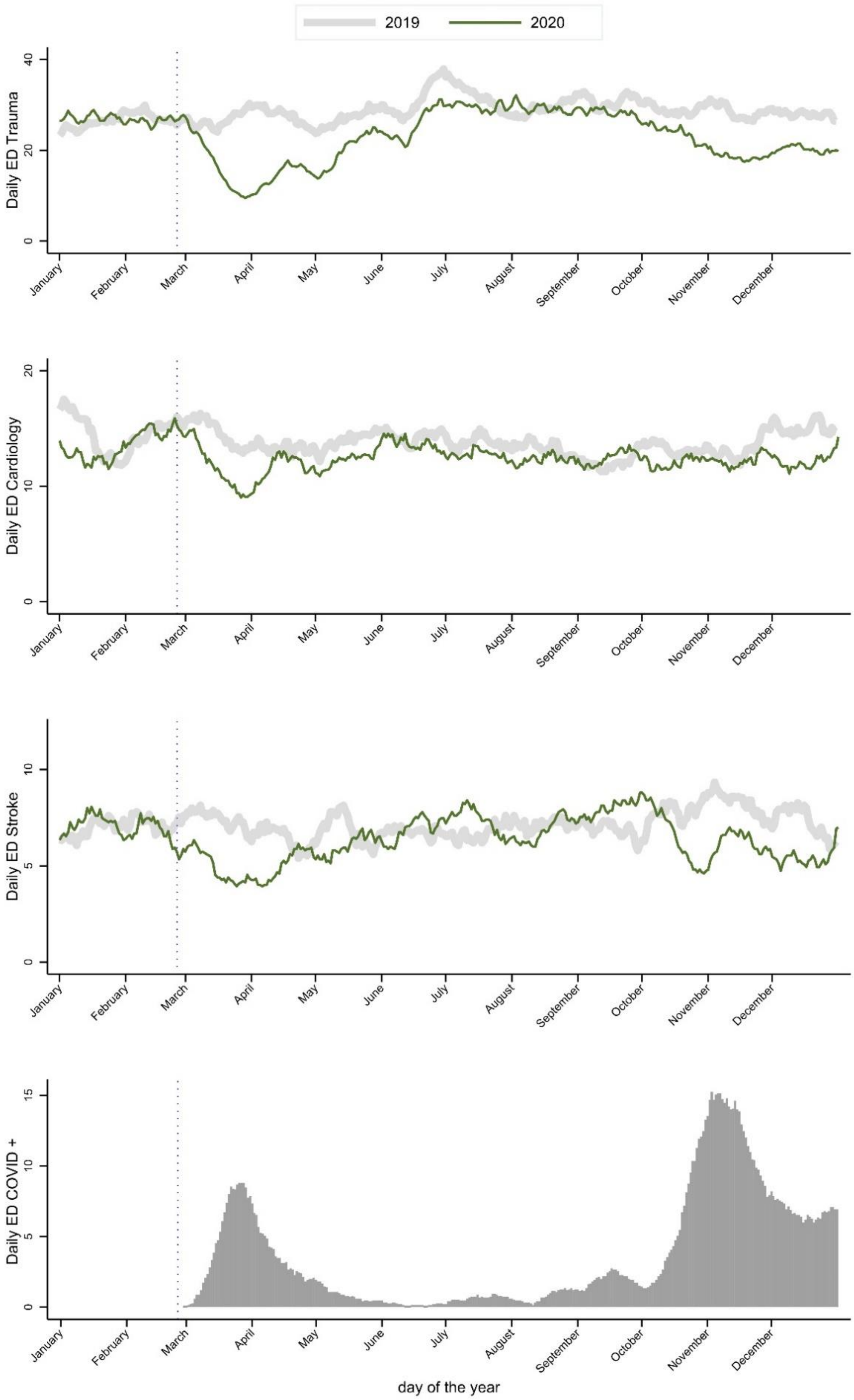

eSupplement 5. Time series by daily ED specific activity at Lausanne University Hospital (GIB, diabetes, allergy).

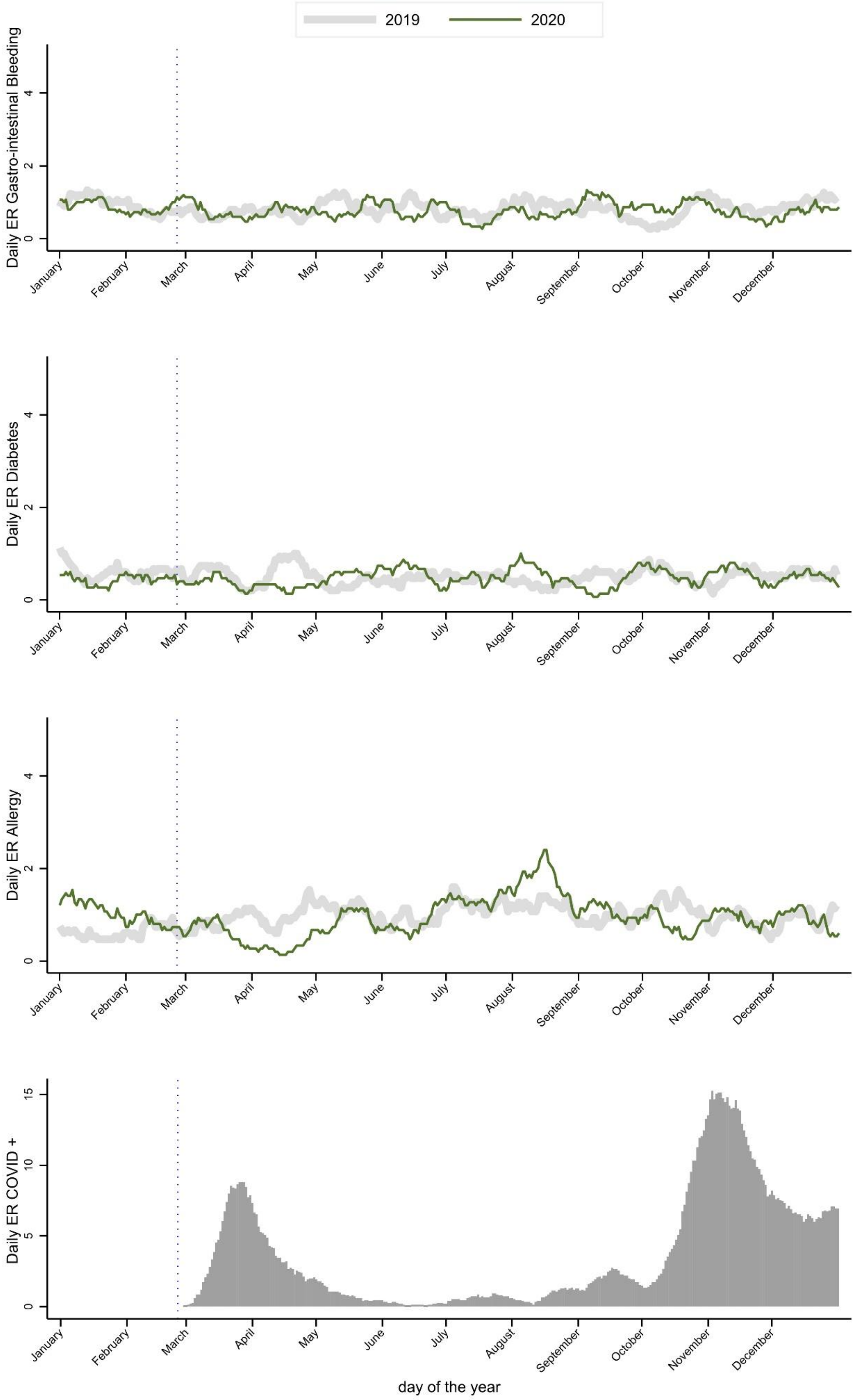

For peer review only - http://bmjopen.bmj.com/site/about/guidelines.xhtml

eSupplement 6. Time series by ED length of stay and waiting time at Lausanne University Hospital.

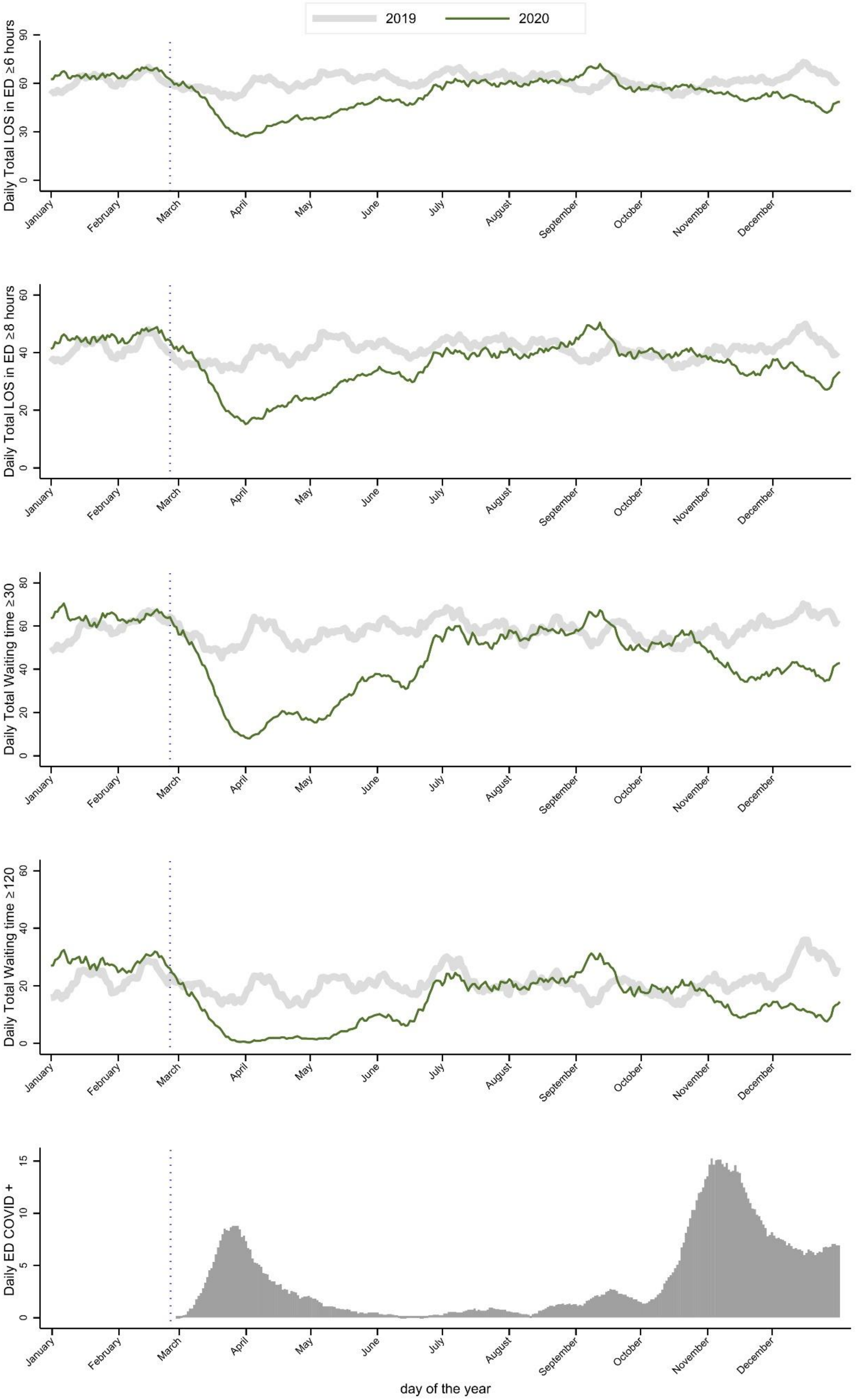

STROBE Statement—Checklist of items that should be included in reports of ***cross-sectional studies***

	Item No	Recommendation	Page No
**Title and abstract**	1	(*a*) Indicate the study's design with a commonly used term in the title or the abstract	1
		(*b*) Provide in the abstract an informative and balanced summary of what was done and what was found	2
**Introduction**			
Background/rationale	2	Explain the scientific background and rationale for the investigation being reported	4
Objectives	3	State specific objectives, including any prespecified hypotheses	4,5
**Methods**			
Study design	4	Present key elements of study design early in the paper	5
Setting	5	Describe the setting, locations, and relevant dates, including periods of recruitment, exposure, follow-up, and data collection	5
Participants	6	(*a*) Give the eligibility criteria, and the sources and methods of selection of participants	5
Variables	7	Clearly define all outcomes, exposures, predictors, potential confounders, and effect modifiers. Give diagnostic criteria, if applicable	6
Data sources/ measurement	8*	For each variable of interest, give sources of data and details of methods of assessment (measurement). Describe comparability of assessment methods if there is more than one group	6,7
Bias	9	Describe any efforts to address potential sources of bias	8
Study size	10	Explain how the study size was arrived at	NA
Quantitative variables	11	Explain how quantitative variables were handled in the analyses. If applicable, describe which groupings were chosen and why	NA
Statistical methods	12	(*a*) Describe all statistical methods, including those used to control for confounding	7,8
		(*b*) Describe any methods used to examine subgroups and interactions	7
		(*c*) Explain how missing data were addressed	8
		(*d*) If applicable, describe analytical methods taking account of sampling strategy	NA
		(*e*) Describe any sensitivity analyses	7,8
**Results**			
Participants	13*	(a) Report numbers of individuals at each stage of study—eg numbers potentially eligible, examined for eligibility, confirmed eligible, included in the study, completing follow-up, and analysed	8
		(b) Give reasons for non-participation at each stage	-
		(c) Consider use of a flow diagram	-
Descriptive data	14*	(a) Give characteristics of study participants (eg demographic, clinical, social) and information on exposures and potential confounders	8,9, table 1
		(b) Indicate number of participants with missing data for each variable of interest	Table 1
Outcome data	15*	Report numbers of outcome events or summary measures	8,9

For peer review only

Main results	16	(*a*) Give unadjusted estimates and, if applicable, confounder-adjusted estimates and their precision (eg, 95% confidence interval). Make clear which confounders were adjusted for and why they were included	Table 2, Fig 1, Fig 2
		(*b*) Report category boundaries when continuous variables were categorized	NA
		(*c*) If relevant, consider translating estimates of relative risk into absolute risk for a meaningful time period	NA
Other analyses	17	Report other analyses done—eg analyses of subgroups and interactions, and sensitivity analyses	Fig 3, Fig4, Web appendix
**Discussion**			
Key results	18	Summarise key results with reference to study objectives	9
Limitations	19	Discuss limitations of the study, taking into account sources of potential bias or imprecision. Discuss both direction and magnitude of any potential bias	11,12
Interpretation	20	Give a cautious overall interpretation of results considering objectives, limitations, multiplicity of analyses, results from similar studies, and other relevant evidence	10,11
Generalisability	21	Discuss the generalisability (external validity) of the study results	12
**Other information**			
Funding	22	Give the source of funding and the role of the funders for the present study and, if applicable, for the original study on which the present article is based	12

*Give information separately for exposed and unexposed groups.

Note: An Explanation and Elaboration article discusses each checklist item and gives methodological background and published examples of transparent reporting. The STROBE checklist is best used in conjunction with this article (freely available on the Web sites of PLoS Medicine at http://www.plosmedicine.org/, Annals of Internal Medicine at http://www.annals.org/, and Epidemiology at http://www.epidem.com/). Information on the STROBE Initiative is available at www.strobe-statement.org.

For peer review only

For peer review only - http://bmjopen.bmj.com/site/about/guidelines.xhtml

Lessons from COVID-19 syndromic surveillance through emergency department activity: a prospective time-series study from western Switzerland

Journal:	*BMJ Open*
Manuscript ID	bmjopen-2021-054504.R3
Article Type:	Original research
Date Submitted by the Author:	25-Mar-2022
Complete List of Authors:	AGERON, Francois-Xavier; Lausanne University Hospital, Emergency Department; University of Lausanne<br>Hugli, Olivier; Lausanne University Hospital, Emergency department<br>Dami, Fabrice; Lausanne University Hospital, Emergency Department<br>Caillet-Bois, David; Lausanne University Hospital, Emergency Department<br>Pittet, Valerie; University of Lausanne, Institute of Social and Preventive Medicine<br>Eckert, Philippe; Lausanne University Hospital<br>Beysard, Nicolas; Lausanne University Hospital, Emergency<br>Carron, Pierre-Nicolas; Lausanne University Hospital, Emergency
**Primary Subject Heading**:	Public health
Secondary Subject Heading:	Emergency medicine, Epidemiology, Infectious diseases, Intensive care
Keywords:	COVID-19, Public health < INFECTIOUS DISEASES, ACCIDENT & EMERGENCY MEDICINE

SCHOLARONE™
Manuscripts

I, the Submitting Author has the right to grant and does grant on behalf of all authors of the Work (as defined in the below author licence), an exclusive licence and/or a non-exclusive licence for contributions from authors who are: i) UK Crown employees; ii) where BMJ has agreed a CC-BY licence shall apply, and/or iii) in accordance with the terms applicable for US Federal Government officers or employees acting as part of their official duties; on a worldwide, perpetual, irrevocable, royalty-free basis to BMJ Publishing Group Ltd ("BMJ") its licensees and where the relevant Journal is co-owned by BMJ to the co-owners of the Journal, to publish the Work in this journal and any other BMJ products and to exploit all rights, as set out in our licence.

The Submitting Author accepts and understands that any supply made under these terms is made by BMJ to the Submitting Author unless you are acting as an employee on behalf of your employer or a postgraduate student of an affiliated institution which is paying any applicable article publishing charge ("APC") for Open Access articles. Where the Submitting Author wishes to make the Work available on an Open Access basis (and intends to pay the relevant APC), the terms of reuse of such Open Access shall be governed by a Creative Commons licence – details of these licences and which Creative Commons licence will apply to this Work are set out in our licence referred to above.

Other than as permitted in any relevant BMJ Author's Self Archiving Policies, I confirm this Work has not been accepted for publication elsewhere, is not being considered for publication elsewhere and does not duplicate material already published. I confirm all authors consent to publication of this Work and authorise the granting of this licence.

For peer review only

Lessons from COVID-19 syndromic surveillance through emergency department activity: a prospective time-series study from western Switzerland

François-Xavier Ageron[1], Oliver Hugli[1], Fabrice Dami[1], David Caillet-Bois[1], Valérie Pittet[2], Philippe Eckert[3], Nicolas Beysard[1], Pierre-Nicolas Carron[1]

1 Emergency Department, Lausanne University Hospital, 46 Rue du Bugnon, 1011 Lausanne, Switzerland

2 Centre for Primary Care and Public Health, 10 route de la Corniche, University of Lausanne, Lausanne, Switzerland

3 General Directorate, Lausanne University Hospital, 21 Rue du Bugnon, 1011 Lausanne, Switzerland

Submitted to**:** *BMJ open*

Word count: 2678; 3 figures; 2 tables

Corresponding author:

Dr. Francois-Xavier AGERON, MD, PhD

Emergency Department

Lausanne University Hospital

46 Rue du Bugnon

1011 Lausanne, Switzerland

Tel.: +41 79 556 88 69

E-mail: francois-xavier.ageron@chuv.ch

For peer review only

For peer review only - http://bmjopen.bmj.com/site/about/guidelines.xhtml

Abstract

Objective: We aimed to assess if emergency department (ED) syndromic surveillance during the first and second waves of the COVID-19 outbreak could have improved our surveillance system.

Design, settings: We did an observational study using aggregated data from the ED of a university hospital and public health authorities in western Switzerland.

Participants: All patients admitted to the ED were included.

Primary outcome measure: The main outcome was intensive care unit (ICU) occupancy. We used time series methods for ED syndromic surveillance (flu-like syndrome, droplet isolation) and usual indicators from public health authorities (new cases, proportion of positive tests in the population).

Results: Based on 37,319 ED visits during the COVID-19 outbreak, 1421 ED visits (3.8%) were positive for severe acute respiratory syndrome coronavirus 2 (SARS-CoV-2). Patients with flu-like syndrome or droplet isolation in the ED showed a similar correlation to ICU occupancy as confirmed cases in the general population, with a time lag of approximately 13 days (0.73 [95% CI 0·64-0.80]; 0.79 [95% CI 0.71-0.86]; and 0.76 [95% CI 0.67-0.83], respectively). The proportion of positive tests in the population showed the best correlation with ICU occupancy (0.95 [95% CI 0.85-0.96]).

Conclusion: ED syndromic surveillance is an effective tool to detect and monitor a COVID-19 outbreak and to predict hospital resource needs. It would have allowed to anticipate ICU occupancy by 13 days, including significant aberration detection at the beginning of the second wave.

Keywords: emergency department, COVID-19, surveillance system, public health

For peer review only - http://bmjopen.bmj.com/site/about/guidelines.xhtml

Article summary

Strengths and limitations of this study

- A major strength of our study is that we performed and compared time series of surveillance data from the emergency department and the usual surveillance system (health regional authority and laboratory surveillance) during the COVID-19 outbreak.
- Emergency department surveillance data was correlated with intensive care unit occupancy during the outbreak.
- Rigorous methods were applied to detect a significant signal of the second wave before intensive care unit saturation, such as the Early Aberration Reporting System using a Shewhart chart.
- A limitation is that despite a good correlation and early detection of a significant signal, our study lacks external validity.

For peer review only - http://bmjopen.bmj.com/site/about/guidelines.xhtml

Introduction

In early 2020, the World Health Organization declared the COVID-19 outbreak to be a public health emergency of international concern.[1] Europe was particularly badly hit in spring 2020. After a relative lull in the summer, a second wave occurred in Europe in autumn 2020. Switzerland was among the most affected countries during this period with a much higher COVID-19 incidence compared to the first wave and with a 7-day incidence higher than 600 confirmed cases per 100,000.[2–4] Even if the second wave was expected, its beginning, timing and magnitude were not fully anticipated by the public health authorities.

Current public health surveillance systems include laboratory tests, death rates, hospital-based surveillance, and sentinel networks in primary care. Usual surveillance reports use the notification rate of confirmed cases and deaths, laboratory tests, hospital and intensive care unit (ICU) admission and occupancy rates. Primary care sentinel surveillance collects syndromic symptoms related to seasonal flu-like syndrome. Emergency departments (ED) are uniquely positioned at the interface of the community and hospitals and could serve as early warning systems to identify emerging threats and support decisions of public health authorities.[5] However, only one-third of European countries include ED data in their syndromic surveillance.[6] Until now, this has not been the case in Switzerland where ED data sets have not been used to detect or monitor epidemic outbreaks and, more specifically, the COVID-19 outbreak.

From July to early September 2020, most European countries observed an increase in the incidence of COVID-19 in young people <35 years, but without any significant simultaneous increase in hospital and ICU occupancy. These two concurrent numbers potentially contributed to erroneously reassure public health and political authorities. In addition, a retrospective analysis indicated a persistent higher incidence at the end of the summer in Switzerland, particularly in the western region.[7] However, it remains unknown if the monitoring of cases admitted to the ED would have provided early predictive clues on the resurgence of the pandemic. The aim of our study was to assess if ED

For peer review only - http://bmjopen.bmj.com/site/about/guidelines.xhtml

For peer review only

syndromic surveillance during the first and second waves of the COVID-19 outbreak could have improved health surveillance and provided additional information for the earlier detection of outbreak signals.

Methods

Study design and population

We did an observational study to assess whether ED syndromic surveillance would have improved the management of the first and second waves of the COVID-19 outbreak in a health system in western Switzerland, based on routine data from the Canton of Vaud and Lausanne University Hospital. The first wave occurred in March 2020 and reached a peak at the beginning of April 2020. The first lockdown in Switzerland started on 17 March 2020 and ended on 11 May 2020. The second wave occurred in November 2020. There was no lockdown applied for the second wave, but some federal restrictions were applied from 3 November 2020, such as restaurant, bar, cinema, museum and library closures.

We used aggregated data from the ED of Lausanne University Hospital, one of the five university hospitals in Switzerland, located in the French-speaking region. It serves as a primary care hospital for the Lausanne area with a population of 250,000 inhabitants and as a tertiary hospital for western Switzerland with a population of 1 million inhabitants. The ED triage includes approximatively 65,000 adult patients per year, two-thirds of whom are admitted to the ED, and one-third to the primary care consultation.

We used data from all consecutive visits leading to ED admission from 25 February, 2019 to 19 January, 2020 (pre-COVID period used as a control period) and from 25 February, 2020 (date of the first infection due to severe acute respiratory syndrome coronavirus 2 [SARS-CoV-2] in Switzerland) to 25 February, 2021. Patients referred to the primary care consultation after ED triage were

For peer review only - http://bmjopen.bmj.com/site/about/guidelines.xhtml

excluded. We also considered aggregated data for the entire population of the Canton of Vaud collected by the emergency medical service (EMS) dispatch centre and the public health authorities.

Data collection

We collected aggregated data from the ED including date and hour of admission, age group in categories, gender, main complaints at the time of admission classified using the Swiss Emergency Triage Scale (SETS),[8] deaths in the ED, hospital admissions to the ward or intensive care unit (ICU), and positive COVID-19 test notification. The modified early warning score (MEWS) and the national early warning score (NEWS) were calculated from the initial triage vital signs.[9,10] Data were extracted from the ED patient flow management software (Gyroflux®, Lausanne University Hospital, Switzerland) including triage vital signs, symptoms, isolation, ED length of stay, COVID test result, discharge diagnosis, and destination of ED patients. Only patient flow aggregated data available in real time were collected, without additional data from medical records. In addition, data were collected from the EMS dispatch centre (‘Centrale d'appels sanitaires d'urgence 144’) of the Canton of Vaud, including daily emergency calls and ambulance dispatch. We also collected daily hospital occupancy for COVID-19 patients in general wards and the ICU, as well as data from the Vaud health authority surveillance system (notification of new cases) and laboratory surveillance (results of PCR and antigen tests).[11]

Outcome

We selected daily absolute ICU occupancy as the primary outcome. ICU beds are a scarce resource requiring trained staff and specific medical devices. The prediction and anticipation of critical care resources has been a key issue in the COVID-19 outbreak. We considered the absolute ICU bed occupancy and not the ICU occupancy rate as the total number of ICU beds regularly evolved during the pandemic, according to needs and available resources (i.e. an increase from 35 to 76 beds).

Surveillance indicators

For peer review only - http://bmjopen.bmj.com/site/about/guidelines.xhtml

We studied and compared 'usual' and ED-specific surveillance indicators for COVID-19. Usual surveillance indicators were: 1) number of new confirmed cases of COVID-19 in the population (notification to cantonal public health authorities by medical laboratories or a general practitioner based on a PCR or antigen test); and 2) laboratory surveillance with the proportion of positive tests (PCR and antigen) from all tests performed. ED surveillance indicators were: 1) number of confirmed cases of COVID-19 during ED stay (PCR test); 2) number of patients subjected to droplet isolation measures in the ED; 3) syndromic surveillance with flu-like syndrome in the ED at triage; 4) number of EMS calls; and 5) number of ambulance dispatches.

Data analysis

We applied time series analyses for ED COVID-19 visits and for syndromic surveillance, including infectious disease, respiratory disease, cardiac symptoms including chest pain, neurologic symptoms including acute paralysis, gastrointestinal bleeding, trauma, psychiatric disorders, and hyper- or hypoglycaemia. We plotted the time series of syndromic surveillance data during the COVID-19 period and compared these to the same period of 2019. We smoothed time-series curves based on the moving 7-day average. We compared graphically ED-EMS surveillance and usual surveillance in the general population and explored the relationship between ICU occupancy and ED-EMS surveillance and traditional surveillance indicators by cross-correlation, and plotted correlograms. We tested the correlation between time series using the Breusch-Godfrey test for higher-order serial correlation and Durbin's alternative test for serial correlation.[12,13] The time lag in days between surveillance indicators and ICU occupancy was determined by estimating which lag showed the highest correlation on correlograms. We performed a Vector Auto-Regression (VAR) model and considered the optimal time lag for the lowest final prediction error and the lowest Akaike's information criterion. We performed Granger-causality with a linear regression model and a VAR model to determine which indicator was the best to predict ICU occupancy.[14] Quality control charts were then used to detect early aberration in daily data. The Early Aberration Reporting System

For peer review only

For peer review only - http://bmjopen.bmj.com/site/about/guidelines.xhtml

(EARS) uses different methods for temporal aberration detection, including the Shewhart chart (P-chart), moving average, and variation of the cumulative sum.[15,16] To assess the usefulness of the ED surveillance system, we assessed graphically the moving average for ED flu-like syndrome aberrations detected by P-chart during the second wave. The P-chart measures the fraction of nonconforming units in a sample. The control limits for the P-chart were estimated based on the confidence interval of the estimated fraction of the event in the time period using the normal approximation. The formula for the upper and lower limit was: $\mathrm{Pr} \pm 3\sqrt{\frac{\mathrm{Pr}\,(1-\mathrm{Pr})}{N}}$ where Pr was the estimated fraction in the time period. Detection of aberration occurs when the value is outside the 99.5% confidence interval. We detail the method in the supplement file (eSupplement 1). We did not reported any missing value for syndromic surveillance in the ED (mandatory item in the software). The sample size was fixed during the study period. We estimated that the minimal sample size was 2,668 participants to have a 90% chance of detecting, as significant level of 5%, a difference in the correlation coefficient from 0.75 to 0.80. Data were analysed using Stata version 16.0 (StataCorp, College Station, TX, USA).

Patient and public involvement statement

Patients were not involved in the research question and in the design of the study.

Results

We collected 37,319 ED visits from 25 February 2020 to 25 February 2021 (COVID-19 period) and 42,584 ED visits from 25 February 2019 to 19 January 2020 (pre-COVID [control] period). We reported 1421 (3.8%) confirmed cases of COVID-19 during ED stay, 2181 (5.8%) flu-like syndromes, and 4124 (11.1%) ED visits with droplet isolation (table 1). An increase of flu-like syndromes was observed during the COVID-19 period. The frequency of ICU admission also increased during the COVID-19 period by 30% (OR 1.30; 95% CI 1.15-1.47; P<.001).

For peer review only - http://bmjopen.bmj.com/site/about/guidelines.xhtml

We plotted routine surveillance indicators (confirmed cases, laboratory surveillance and ICU occupancy) and emergency surveillance indicators (EMS and ED indicators) (figure 1). The frequency of positive laboratory tests and confirmed cases first immediately increased, followed by ED flu-like syndrome, ED isolation droplet, and confirmed ED COVID-19. All indicators followed exactly the trend in ICU occupancy with a time lag, depending on the indicators. ED flu-like syndrome and ED droplet isolation showed a higher increase in the first wave than the second wave compared to ED COVID-19-confirmed cases. All surveillance indicators, except the EMS total number of calls, showed a good correlation with ICU occupancy (table 2). Correlograms showed a positive correlation for all indicators during the second wave (eSupplement 2). The highest correlations between ED-EMS surveillance indicators and ICU occupancy were obtained with time lags of 10 to 13 days (table 2). A significant aberration was detected as of 8 March 2020 for the first wave and as of 25 October 2020 for the second wave (figure 2). Aberrations were detected more than three weeks before the maximum ICU occupancy was reached. A selection of daily P-charts for ED flu-like syndrome during the second wave are presented in the supplement (eSupplement 3).

Daily ED activity is presented in figure 3. The total number of ED visits decreased during the first and second waves compared to ED activity in the previous year. Hospital admission remained stable, with a slight increase during the second wave. The number of patients who presented an intermediate-to-high risk of critical care (NEWS ≥ 5) increased during the first and second waves. Compared to 2019, trauma, cardiology, and stroke activity decreased during the first wave and to a lesser extent during the second wave (eSupplement 4). Gastrointestinal bleeding and diabetes were unchanged during both waves (eSupplement 5). Allergy decreased during the spring lockdown and increased during the summer break. ED length of stay and waiting time decreased during the first wave (eSupplement 6). During the second wave, ED length of stay decreased on a smaller scale.

Discussion

For peer review only - http://bmjopen.bmj.com/site/about/guidelines.xhtml

Our study shows the potential for ED syndromic surveillance as an effective tool to detect and monitor COVID-19 outbreaks and to predict hospital resource needs. The ED surveillance system correlated with ICU occupancy and would have allowed to anticipate ICU occupancy by 11 to 13 days. Of note, it would have also enabled significant aberration detection at the beginning of the second wave. In addition, ED surveillance would provide useful information to plan hospital bed needs, including the number and severity of patients admitted to the ED, hospital and ICU admissions, and hospital resources required for trauma, cardiology and neurology patients.

Comparison with other studies

Similar to others, we found a decrease in the total number of ED visits during the first wave.[17–20] Many countries implemented a lockdown during the first wave of the outbreak that explained the decrease in ED visits to a large extent.[21] Importantly, our syndromic surveillance results allow to describe with finer granularity the change in ED activity. During lockdown, we observed a decrease of certain diseases associated with exposure to environmental factors, such as allergy or CO_2 emission. Jephcote et al also reported a change in air quality during lockdown in the United Kingdom.[22] Kuitunen et al showed that the volume of road traffic and ED visits decreased at the same time and we also observed a reduction in the number of minor and major traumatic injuries.[21] The COVID-19 outbreak well illustrated that a change in human activities contributing to pollution has an immediate effect on population health.

We showed that ED surveillance data was sufficiently accurate to detect changes in the epidemiology of the COVID-19 outbreak, based on our current system using syndromic flu-like presentations and isolation measures for droplet. In the USA, Pulia and al showed that the surveillance of patients placed in respiratory isolation for an acute respiratory infection was useful to identify and monitor trends during the pandemic.[23] In Paris (France), researchers found that ED visits and EMS calls were correlated with ICU admission, as was the proportion of positive PCR tests.[24] Similar to the

For peer review only - http://bmjopen.bmj.com/site/about/guidelines.xhtml

Paris study, we showed that ED surveillance predicts ICU occupancy with a time lag of 13 days and the proportion of positive laboratory tests with a lag of 15 days.

Clinical implications

ED visits have constantly risen during the last decade and one-fourth to one-third of the population visit an ED annually.[25] EDs have become an important player in the public health system and an interface between primary care and the hospital. Indeed, the ED represents today almost the only clinical pathway to unscheduled in-hospital care. For this reason, the ED has the potential to become a real-time observatory of public health if properly designed with well-defined indicators. Consequently, it is not surprising that ED surveillance would have been an effective tool to detect and monitor COVID-19 outbreak activity as it provides simple indicators for real-time monitoring that allow a rapid response from health care authorities.

Inside hospitals, ED surveillance would be useful to plan ICU or intermediate-care unit resources by predicting ICU occupancy with a significant time lag, specific to the epidemic. Additional syndromic surveillance for surgery and other medical specialities would also be helpful to reduce some activities and re-allocate resources where they are the most needed. Of note, ED surveillance would enable to detect the indirect consequences of a pandemic, such as the change in ED visits for life-threatening conditions. It is unlikely that myocardial infarction and strokes decreased during lockdown, but the decrease in chest pain and stroke symptoms observed in the ED suggests that patients avoided attending the ED as a consequence of the 'stay-at-home' campaign and fear of nosocomial COVID-19 infection.[26] This type of ED surveillance data could incite health authorities to inform the population to alert emergency services in case of chest pain and stroke symptoms, regardless of the COVID-19 outbreak.

Strengths and weaknesses

Our study has some strengths and weaknesses. First, we used a well-described cohort of consecutive ED patient visits without missing data for outcome, syndromic surveillance, and triage severity.

For peer review only - http://bmjopen.bmj.com/site/about/guidelines.xhtml

Follow-up was complete. Second, we used simple observations to assess the obvious correlation between ICU occupancy and surveillance indicators in the first and second waves of the COVID-19 outbreak. We confirmed these observations with rigorous methods used in econometric science and in studies on surveillance systems. Third, we excluded patients attending at ED triage and requiring primary care that might lead to selection bias. However, the objective of the study was to use 'real-life data' available in 'real time' to predict ICU occupancy and detect aberration in syndromic surveillance. Fourth, even if P-charts and the correlation between ICU occupancy and ED surveillance data are obvious in retrospect, surveillance data could be difficult to interpret at the initial stage of a new pandemic and studies to assess the ability of ED surveillance systems to detect a potential new threat need to be performed prospectively in real time. Fifth, our study lacks external validity. The results are dependent on the health care system, hospital resources, and the triage criteria used in our ED.

In conclusion, ED syndromic surveillance provides additional effective information not accessible in the usual surveillance system. The real-time availability of data makes ED syndromic surveillance a powerful tool for health care and political authorities. Future studies on the potential role of emergency services as a public health observatory are needed to further demonstrate their ability to detect and provide data on a larger scale, such as at national level or in situations of infectious diseases, but also in non-infectious diseases related to toxicological, meteorological or psychological diseases.

For peer review only

For peer review only - http://bmjopen.bmj.com/site/about/guidelines.xhtml

The Corresponding Author has the right to grant on behalf of all authors and does grant on behalf of all authors, *a worldwide licence* to the Publishers and its licensees in perpetuity, in all forms, formats and media (whether known now or created in the future), to i) publish, reproduce, distribute, display and store the Contribution, ii) translate the Contribution into other languages, create adaptations, reprints, include within collections and create summaries, extracts and/or, abstracts of the Contribution, iii) create any other derivative work(s) based on the Contribution, iv) to exploit all subsidiary rights in the Contribution, v) the inclusion of electronic links from the Contribution to third party material where-ever it may be located; and, vi) licence any third party to do any or all of the above.

Competing interests: All authors have completed the ICMJE uniform disclosure form at www.icmje.org/coi_disclosure.pdf and declare no support from any organisation for the submitted work; no financial relationships with any organisations that might have an interest in the submitted work in the previous three years; no other relationships or activities that could appear to have influenced the submitted work.

Funding: This study received no funding.

Author Contributions: FXA and PNC designed the study. DCB and FXA were responsible for the data management plan. DCB, FD and VP extracted data. FXA was responsible for data analysis. OH, PNC and FXA interpreted data and drafted the manuscript. NB, VP, DCB, FD, PE, OH, PNC and FXA contributed to the interpretation of the results, critical revision of the manuscript, and approved the final version. NB, VP, DCB, FD, PE, OH, PNC and FXA agreed to be accountable for all aspects of the work.

Ethical approval: This study was assessed by the Ethics Review Board of the Canton of Vaud (reference number CER-VD 2020-00731) that decided to waive need for approval and need for inform consent as this study collected only aggregate data and no individual patient data level.

For peer review only

Data sharing: Data are available on reasonable request and with agreement from Lausanne University Hospital and Public Health Authorities of the Canton of Vaud.

Acknowledgments: The authors thank Rosemary Sudan for editorial assistance.

For peer review only

References

1 WHO. COVID-19 Public Health Emergency of International Concern (PHEIC) global research and innovation forum : towards a research roadmap. *Geneva; United Nation* 2020;:7.

2 Salathé M, Althaus CL, Neher R, *et al.* COVID-19 epidemic in Switzerland: on the importance of testing, contact tracing and isolation. *Swiss Medical Weekly* 2020;**150**. doi:10.4414/smw.2020.20225

3 Nørgaard SK, Vestergaard LS, Nielsen J, *et al.* Real-time monitoring shows substantial excess all-cause mortality during second wave of COVID-19 in Europe, October to December 2020. *Euro Surveill* 2021;**26**. doi:10.2807/1560-7917.ES.2021.26.1.2002023

4 Switzerland: WHO Coronavirus Disease (COVID-19) Dashboard. https://covid19.who.int/region/euro/country/ch (accessed 24 Mar 2021).

5 Hughes HE, Hughes TC, Morbey R, *et al.* Emergency department use during COVID-19 as described by syndromic surveillance. *Emerg Med J* 2020;**37**:600–4. doi:10.1136/emermed-2020-209980

6 Hughes HE, Edeghere O, O'Brien SJ, *et al.* Emergency department syndromic surveillance systems: a systematic review. *BMC Public Health* 2020;**20**:1891. doi:10.1186/s12889-020-09949-y

7 Haag C, Höglinger M, Moser A, *et al.* Social mixing and risk exposures for SARS-CoV-2 infections in elderly persons. *Swiss Medical Weekly* 2020;**150**. doi:10.4414/smw.2020.20416

8 Rutschmann OT, Hugli OW, Marti C, *et al.* Reliability of the revised Swiss Emergency Triage Scale: a computer simulation study. *Eur J Emerg Med* 2018;**25**:264–9. doi:10.1097/MEJ.0000000000000449

9 Subbe CP, Kruger M, Rutherford P, *et al.* Validation of a modified Early Warning Score in medical admissions. *QJM* 2001;**94**:521–6. doi:10.1093/qjmed/94.10.521

10 Smith GB, Prytherch DR, Meredith P, *et al.* The ability of the National Early Warning Score (NEWS) to discriminate patients at risk of early cardiac arrest, unanticipated intensive care unit admission, and death. *Resuscitation* 2013;**84**:465–70. doi:10.1016/j.resuscitation.2012.12.016

11 Gossin M, Walther D, Blanco J-M, *et al.* [SICOVID: a cantonal COVID information system for public health decision-making]. *Rev Med Suisse* 2020;**16**:2177–82.

12 hamilton J Douglas. *Time Series Analysis*. Princeton University Press. Princeton University Press 1994. https://press.princeton.edu/books/hardcover/9780691042893/time-series-analysis (accessed 18 Mar 2021).

13 Brown RL, Durbin J, Evans JM. Techniques for Testing the Constancy of Regression Relationships Over Time. *Journal of the Royal Statistical Society: Series B (Methodological)* 1975;**37**:149–63. doi:https://doi.org/10.1111/j.2517-6161.1975.tb01532.x

14 Rossi B, Wang Y. Vector autoregressive-based Granger causality test in the presence of instabilities. *The Stata Journal* 2019;**19**:883–99. doi:10.1177/1536867X19893631

For peer review only - http://bmjopen.bmj.com/site/about/guidelines.xhtml

15 Hutwagner L, Thompson W, Seeman GM, *et al.* The bioterrorism preparedness and response Early Aberration Reporting System (EARS). *J Urban Health* 2003;**80**:i89-96. doi:10.1007/pl00022319

16 Lawson BM, Fitzhugh EC, Hall SP, *et al.* Multifaceted Syndromic Surveillance in a Public Health Department Using the Early Aberration Reporting System. *Journal of Public Health Management and Practice* 2005;**11**:274–281.

17 Jeffery MM, D'Onofrio G, Paek H, *et al.* Trends in Emergency Department Visits and Hospital Admissions in Health Care Systems in 5 States in the First Months of the COVID-19 Pandemic in the US. *JAMA Intern Med* 2020;**180**:1328–33. doi:10.1001/jamainternmed.2020.3288

18 Hartnett KP, Kite-Powell A, DeVies J, *et al.* Impact of the COVID-19 Pandemic on Emergency Department Visits - United States, January 1, 2019-May 30, 2020. *MMWR Morb Mortal Wkly Rep* 2020;**69**:699–704. doi:10.15585/mmwr.mm6923e1

19 Casalino E, Choquet C, Bouzid D, *et al.* Analysis of Emergency Department Visits and Hospital Activity during Influenza Season, COVID-19 Epidemic, and Lockdown Periods in View of Managing a Future Disaster Risk: A Multicenter Observational Study. *Int J Environ Res Public Health* 2020;**17**. doi:10.3390/ijerph17228302

20 Boserup B, McKenney M, Elkbuli A. The impact of the COVID-19 pandemic on emergency department visits and patient safety in the United States. *Am J Emerg Med* 2020;**38**:1732–6. doi:10.1016/j.ajem.2020.06.007

21 Kuitunen I, Ponkilainen VT, Launonen AP, *et al.* The effect of national lockdown due to COVID-19 on emergency department visits. *Scandinavian Journal of Trauma, Resuscitation and Emergency Medicine* 2020;**28**:114. doi:10.1186/s13049-020-00810-0

22 Jephcote C, Hansell AL, Adams K, *et al.* Changes in air quality during COVID-19 'lockdown' in the United Kingdom. *Environmental Pollution* 2021;**272**:116011. doi:10.1016/j.envpol.2020.116011

23 Pulia MS, Hekman DJ, Glazer JM, *et al.* Electronic Health Record-Based Surveillance for Community Transmitted COVID-19 in the Emergency Department. *West J Emerg Med* 2020;**21**:748–51. doi:10.5811/westjem.2020.5.47606

24 By the COVID-19 APHP-Universities-INRIA-INSERM. Early indicators of intensive care unit bed requirement during the COVID-19 epidemic: A retrospective study in Ile-de-France region, France. *PLOS ONE* 2020;**15**:e0241406. doi:10.1371/journal.pone.0241406

25 Sanchez B, Hirzel AH, Bingisser R, *et al.* State of Emergency Medicine in Switzerland: a national profile of emergency departments in 2006. *Int J Emerg Med* 2013;**6**:23. doi:10.1186/1865-1380-6-23

26 Mesnier J, Cottin Y, Coste P, *et al.* Hospital admissions for acute myocardial infarction before and after lockdown according to regional prevalence of COVID-19 and patient profile in France: a registry study. *The Lancet Public Health* 2020;**5**:e536–42. doi:10.1016/S2468-2667(20)30188-2

For peer review only - http://bmjopen.bmj.com/site/about/guidelines.xhtml

Figure legends

Figure 1. Time series surveillance indicators.

Figure 2. P-charts of emergency department flu-like syndrome during the COVID-19 outbreak at Lausanne University Hospital.

Figure 3. Time series daily emergency department general activity at Lausanne University Hospital.

For peer review only

For peer review only - http://bmjopen.bmj.com/site/about/guidelines.xhtml

Table 1. Patient characteristics

	COVID period 25 Feb 20 to 25 Feb 21		Previous period 25 Feb 19 to 24 Feb 20	
	N	% (95% CI)	N	% (95% CI)
Total ED visits	37,319		42,584	
ED flu-like syndrome	2,181	5.8 (5.6-6.1)	235	0.6 (0.5-0.6)
ED isolation droplet	4,124	11.1 (10.7-11.4)	510	1.2 (1.1-1.3)
ED respiratory syndrome	3,454	9.3 (9.0-9.6)	2,713	6.4 (6.1-6.6)
ED COVID-19 confirmed	1,421	3.8 (3.6-4.0)	-	-
ED visits				
Medicine	14,558	39.0 (38.5-39.5)	15,261	35.8 (35.4-36.3)
Surgery	3,098	8.3 (8.0-8.6)	3,799	8.9 (8.7-9.2)
Resuscitation room	2,340	6.3 (6.0-6.5)	2,201	5.2 (5.0-5.4)
Ambulatory care	18,491	49.5 (49.0-50.1)	22,038	51.8 (51.3-52.2)
Age (years)				
15 -29	6,429	17.2 (16.6-17.6)	8,567	20.1 (19.7-20.5)
30 -44	7,571	20.3 (19.9-20.7)	8,929	21.0 (20.6-21.4)
45 -54	4,574	12.3 (11.9-12.6)	5,223	12.3 (12.0-12.6)
55 – 64	4,957	13.3 (12.9-13.6)	5,258	12.3 (12.0-12.7)
65 -74	4,496	12.0 (11.7-12.4)	4,826	11.3 (11.0-11.6)
≥ 75	8,987	24.1 (23.6-24.5)	9,350	22.0 (21.6-22.4)
Gender (female)	17,171	46.0 (45.5-46.5)	19,745	46.4 (45.9-46.9)
Hospitalisation	15,325	41.1 (40.6-41.6)	15,545	36.5 (36.0-37.0)
ICU	615	1.7 (1.5-1.8)	562	1.3 (1.2-1.4)
MEWS ≥5	225	0.6 (0.5-0.7)	188	0.4 (0.4-0.5)
NEWS ≥5	1,028	2.7 (2.6-2.9)	849	2.0 (1.9-2.1)
Length of stay in ED ≥ 6 hours	18,926	50.7 (50.2-51.2)	22,581	53.0 (52.6-53.5)
Death in the ED	63	0.2 (0.1-0.2)	59	0.1 (0.1-0.2)

ED: emergency department; MEWS: Modified Early Warning Score; NEWS: National Early Warning Score; ICU: intensive care unit

For peer review only - http://bmjopen.bmj.com/site/about/guidelines.xhtml

For peer review only

Table 2. Correlation and time lag between surveillance indicators and ICU occupancy.

	Highest correlation Coefficient 95% CI	Time Lag 1* [days]	Time Lag 2* [days]	P value for Breusch-Godfrey test and Durbin's test	P value for Granger causality	
					Lag 1	Lag 2
Confirmed cases	0.76 (0.67-0.83)	18	16	<.001	<.001	.901
Proportion of positive laboratory tests	0.92 (0.85-0.96)	15	20	<.001	<.001	.009
EMS call	0.47 (0.38-0.56)	20	7	<.001	<.001	.368
Ambulance dispatch	0.33 (0.25-0.42)	33	7	<.001	<.001	.221
ED droplet isolation	0.79 (0.71-0.86)	11	6	<.001	<.001	<.001
ED flu-like syndrome	0.73 (0.64-0.80)	13	7	<.001	<.001	<.001
ED COVID-19 confirmed	0.81 (0.73-0.88)	13	7	<.001	<.001	.020

*Lag 1 estimated by the highest correlation coefficient on correlograms and Lag 2 estimated by the lowest final prediction error (FPE) and the lowest Akaike's information criterion (AIC).

For peer review only

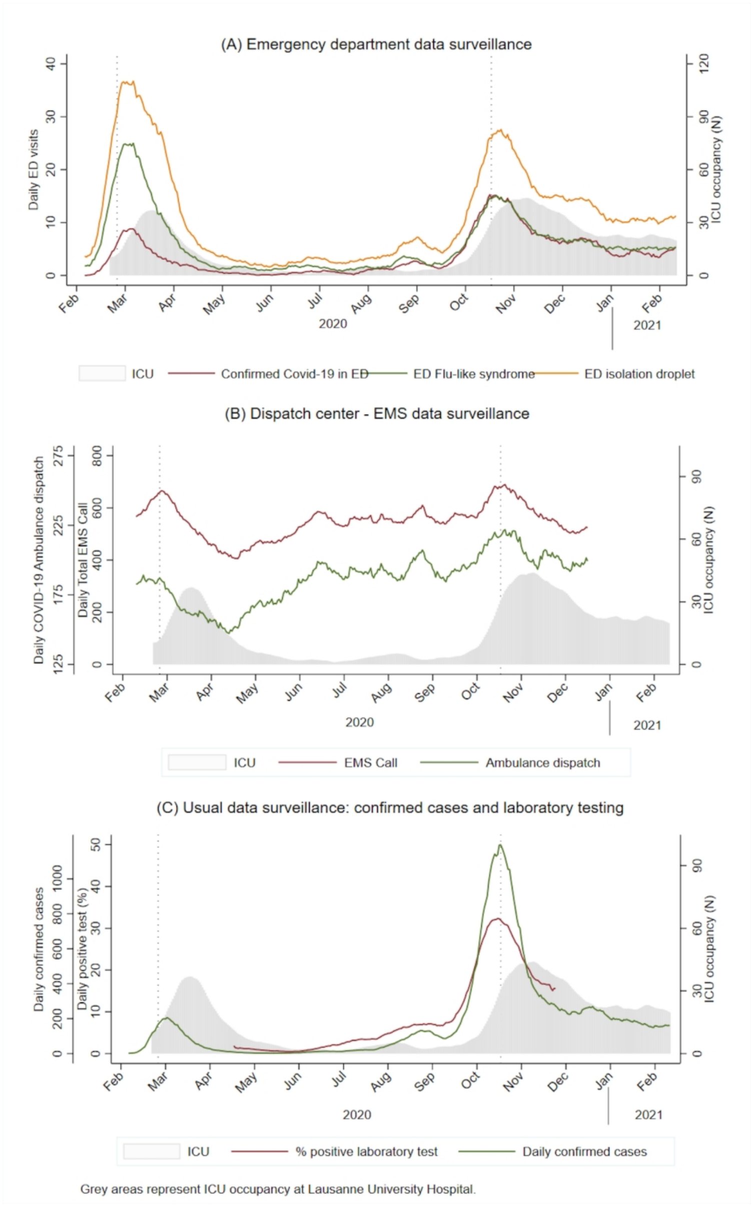

Figure 1. Time series surveillance indicators

119x192mm (300 x 300 DPI)

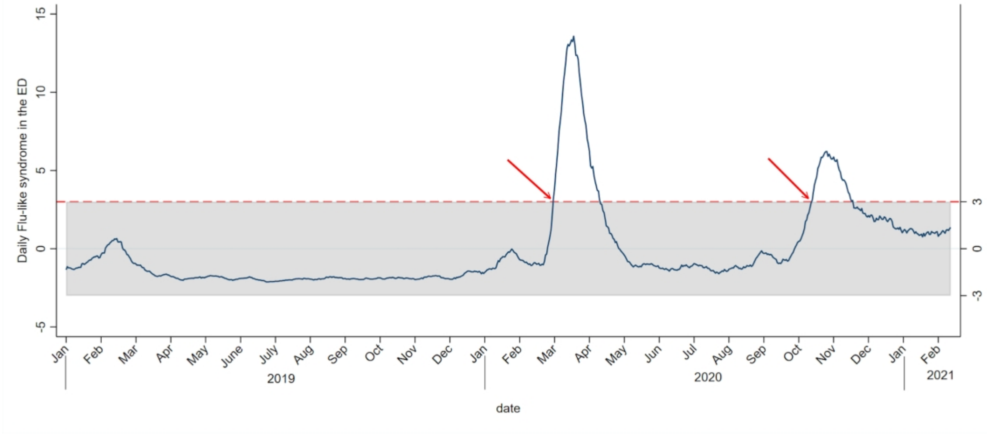

Figure 2. P-charts of emergency department flu-like syndrome during the COVID-19 outbreak at Lausanne University Hospital

202x90mm (300 x 300 DPI)

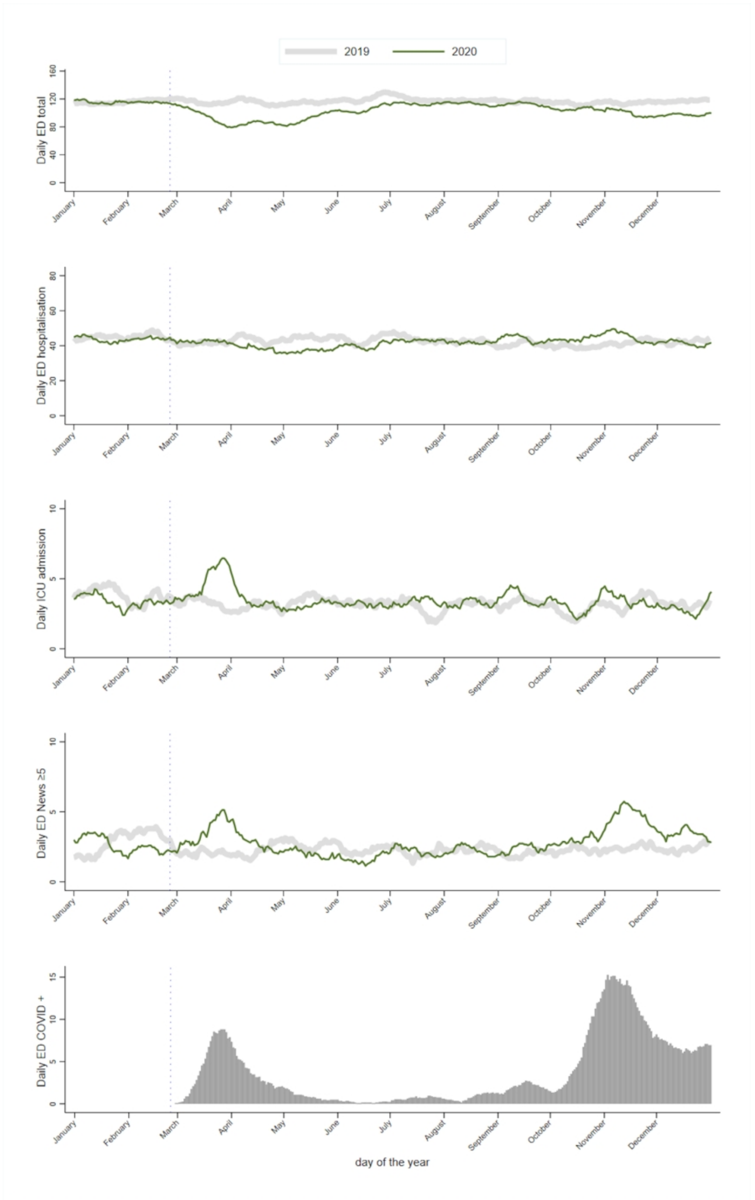

Figure 3. Time series daily emergency department general activity at Lausanne University Hospital

124x199mm (300 x 300 DPI)

Web Appendix

eSupplement 1. Method for Early Aberration Reporting System (STATA computing)

1. Identify the flu-like syndrome in emergency department software using the main complaint reported by the patient: combine the flu-like syndrome or fever/cough/shortness of breath and isolation droplet depending on the main complaint collected.

```
.gen flu_like_syndrome=1 if fever==1 | shortness_breath==1 | cough==1 & isolation_droplet
```

2. Extract the number of flu-like syndromes by day and the total number of patients attending the ED by day.

```
.collapse (sum) flu_like_syndrome total_ed id, by(date_admission)
```

3. Perform a moving average for the number of flu-like syndromes by day (7 days or less).

```
.tssmooth ma flu_like_sd_ma= flu_like_syndrome, window(7 1 7)
```

```
.tssmooth ma total_ed_ma= total_ed, window(7 1 7)
```

4. Plot the P-chart (using the usual limit $\Pr \pm 3\sqrt{\frac{\Pr(1-\Pr)}{N}}$).

```
. pchart flu_like_sd_ma date_admission total_ed_ma, sta recast(line) ytitle("Daily Flu-like syndrome in the ED",size(small)) title("P-chart moving average fraction defective", size(small)) xtitle("date",size(small)) ylabel(, labsize(small) axis(1)) ylabel(, labsize(small))
```


For peer review only - http://bmjopen.bmj.com/site/about/guidelines.xhtml

eSupplement 2. Correlogram between ICU occupancy and surveillance indicators at Lausanne University Hospital.

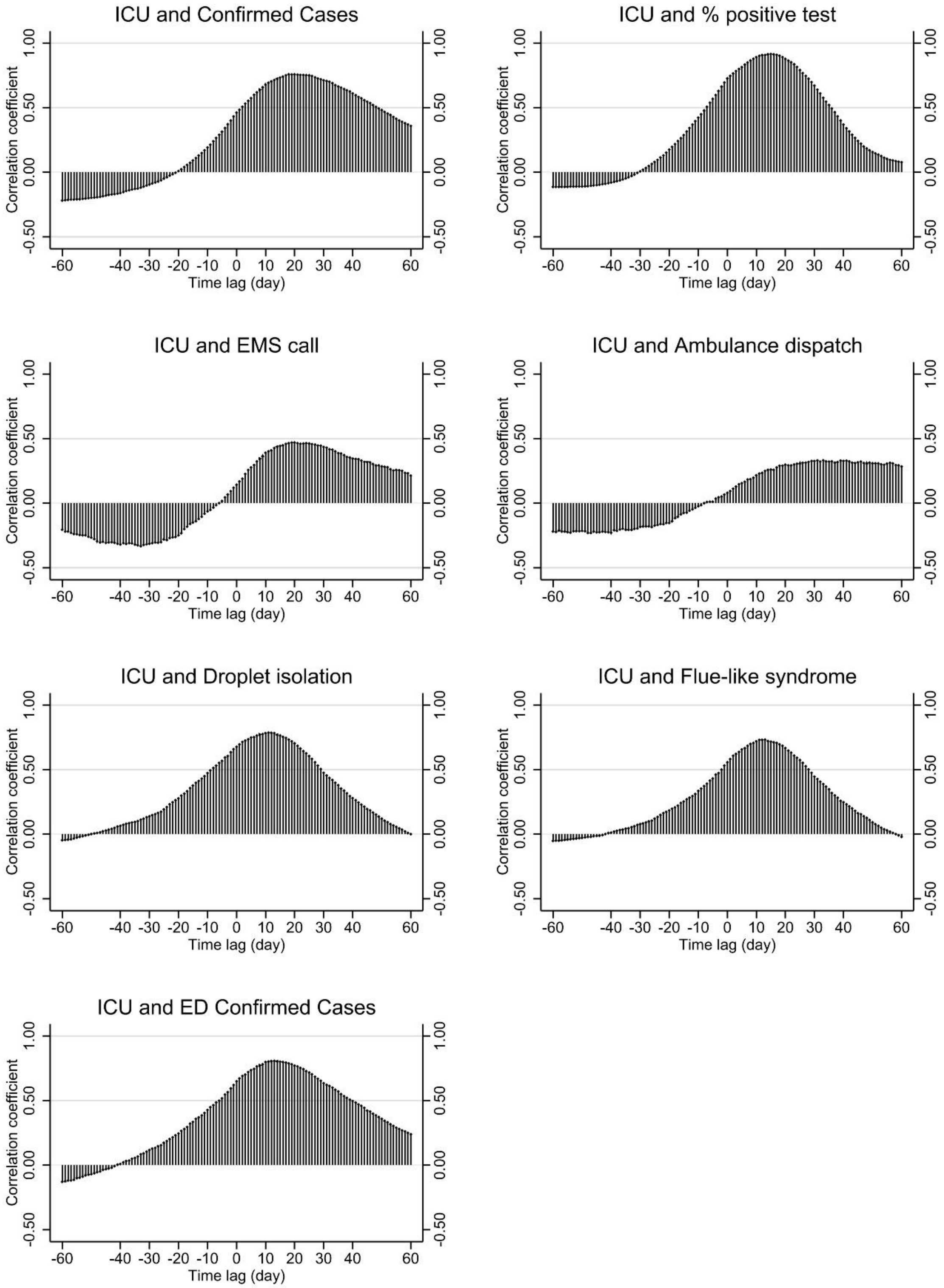

eSupplement 3. Daily P-charts of emergency department flu-like syndrome during the second wave of the COVID-19 outbreak at Lausanne University Hospital.

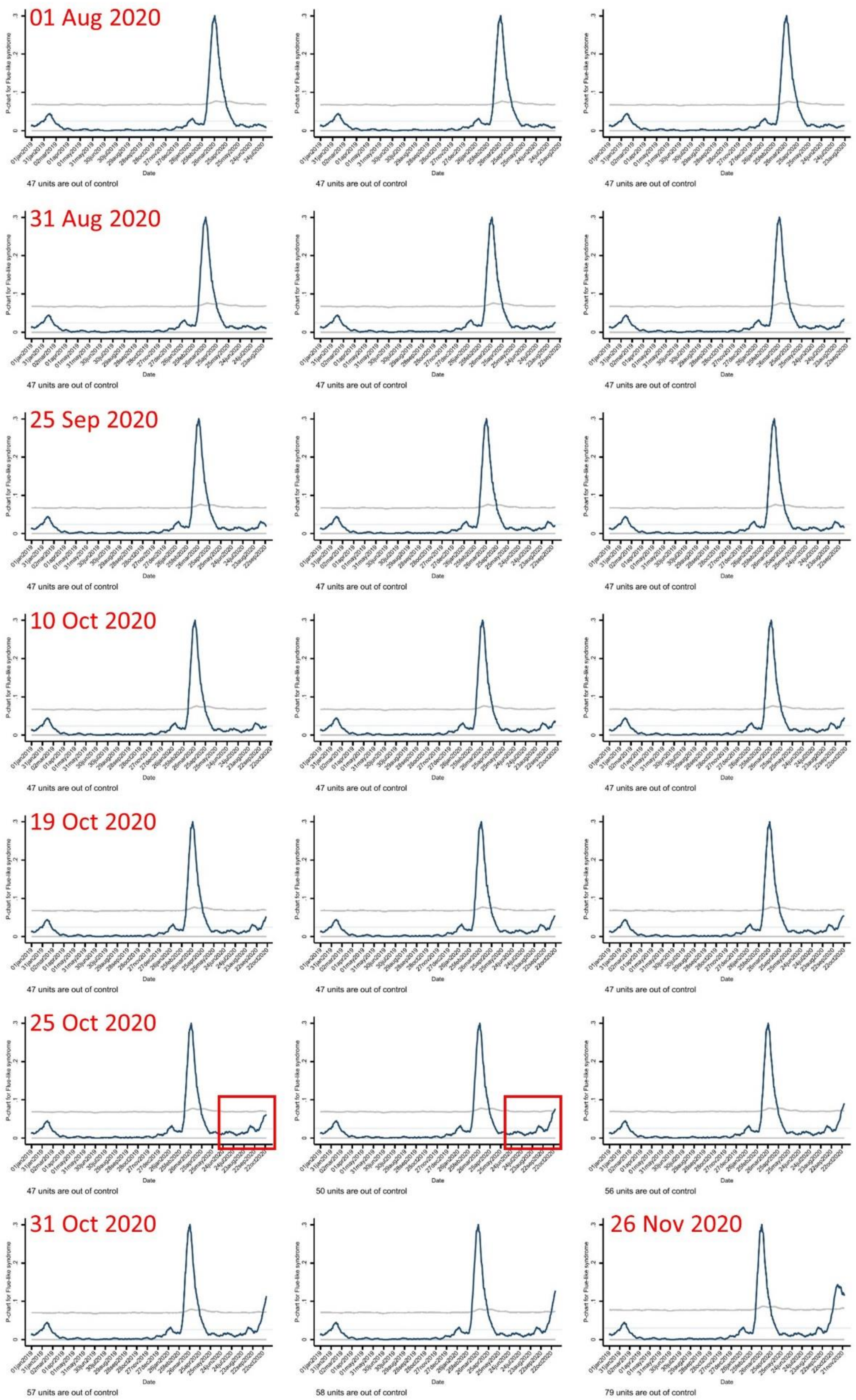

eSupplement 4. Time series by daily ED specific activity at Lausanne University Hospital (trauma, cardiology and stroke).

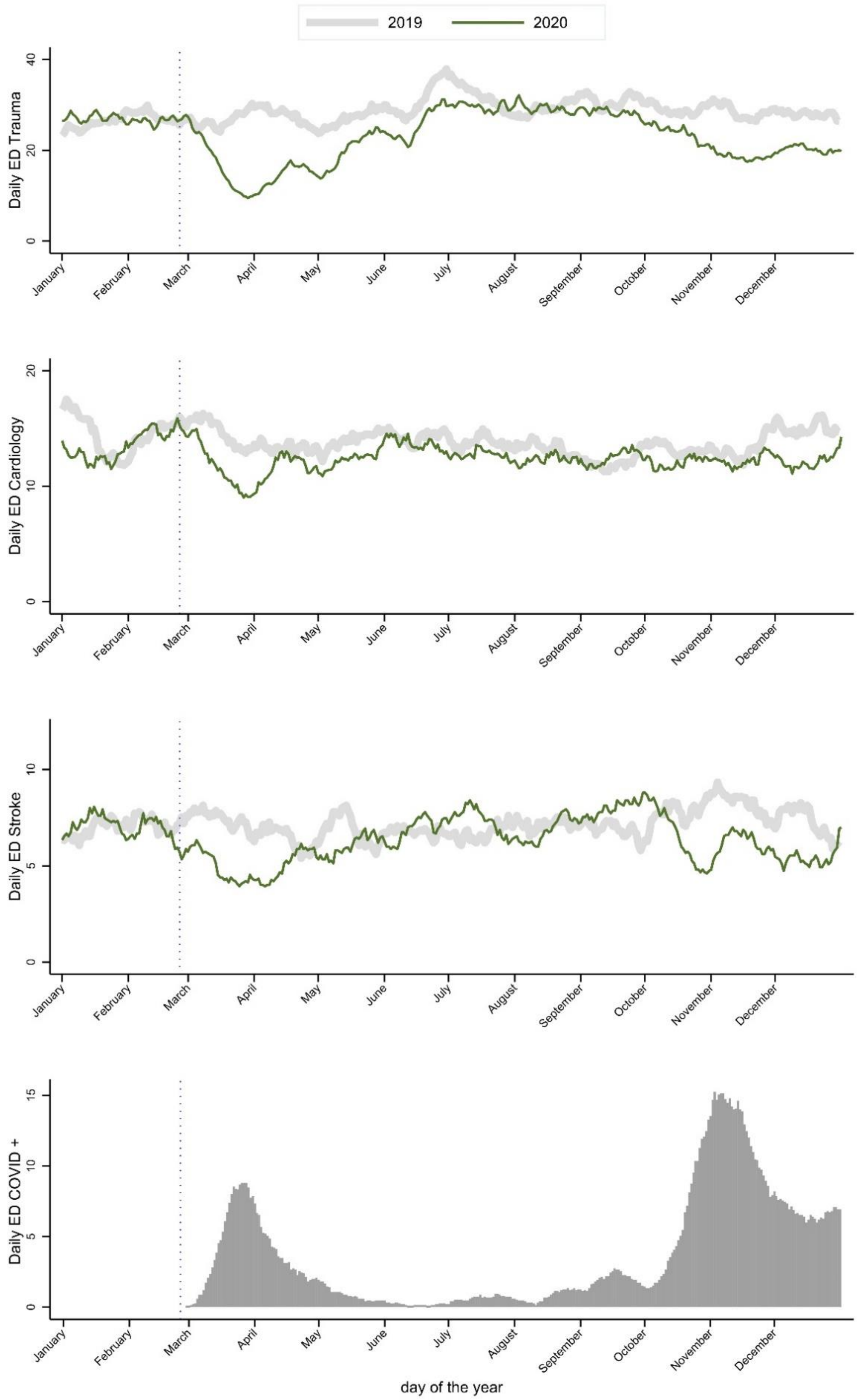

eSupplement 5. Time series by daily ED specific activity at Lausanne University Hospital (GIB, diabetes, allergy).

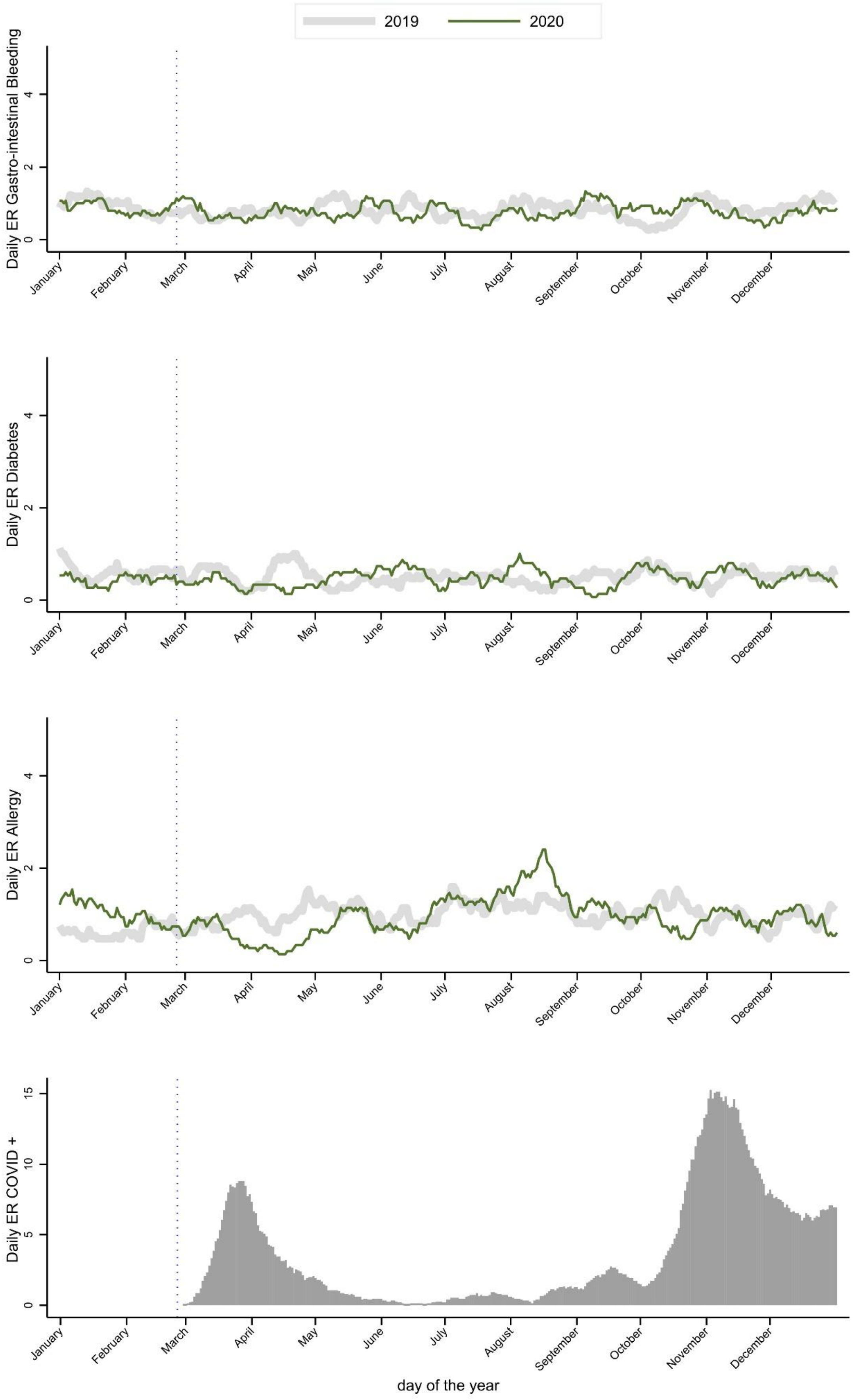

For peer review only - http://bmjopen.bmj.com/site/about/guidelines.xhtml

eSupplement 6. Time series by ED length of stay and waiting time at Lausanne University Hospital.

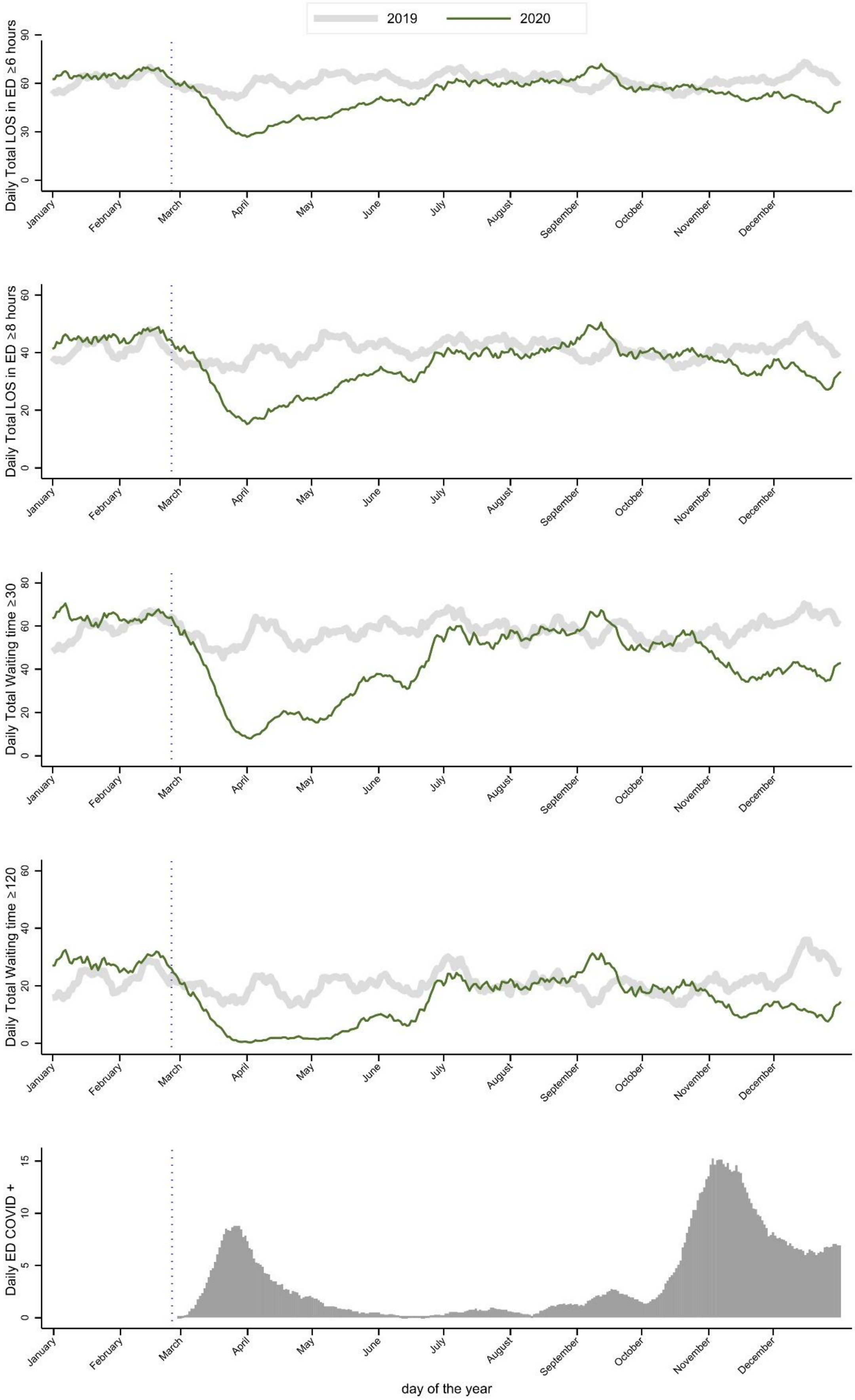

STROBE Statement—Checklist of items that should be included in reports of ***cross-sectional studies***

	Item No	Recommendation	Page No
**Title and abstract**	1	(*a*) Indicate the study's design with a commonly used term in the title or the abstract	1
		(*b*) Provide in the abstract an informative and balanced summary of what was done and what was found	2
**Introduction**			
Background/rationale	2	Explain the scientific background and rationale for the investigation being reported	4
Objectives	3	State specific objectives, including any prespecified hypotheses	4,5
**Methods**			
Study design	4	Present key elements of study design early in the paper	5
Setting	5	Describe the setting, locations, and relevant dates, including periods of recruitment, exposure, follow-up, and data collection	5
Participants	6	(*a*) Give the eligibility criteria, and the sources and methods of selection of participants	5
Variables	7	Clearly define all outcomes, exposures, predictors, potential confounders, and effect modifiers. Give diagnostic criteria, if applicable	6
Data sources/ measurement	8*	For each variable of interest, give sources of data and details of methods of assessment (measurement). Describe comparability of assessment methods if there is more than one group	6,7
Bias	9	Describe any efforts to address potential sources of bias	8
Study size	10	Explain how the study size was arrived at	NA
Quantitative variables	11	Explain how quantitative variables were handled in the analyses. If applicable, describe which groupings were chosen and why	NA
Statistical methods	12	(*a*) Describe all statistical methods, including those used to control for confounding	7,8
		(*b*) Describe any methods used to examine subgroups and interactions	7
		(*c*) Explain how missing data were addressed	8
		(*d*) If applicable, describe analytical methods taking account of sampling strategy	NA
		(*e*) Describe any sensitivity analyses	7,8
**Results**			
Participants	13*	(a) Report numbers of individuals at each stage of study—eg numbers potentially eligible, examined for eligibility, confirmed eligible, included in the study, completing follow-up, and analysed	8
		(b) Give reasons for non-participation at each stage	-
		(c) Consider use of a flow diagram	-
Descriptive data	14*	(a) Give characteristics of study participants (eg demographic, clinical, social) and information on exposures and potential confounders	8,9, table 1
		(b) Indicate number of participants with missing data for each variable of interest	Table 1
Outcome data	15*	Report numbers of outcome events or summary measures	8,9

For peer review only - http://bmjopen.bmj.com/site/about/guidelines.xhtml

Main results	16	(*a*) Give unadjusted estimates and, if applicable, confounder-adjusted estimates and their precision (eg, 95% confidence interval). Make clear which confounders were adjusted for and why they were included	Table 2, Fig 1, Fig 2
		(*b*) Report category boundaries when continuous variables were categorized	NA
		(*c*) If relevant, consider translating estimates of relative risk into absolute risk for a meaningful time period	NA
Other analyses	17	Report other analyses done—eg analyses of subgroups and interactions, and sensitivity analyses	Fig 3, Fig4, Web appendix
**Discussion**			
Key results	18	Summarise key results with reference to study objectives	9
Limitations	19	Discuss limitations of the study, taking into account sources of potential bias or imprecision. Discuss both direction and magnitude of any potential bias	11,12
Interpretation	20	Give a cautious overall interpretation of results considering objectives, limitations, multiplicity of analyses, results from similar studies, and other relevant evidence	10,11
Generalisability	21	Discuss the generalisability (external validity) of the study results	12
**Other information**			
Funding	22	Give the source of funding and the role of the funders for the present study and, if applicable, for the original study on which the present article is based	12

*Give information separately for exposed and unexposed groups.

Note: An Explanation and Elaboration article discusses each checklist item and gives methodological background and published examples of transparent reporting. The STROBE checklist is best used in conjunction with this article (freely available on the Web sites of PLoS Medicine at http://www.plosmedicine.org/, Annals of Internal Medicine at http://www.annals.org/, and Epidemiology at http://www.epidem.com/). Information on the STROBE Initiative is available at www.strobe-statement.org.

For peer review only

For peer review only - http://bmjopen.bmj.com/site/about/guidelines.xhtml
